# Crystallography of Representative MOFs Based on Pillared Cyanonickelate (PICNIC) Architecture

**DOI:** 10.3390/cryst6090108

**Published:** 2016

**Authors:** Winnie Wong-Ng, Jeffrey T. Culp, Yu-Sheng Chen

**Affiliations:** 1 Materials Measurement Science Division, National Institute of Standards and Technology, Gaithersburg, MD 20899, USA; 2 National Energy Technology Laboratory, United States Department of Energy, P.O. Box 10940, Pittsburgh, PA 15236, USA; 3 AECOM, South Park, PA 15219, USA; 4 ChemMatCARS, University of Chicago, Argonne, IL 60439, USA

**Keywords:** MOFs, Flexible Ni(CN)_4_-based metal-organic frameworks, crystallography, structure and adsorption properties

## Abstract

The pillared layer motif is a commonly used route to porous coordination polymers or metal organic frameworks (MOFs). Materials based on the pillared cyano-bridged architecture, [Ni’(L)Ni(CN)_4_]_n_ (L = pillar organic ligands), also known as PICNICs, have been shown to be especially diverse where pore size and pore functionality can be varied by the choice of pillar organic ligand. In addition, a number of PICNICs form soft porous structures that show reversible structure transitions during the adsorption and desorption of guests. The structural flexibility in these materials can be affected by relatively minor differences in ligand design, and the physical driving force for variations in host-guest behavior in these materials is still not known. One key to understanding this diversity is a detailed investigation of the crystal structures of both rigid and flexible PICNIC derivatives. This article gives a brief review of flexible MOFs. It also reports the crystal structures of five PICNICS from our laboratories including three 3-D porous frameworks (Ni-Bpene, NI-BpyMe, Ni-BpyNH2), one 2-D layer (Ni-Bpy), and one 1-D chain (Ni-Naph) compound. The sorption data of BpyMe for CO_2_, CH_4_ and N_2_ is described. The important role of NH_3_ (from the solvent of crystallization) as blocking ligands which prevent the polymerization of the 1-D chains and 2-D layers to become 3D porous frameworks in the Ni-Bpy and Ni-Naph compounds is also addressed.

## 1. Introduction

The continual rise in anthropogenic CO_2_ concentration since the dawn of the Industrial Revolution and its effect on climate change underlie the urgent need for the implementation of carbon mitigation approaches to stabilize the CO_2_ concentration in the atmosphere, which would result in a more sustainable global development [1–3]. Given the existing availability and usage of fossil fuel resources worldwide, particularly coal, fossil fuel-based energy sources are expected to remain the predominant source of energy in the foreseeable future.

Over the past twenty years, a great number of possible solid sorbents have been reported throughout the literature. Porous materials which offer a wide range of compositions and structures suitable for adsorption and capture of CO_2_ [4] near atmospheric pressure and over various temperature ranges include zeolites [5,6], activated carbon [7], smectites [8], oxide materials such as calcium oxide [9], lithium zirconates [10], and hydrotalcites [11]. There are also a vast number of reported metal organic frameworks (MOFs) or coordination polymer compounds [12–45] which show diverse properties for reversible CO_2_ adsorption over a range of pressure and temperature.

MOFs consist of metal centers and/or metal clusters connected by organic linkers, forming 3-D porous structures with 1-D, 2-D, or 3-D channel systems. The field of MOF research has been rapidly expanding in the past decade. According to Zhou and Kitagawa [12], the surge of MOF research in recent years has been due to five factors: (1) advances in cluster chemistry; (2) maturation of organic synthesis (ligand design and post-synthetic modification on linkers); (3) improvements for evaluation of sorption and structural properties; (4) interdisciplinary growth of MOFs, and (5) their expanding potential in applications.

The goals of this article are to give a brief description of flexible MOFs, with emphasis on the flexible PICNICs, and to discuss the crystallography of flexible and rigid PICNICs based on our recent crystallographic investigations on five Ni(CN)_4_-based compounds. A gas sorption study of a selected compound is also included.

### 1.1. Flexible Metal Organic Frameworks (MOFs)

Studies of flexible coordination polymers have been pioneered by Professor Kitagawa’s group at Kyoto University [46–49]. Since 1990, they have synthesized a great number of porous coordination polymers, providing a variety of properties. In their 2005 papers [47,48], they named the inorganic porous coordination polymers that are structurally flexible and possess selective adsorption characteristics along with the potential to withstand greater external stress than the third-generation coordination polymers (or MOFs). The features of the third-generation MOFs include their unique ability to undergo structural changes (with cell changes) during adsorption and desorption of guests between a closed (minimal porosity) and an open (high porosity) structure. These materials undergo reversible structural changes in response to external stimuli such as a guest inclusion.

Flexible MOFs open up an exciting new research area for the investigation of structure-property relationships in materials science. The flexible behavior in these materials occurs through several different mechanisms [50]. One type of flexible MOF occurs in multi-interpenetrating frameworks, wherein one framework shifts with respect to the others, thereby closing or opening the pores [41–43]. Another type of flexible MOF has flexible frameworks from adaptable supports [44,45], such as guest-host interactions that lead to very large swelling or breathe under external stimuli including temperature, pressure, and gas or solvent adsorption. For example, μ-53(Al, Cr) is known for its large reversible breathing amplitude between the hydrated and anhydrous form. A vanadium-based MOF, VO-(biphenyl-4,4’-dicarboxylate), which adopts an expanded μ-47 structure type, was found to display a distinct breathing effect (existence of both narrow pore and large pore forms) and has a high CO_2_ adsorption capacity [51]. Other flexible MOF structures such as Zn_2_(bpdc)_2_(bpee)]·2DMF (where bpdc = 4,4’-biphenyl dicarboxylate and bpee = 1,2-bis(4-pyridyl)ethylene) [52], and ([(Ni_2_L_4_)(bpdc)]·14H_2_O) [53] can reversibly restore and collapse their pore structure by adsorption of gases at high pressure [52].

The reversible structural changes in flexible MOFs occurring as a response to external stimuli such as a guest inclusion have been identified by the appearance of a step (gate opening) and a hysteresis in the adsorption/desorption measurements. In addition, some of these MOFs have unusual uptake behavior and preferential adsorption of one gas over the others. This is termed “selectivity” or viewed as the ratio of uptake concentrations of two gases at a given temperature and pressure. For example, Nijem et al. [54], using IR, Raman spectroscopy and density functional theory (DFT) calculations, reported their finding of the preferential adsorption of CO_2_ over N_2_ in MOF Zn_2_(bpdc)_2_(bpee) as due to the interactions of CO_2_ (quadrupole moment, polarity and stretching of the C=O bond) with the linkers, resulting in pores opening. Tanaka et al. [55] found that kinetics plays an important role in the gate-opening process in a flexible porous coordination polymer, {[Cd(benzophenone-4,4’-dicarboxylate)(4,4’-bipyridyl)]}_n_. Kauffman et al. [56] confirmed selective adsorption of CO_2_ from light gas mixture from the structural transition in the structurally dynamic porous coordination polymer, catena-bis(dibenzoylmethanato)-4,4’-bipyridyl)Ni(II), referred to as “Ni-DBMBpy” [57].

### 1.2. Ni(CN)_4_-Based MOFs (PICNICs)

Many structural changes in flexible MOFs have been observed in pseudo-1-D or 2-D materials which lack an extended covalent bridge in the third dimension. The structure of the Ni(CN)_4_-based flexible MOFs comprises extended 3-D coordination polymers which have strong coordinate covalent bridges in all three dimensions. Cyano-bridged complexes have been shown to form polymeric structures with 3-D Hofmann-like microporous frameworks [58], formed by metal-metal or metal-ligand-metal bridge connections in one, two, or three dimensions.

Back in 2001, Niel et al. [59] reported the isostructural 3-D coordination networks of the family of compounds {Fe^II^(pyrazine)[M^II^(CN)_4_]} (M = Ni, Pd and Pt). These compounds undergo cooperative spin transitions at high temperatures, and the host–guest interactions inside the pores have considerable effects on the spin crossover properties of these materials. Agusti et al. [60] observed the thermal and light-induced spin crossover phenomena in the 3-D MOF of {[Fe^II^(azpy)[M^II^(CN)_4_]}·nH_2_O (azpy = 4,4’azopyridine; M = Ni,Pd and Pt).

**Figure 1.**
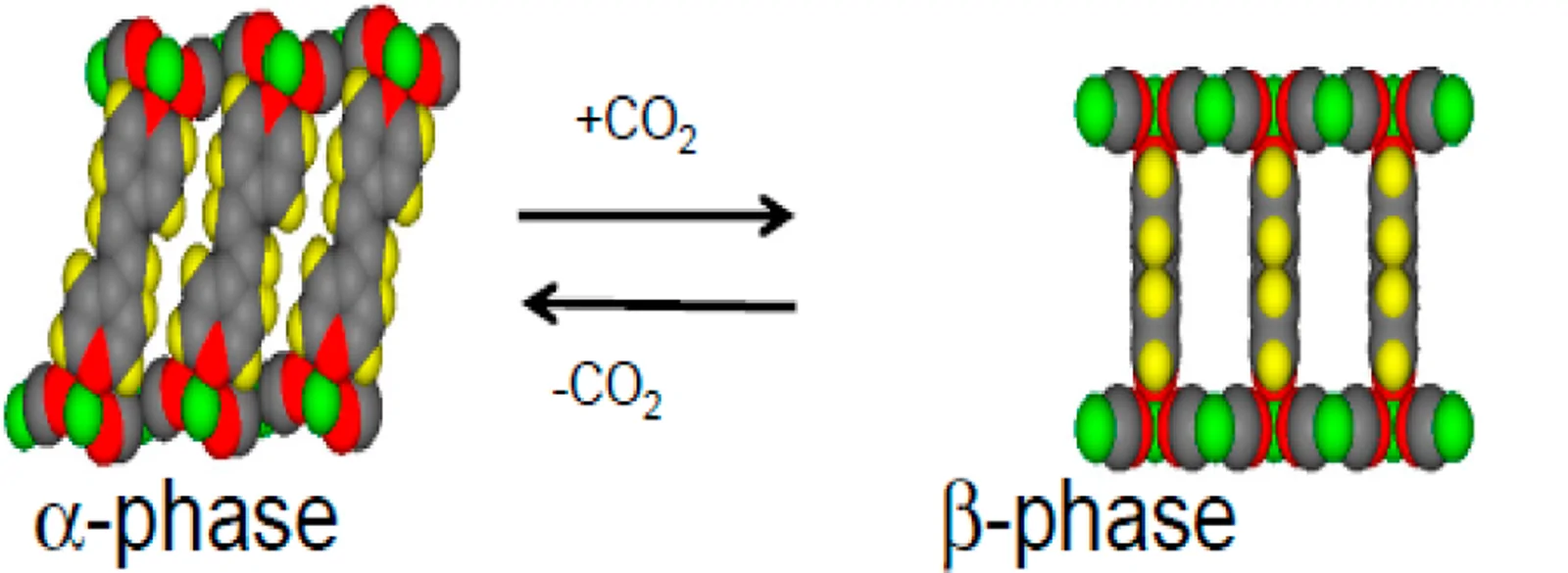
Schematic drawing demonstrating the opening/collapsing (decreasing/increasing the angle between the basal plane and the ligand) of the unit cells of Ni(L)[Ni(CN)_4_] in the presence of CO_2_.

**Figure 2.**
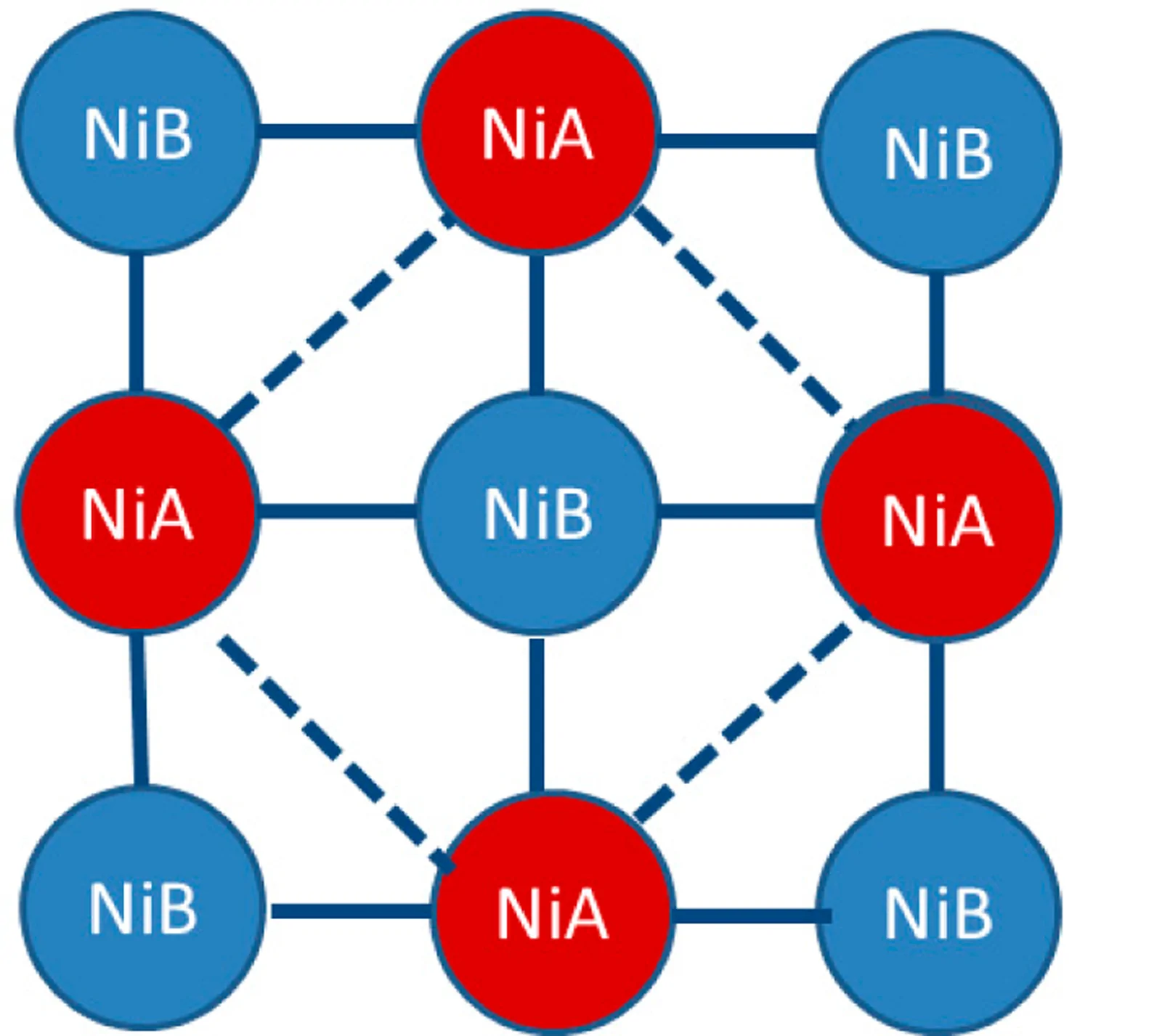
Tetracyano-nickelate square planar complex showing two different types of Nis (different coordination environment).

**Figure 3.**
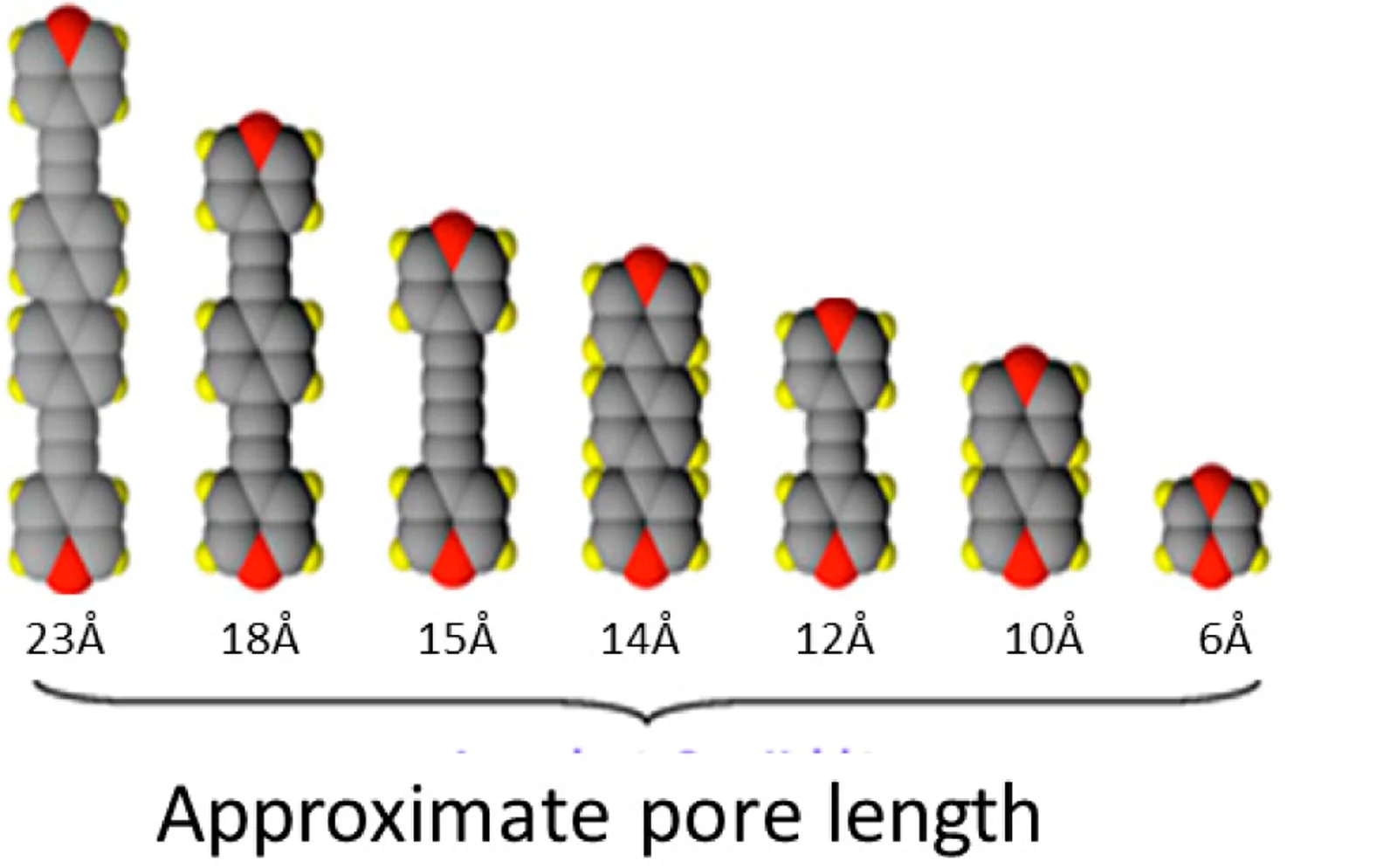
Schematics of the pillar ligands with different lengths.

In recent years, National Energy and Technology Laboratory (NETL) has been one of the leading laboratories in the area of science and engineering research of novel Ni(CN)_4_-based flexible MOFs [56,61–65]. The precursors of these flexible MOFs are typically a cyanometallate complex that acts as a ligand and a transition metal complex with available coordination sites. The schematics of the pillared layered nickel-cyanide-based MOF that consists of a flexible framework is shown in Figure 1. They are collectively called pillared cyanonickelates, or PICNICs. The PICNIC family of compounds was developed for systematic studies of how pore structure and functionality could affect CO_2_ capture and separations. These sorbents are based on 2-D planar structures made from a tetracyano-nickelate coordination compound, which is a square planar complex (Figure 2). The nitrogen-end of the cyanide ligand from NiA can also coordinate with another transition metal, in this case, Ni (NiB). NiB acts as a linear linker between the tetracyano-nickelate groups, creating a 2-D square grid network (*xy*-plane). NiB in general has a six-fold octahedral coordination and it is along the *z*-axis that the organic pillar ligand coordinates. The size of the pores of the materials can be modified by the lengths of the pillared ligands. Figure 3 shows the schematics of the pillar ligands with different lengths, where one can perceive the size of the pore as defined by the length of the ligands.

**Figure 4.**
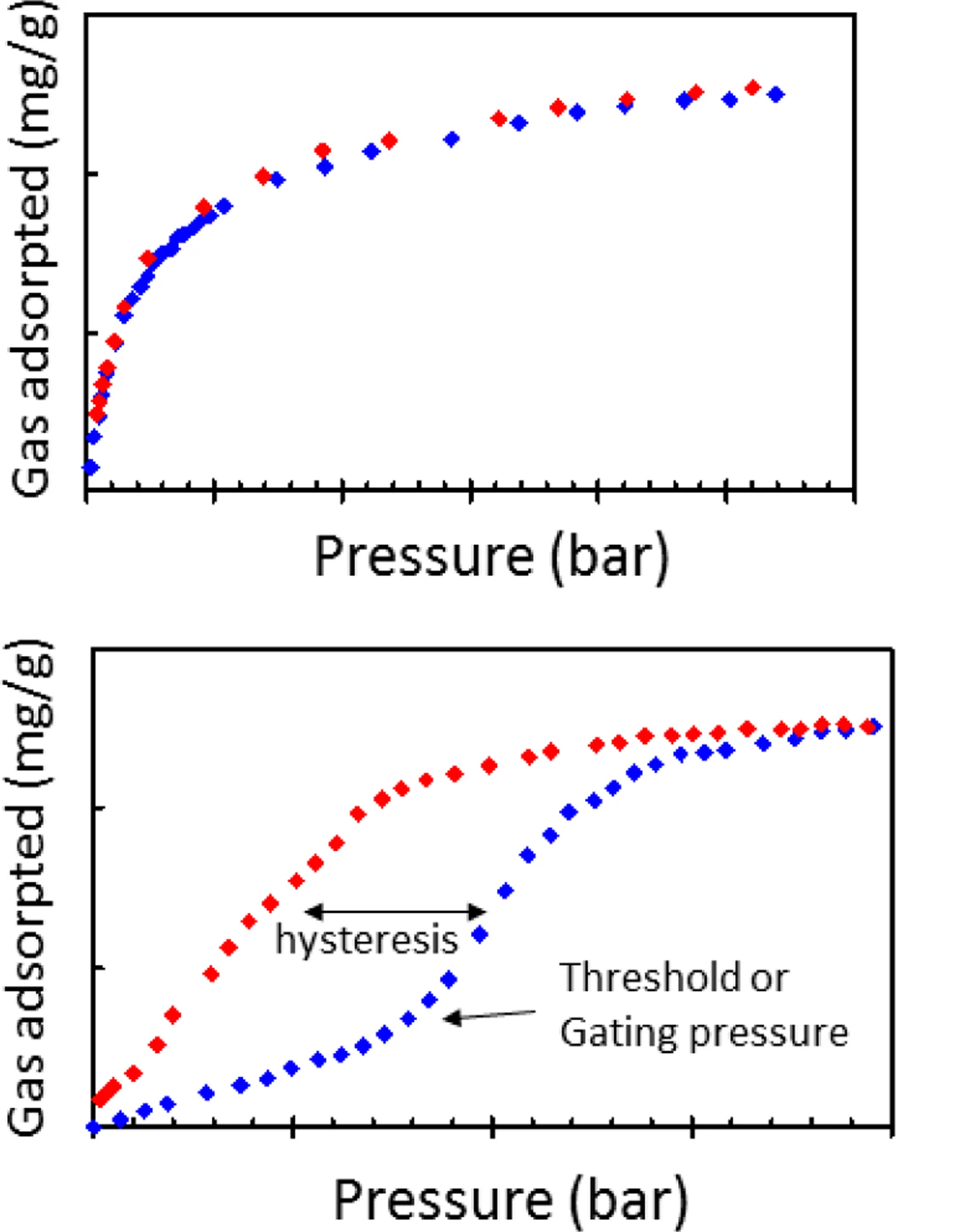
Comparing typical gas adsorption (blue)-desorption (red) cycles for a rigid porous material (**top**) and a flexible MOF (**bottom**). Adsorption in flexible sorbents is associated with a gate-opening effect at a specific threshold condition of temperature and pressure. Desorption is hysteretic.

These PICNICs have the unique ability to undergo dramatic structural changes during adsorption and desorption of guests between closed to open structure [66–77]. In some of the pillared layer systems, the pillars create a sorbent which requires a guest to maintain the structure of the pore. When this gas is removed, the sorbent collapses into a low-porosity state (Figure 1). These guests could be solvent or gas molecules. Gases such as CO_2_, CH4, or N_2_ can cause opening or collapse of the pores during the adsorption and desorption process. Many PICNICs exhibit hysteretic adsorption-desorption behavior which is often associated with the ‘gate-opening’ effect (Figure 4). They also show dynamic host-guest behavior due to structural transitions which occur between the guest-free and guest-loaded states [78–82].

The thermodynamic requirements of the induced structural change in PICNICS means that there is an activation energy that must be overcome in order to provide the energy needed to “power” the structural change. That energy is partially supplied by the energy given up during the adsorption of the gas. Different gases have different adsorption energies which can lead to selective behavior. If one gas can supply enough energy to transition the structure and the other gas cannot, then one is adsorbed and the other is not. It is thought that if gate-opening occurs at significantly different pressures for two gas components, the pores may open selectively for the preferred gas.

Detailed information of the crystal structure of PICNICs is essential for interpreting sorption data, for in-depth understanding of sorption mechanism, for modeling applications and for developing new materials for porous materials applications. In the following, our recent structural work on five selected Ni(CN)_4_-based novel materials is highlighted.

### 1.3. Five Selected Ni(Cn)_4_-Based Structural Studies

**Figure 5.**
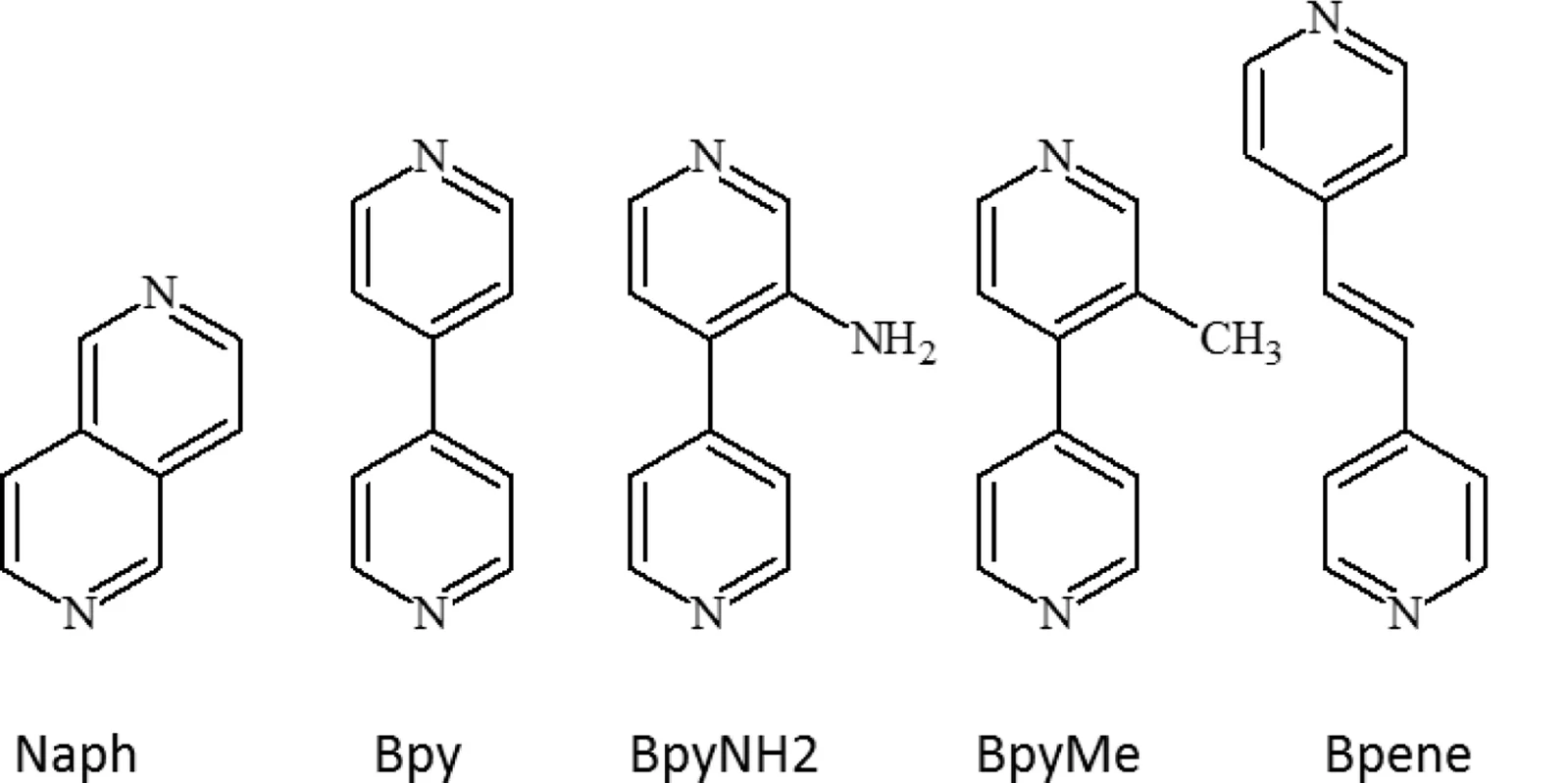
Schematic drawing of the chemical formulas for the five pillar ligands: Naph, C_8_N_2_H_6_; Bpy, C_10_N_2_H_8_; BpyNH2, C_10_N_3_H_9_; BpyMe, C_11_N_2_H_10_; and Bpene, C_12_N_2_H_10_.

Many approaches to the design of porous coordination polymers involve the use of rigid mono- or bidentate heteroaromatic N-atom donor ligands. Coordination of these ligands to the Ni ions in the Ni(CN)_4_ groups would result in a multidimensional network via a self-assembly process [83]. With the goal of synthesizing and determining the structure of representative Ni(L)Ni(CN)_4_ (L = pillar organic ligands) compounds, the five ligands that we report in this paper are shown in Figure 5 as well as listed below. Often we found that the expected structures and those that we determined are quite different due to either the presence of guest molecules in the pores of the structure or the formation of 2-D or 1-D structures instead of the expected 3-D ones.

Compound 1 which was studied in our laboratory has been reported earlier [84]:

1. 1,2-bis(4-pyridyl)ethylene, C_12_N_2_H_10_, [Bpene]
2. 3-methyl-4,4’-bipyridine, C_11_N_2_H_10_, [BpyMe]
3. 3-amino-4,4’-bipyridine, C_10_N_3_H_9_, [BpyNH2]
4. 4,4’-bipyridine, C_10_N_2_H_8_, [Bpy]
5. 2,6-naphthyridine, C_8_N_2_H_6_, [Naph]

These ligands were chosen based on their lengths and functionalities. Using ligands of different lengths offer a great tenability of structural frameworks. Functional groups on the ligands offer variable configurations as well as possible modulation of CO_2_ affinity. In this study, one of the ligands involves a 4,4’-bipyridine ring (nicknamed Bpy) as the bridging ligands while two others are Bpy-based ligands containing additional functional groups of methyl (–CH_3_) and amine (–NH_2_). The Bpene ligand consists of two pyridine rings sandwiching an ethylene group. The 2,6-naphthyridine ligand (Naph) is a relatively short ligand with two pyridine rings sharing a common edge.

## 2. Results and Discussion

As shown below in our experimental procedure, crystal growths in these samples all involve the use of a mixture of DMSO, H_2_O and NH_3_. Interestingly, though all crystals were grown from very similar reaction conditions, the structures of the materials ranged from 1-D, 2-D and 3-D coordination polymers depending on the organic bridging ligand used. In addition, one of the 3-D structures is flexible whereas two of the 3-D structures are non-flexible. These five compounds are excellent examples of the structural diversity that can arise from a relatively simple set of building blocks and minor variations in ligand structure. Table 1 gives the data collection and structure solution for the compounds we describe in the following. The structure for Ni-Bpene has been reported previously from our laboratories [84].

### 2.1. Three-Dimensional (3-D) Structures

#### 2.1.1. Ni(1,2-bis(4-pyridyl)ethylene)[Ni(CN)_4_] (Ni-Bpene)

**Figure 6.**
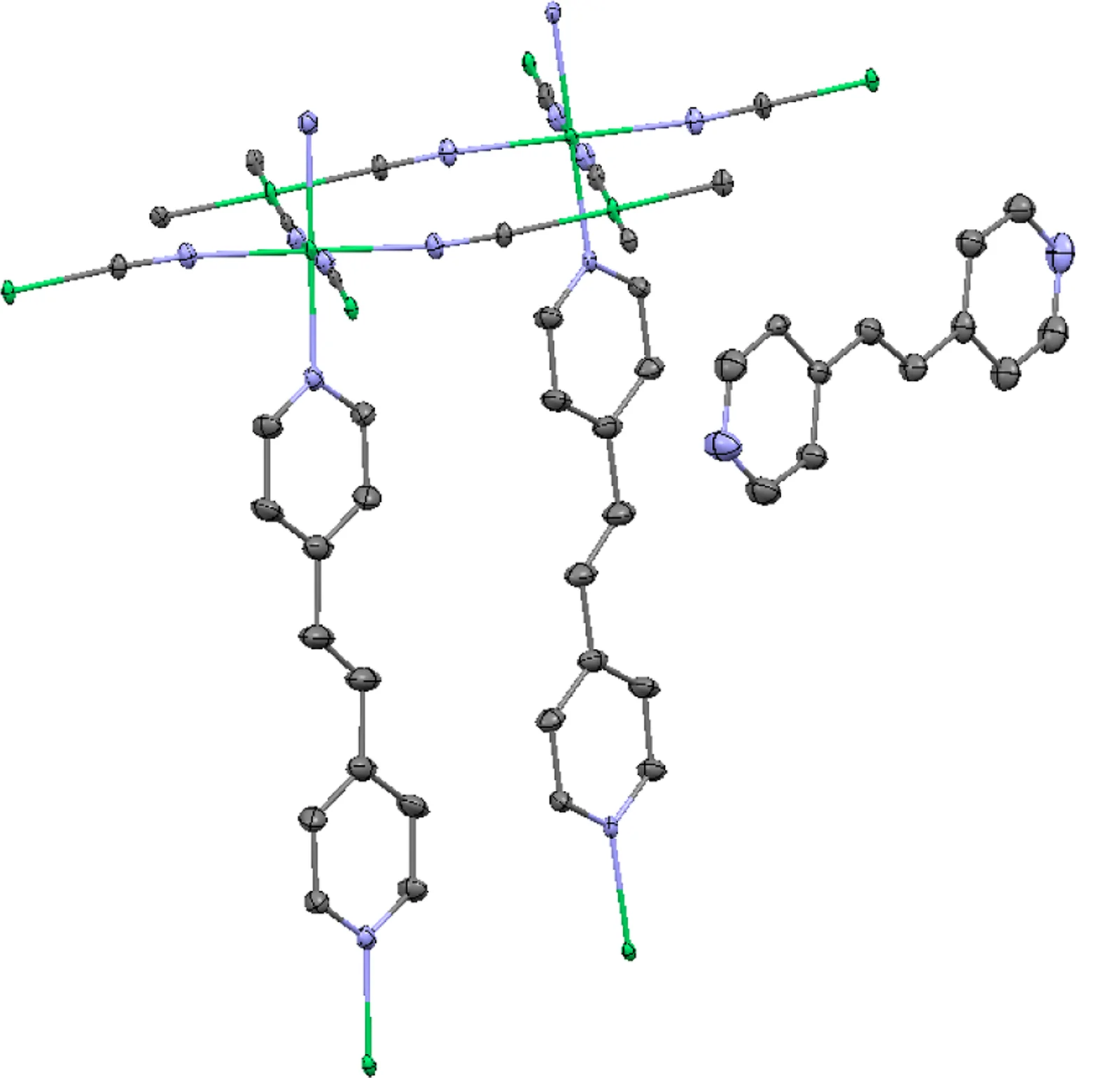
The basic motif of Ni-Bpene (C-grey, N-blue, Ni-green; probability ellipsoids drawn at 50%).

**Figure 7.**
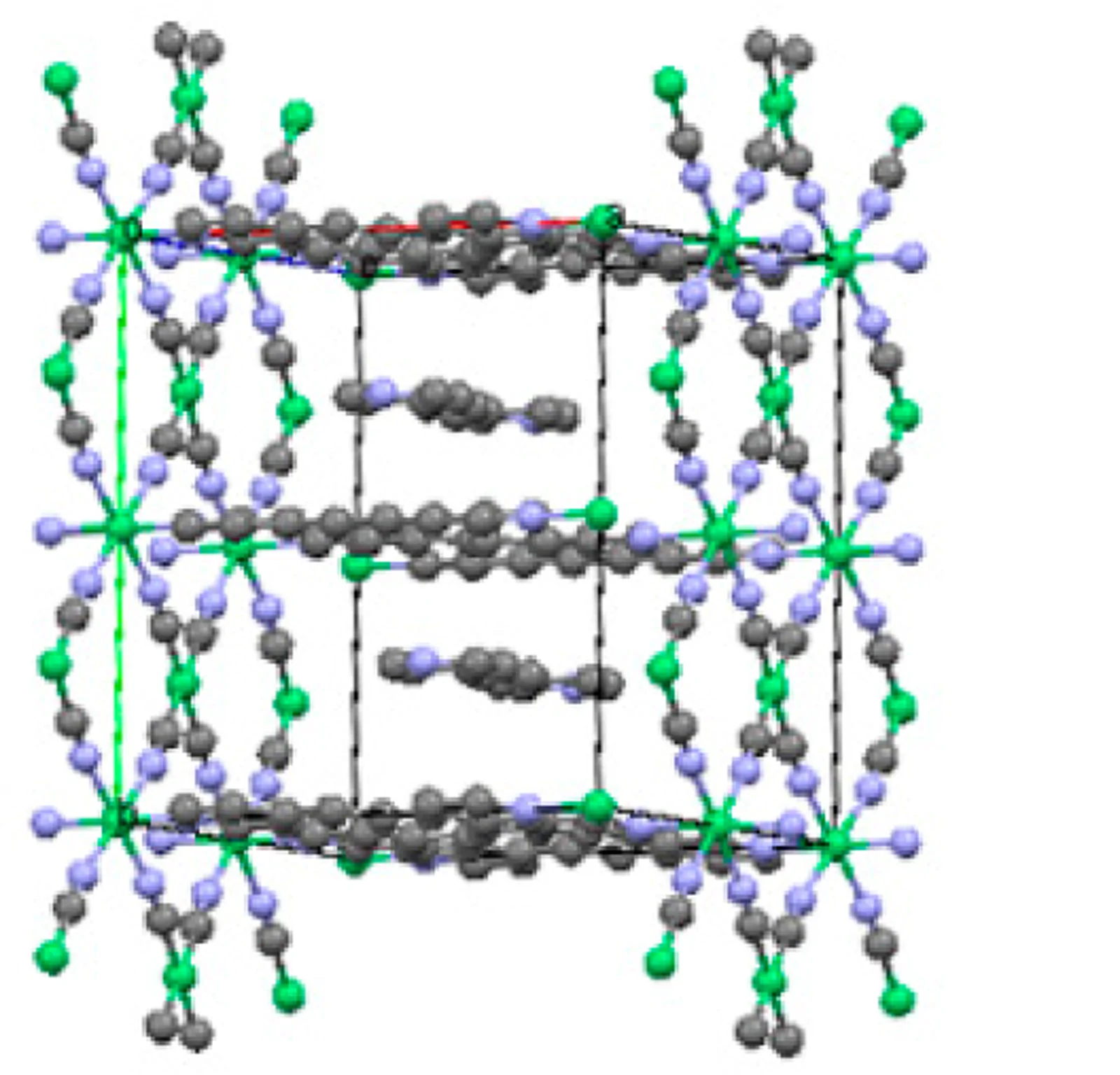
The 2-D tetracyanonickelate square grid network in Ni-Bpene (C-grey, N-blue, Ni-green).

**Figure 8.**
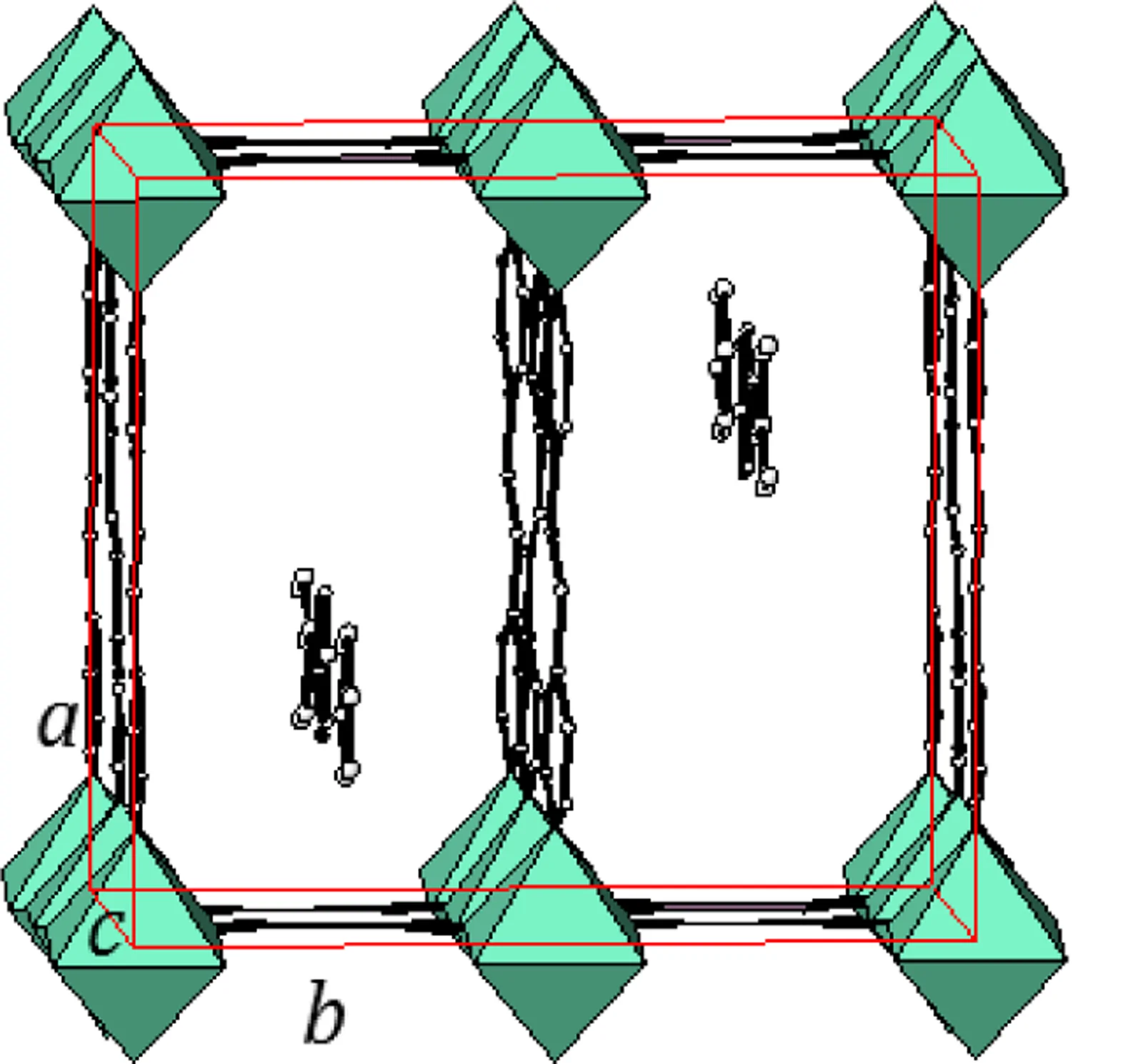
The Bpene ligands bridge the 2-D Ni[Ni(CN)_4_] sheets to form the 3-D pillared-layered structure (C-grey, N-blue, Ni-green).

In the first example, for simplicity, we refer to the sorbent as Ni-Bpene, which stands for Ni(Bpene)[Ni(CN)_4_] [84]. The expected formula without solvent molecule is C_16_H_10_N_6_Ni2. Ni-Bpene was found to adopt the 3-D Hofmann-type structure [58]. The molecular structure of the asymmetric unit with partial labeling is shown in Figure 6, and the complete labeling is given in supplementary Figure S1. The framework is based on the 2-D infinite layer-like tetracyanonickelate [Ni(CN)_4_]^2−^ planar complex [85]. Ni3 and Ni4 are each coordinated to four C atoms of the C≡N groups forming a square-planar geometry. The N ends of the C≡N ligand further coordinate to Ni1 and Ni2 which act as linear linkers between the tetracyanonickelate molecules. All C≡N groups are bridging, creating a 2-D square grid network (Figure 7). The Ni1 and Ni2 ions are octahedrally surrounded by six N atoms, four from the C≡N units and the other two from two Bpene ligands. The layers are pillared by the Bpene ligands, which occupy the axial positions of the [NiN_6_] octahedron. These Bpene ligands further bridge together the 2-D Ni[Ni(CN)_4_] sheets that form the 3-D pillard-layered structure is shown Figure 8. Ni1 and Ni2 are shown to have an octahedral environment while Ni3 and Ni4 adopt a square planar geometry. The Bpene pillar ligands and the Ni[Ni(CN)_4_] layers encase empty voids of parallelepiped shapes which are created by the absence of Bpene coordination to Ni3 and Ni4 of the square plane [Ni(CN)_4_] building units. These void spaces provide pockets for the encapsulation of guest molecules [58].

The chains that make up the 2-D Ni[Ni(CN)_4_] sheets are arranged in a zigzag manner (for example, deviations of bond angles from 180° were observed. The bond distances of Ni-N and Ni-C within the 2-D sheet are all within the expected range (Table S1). The Ni3 and Ni4 have a square planar arrangement, and the mean Ni-C (1.860 Å) and C≡N (1.153 Å) bond lengths agree with the values reported for other tetracyanonickelate salts [86]. The Ni-N distances (range from 2.0168(13) Å to 2.099 (2) Å) are substantially longer than the Ni-C distances (1.856(2) Å to 1.865(2) Å), which also conform to the literature values [87]. The six-coordination Ni are of high spin configuration because nitrogen is acting as a weak-field ligand. When the Ni atom only has four coordinates (such as that in Ni(CN)_4_) [88], the low-spin square-planar coordination of Ni^2+^ ions results in a contraction.

Unexpectedly, one also observes that free Bpene ligands that are not coordinated to any Ni atoms are also present in the cavities of the structure. Positional disorder was observed in the free ligands. Similar disorder was further observed with half of the coordinated Bpene ligand molecules in the unit cell. Apparently there is sufficient space in the unit cell so these ligands have freedom to take on more than one configuration. Also, in the Bpene ligand, the ethylene moiety (C=C double bond) that connects the two pyridyl rings can have two conformations (Figure S1). This disorder was further observed with the free ligands in the lattice as well. Solvent molecules used as solvent of crystallization, such as water and dimethylsulfoxide (DMSO), were also found inside the cavities as disordered molecules. The chemical formula with inclusion of solvent and additional ligand is C_24_H_35_N_7_Ni_2_SO_3_. The guest Bpene ligand and solvents can be removed by a combined extraction and evacuation process to provide a material activated for sorption of gases.

#### 2.1.2. Ni(3-methyl-4,4′-bipyridine)[Ni(CN)_4_], (Ni-BpyMe)

**Figure 9.**
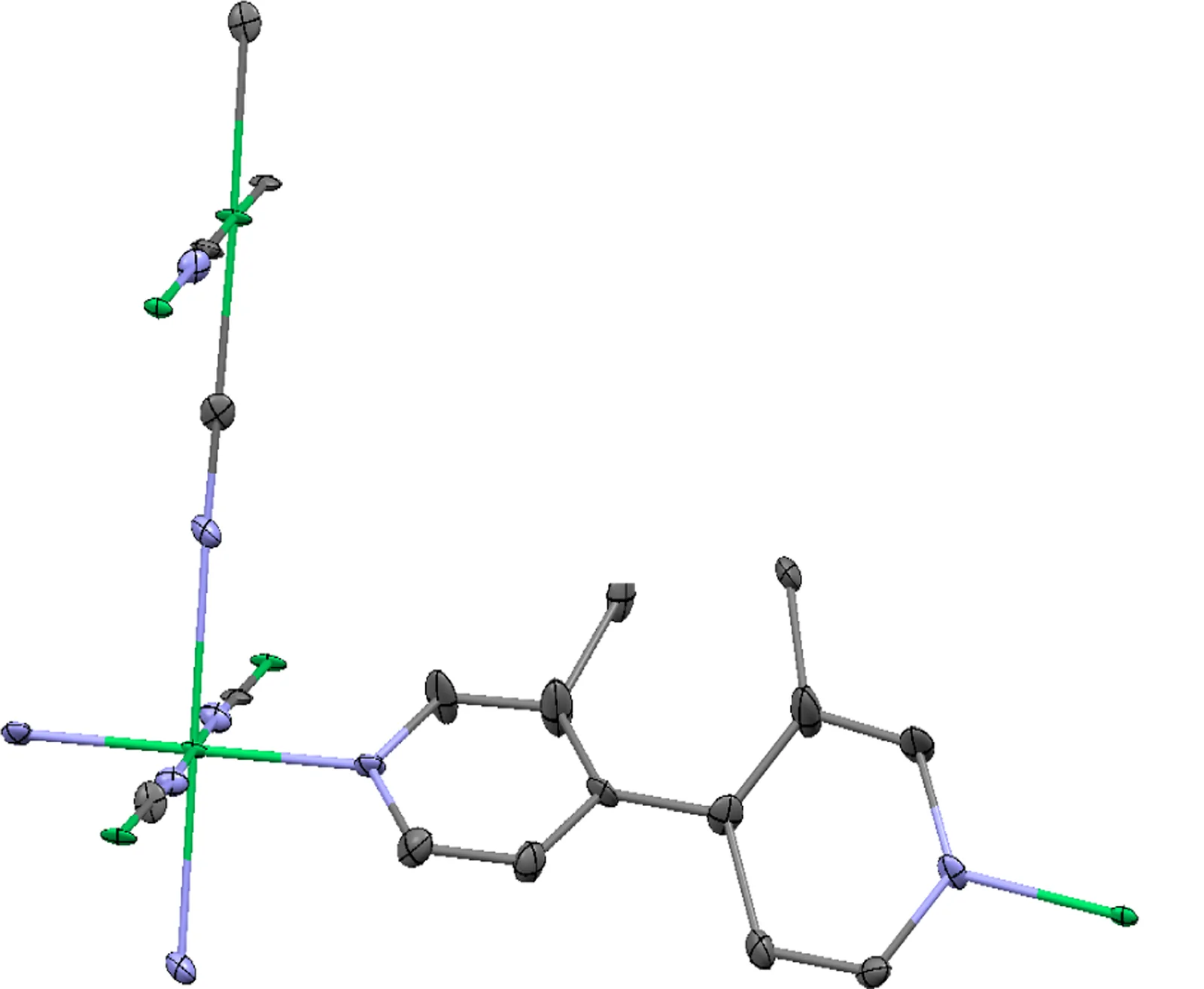
The basic motif of the Ni(BpyMe)[Ni(CN)_4_] molecules (C-grey, N-blue, Ni-green; probability ellipsoids drawn at 50%).

Tables S2–S6 give the atomic coordinates, anisotropic displacement parameters, selected pertinent bond distances, atomic coordinates for the hydrogen atoms and selected bond angles for Ni-BpyMe, respectively. Figure 9 gives the basic motif of the Ni(BpyMe)[Ni(CN)_4_] molecules. A complete labeling of the crystallographic independent unit is given in supplementary Figure S2.

**Figure 10.**
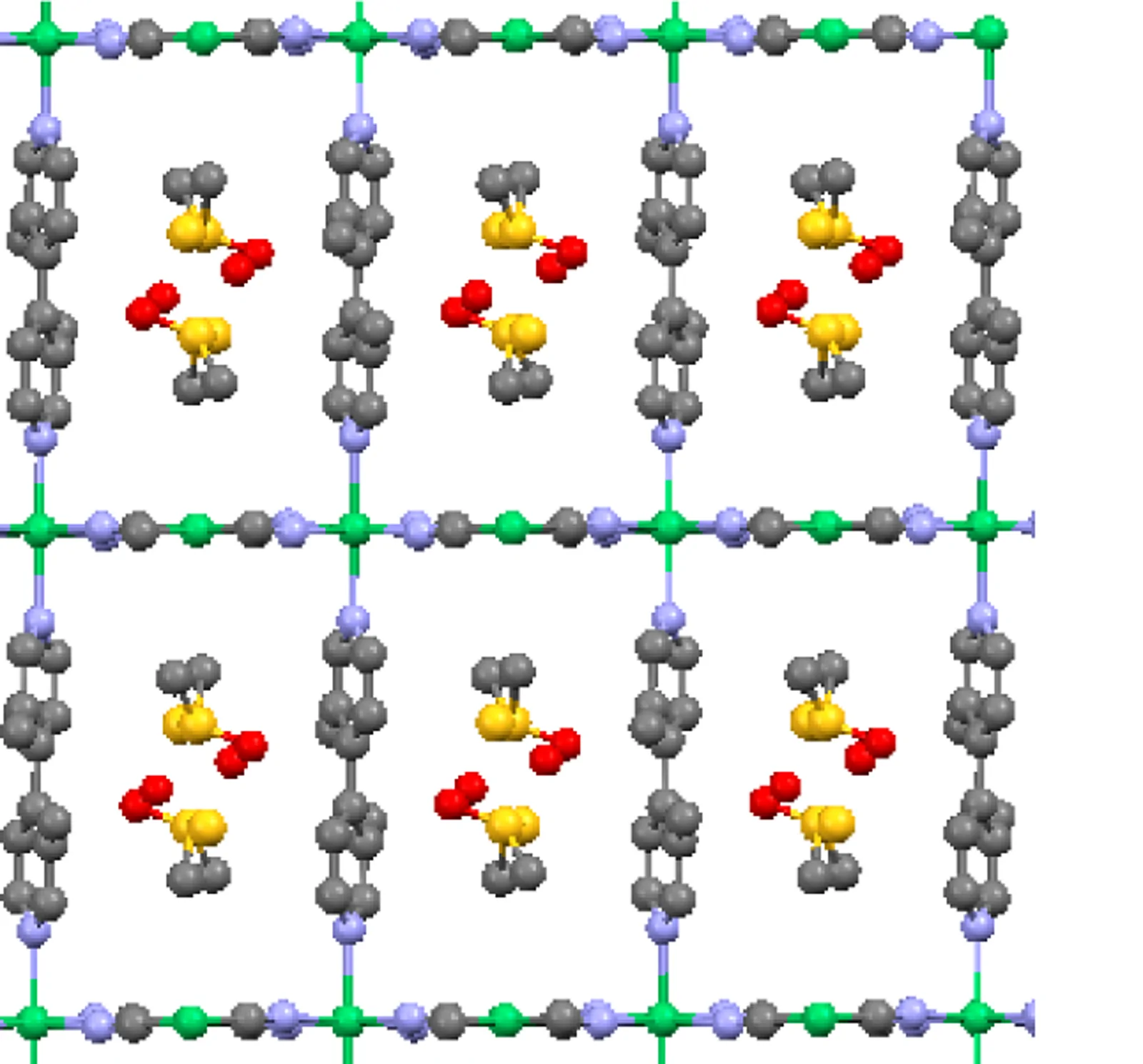
Structure of Ni-BpyMe with disordered DMSO solvent inside the rectangular cage (view along the b-axis; C-grey, N-blue, Ni-green, S-yellow, O-red).

Similar to Ni-Bpene, the Ni-BpyMe crystal is monoclinic with space group *P2/n*, *a* = 13.3483(14) Å, *b* = 7.1002(7) Å, *c* = 13.5625(14) Å, and *β* = 114.834(2)°. The structure of Ni-BpyMe consists of a 3-D net built from extended 2-D [Ni(CN)_4_] groups. These layers are connected to each other via the BpyMe ligands at the six-fold coordinated Ni2 site. Ni1 has a four-fold coordination environment. The interesting feature of this structure is that instead of locating the –CH_3_ group in one position of the pyridine ring as expected, it appears in two positions. The structure represents an average of two configurations which gives rise to the pseudosymmetry. The methyl group is distributed equally among the two pyridine rings; therefore, the occupancy of the methyl group in each pyridine ring site is 50%. The packing diagram of Ni-BpyMe is shown in Figure 10. The disordered hydrogen atom with an occupancy of 0.5 has been assigned as H4 (Table S5)

The bond distances of Ni1-N in the six-fold coordination environment (an average of 2.083 Å) are longer than that of the four-fold coordinated Ni2-C (an average of 1.862 Å), as expected. The average CN distance was found to be 1.152 Å. Bond distances within the bipyridine rings are as expected (Table S4). The two pyridine rings are not coplanar and form a dihedral angle of 45° to avoid steric hindrance between the two rings due to the bulky methyl group.

Similar to Ni-Bpene, disordered solvent of crystallization, DMSO, was also found in the pores (channels) of the structure (Figure 10). In each cluster of DMSO, there are two disordered positions of S (S1a and S2b) and O atoms (O1a and O2b), but the two carbon positions are not disordered (C9 and C10). The two resolved DMSO molecules are S1aO1aC9C10 and S2bO2bC9C10 (hydrogens not included). It is likely that highly disordered water molecules are also in the channel. The chemical formula of the compound is therefore C_19_H_21_N_6_Ni_2_O_2_S_2_.

Unlike that in Ni-Bpene, there is no extra free ligand entrapped in the lattice. It is expected that after the removal of DMSO, the voids in the structure will be able to accommodate guest molecules such as CO_2_. It is possible that the presence of the –CH_3_ groups would play a role in interacting with the guest molecules.

**Figure 11.**
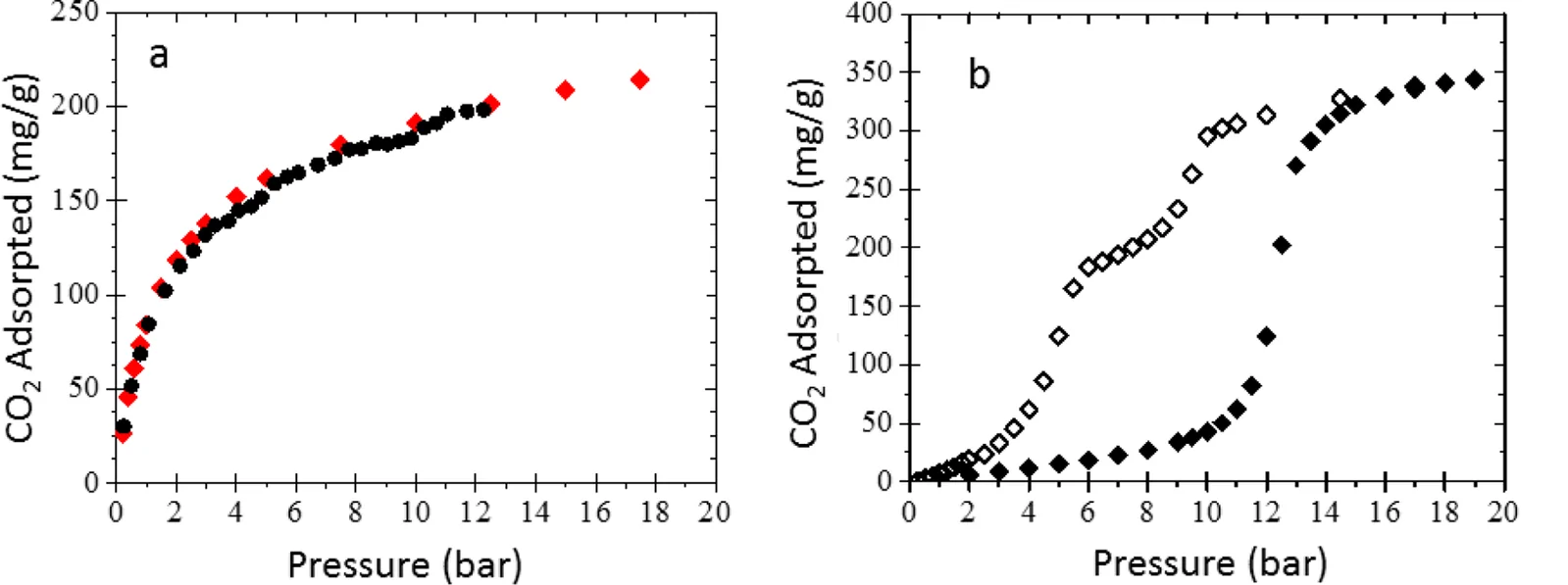
Comparing CO_2_ adsorption data at 30 °C for Ni-BpyMe powder (black) and single-crystal (red) samples. Plot (**b**) is shown to contrast the adsorption (solid)–desorption (open) behavior for CO_2_ at 30 °C on the flexible PICNIC Ni-Bpene to that of the rigid Ni-BpyMe sample, plot (**a**).

**Figure 12.**
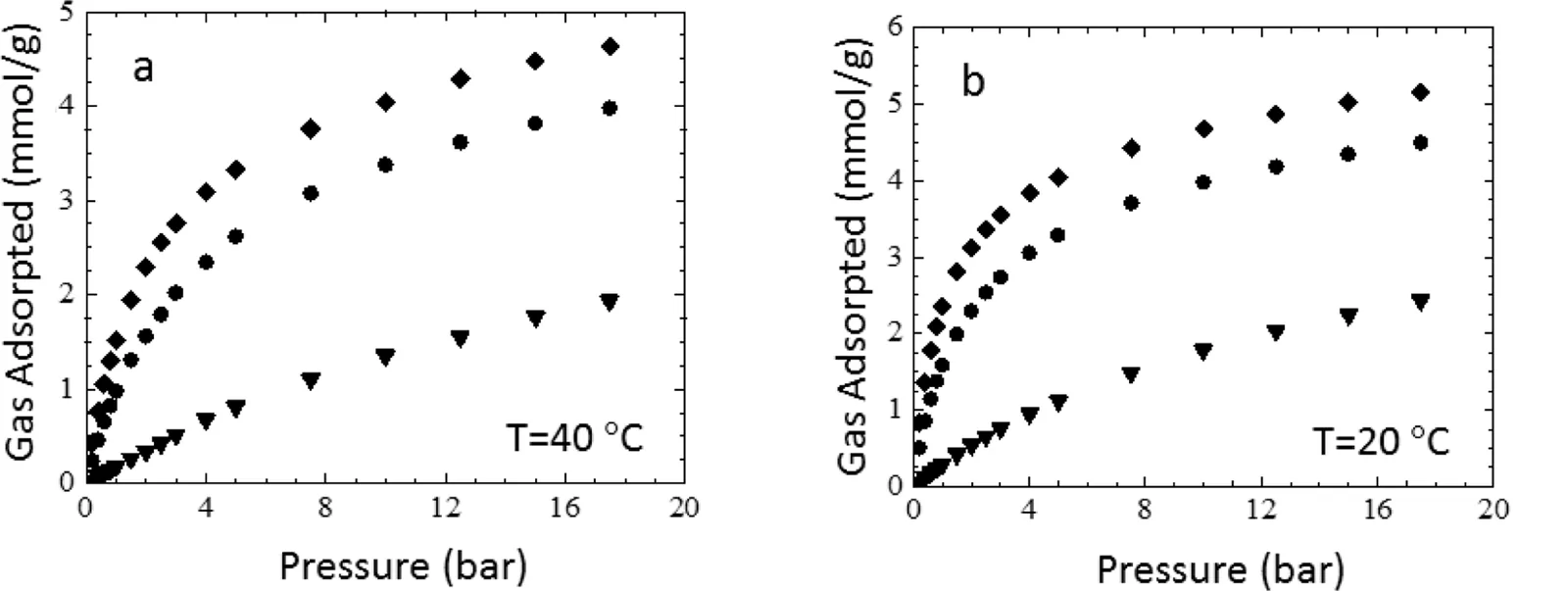
Adsorption data for CO_2_ (diamonds), CH_4_ (circles) and N_2_ (triangles) at 40 °C (**a**) and at 20 °C on a crystalline sample of Ni-BpyMe (**b**).

The gas adsorption properties of a large number of PICNIC derivatives were recently reported [61]. These materials were prepared by a heterogeneous ligand intercalation reaction which gave only powder products and hence detailed structural characterization was lacking. The Ni-BpyMe crystal sample prepared using the current technique (after extraction of guest DMSO with methanol and evacuation) gives the same CO_2_ adsorption isotherm as the powder sample prepared previously showing that the material can be prepared by two completely different synthetic techniques. Figure 11a gives the adsorption curves for Ni-BpyMe for both the crystal (red powder and black single crystals) and powder samples at 30 °C. In contrast to Ni-Bpene (Figure 11b), Ni-BpyMe gives a typical Type I adsorption isotherm with no indication of a structure transition over the measured range of temperature and pressures. Additional gas adsorption curves for crystalline Ni-BpyMe with CH_4_, N_2_ and CO_2_ are shown in Figure 12 as a function of temperature. As is typically observed with porous materials, the trend of these curves indicate that the crystal has the highest adsorption capacity for CO_2_ due to the fact that CO_2_ molecules (having quadrupole moments) have relatively stronger interaction (although weak intermolecular forces) with the BpyMe molecules than that with the CH_4_, and N_2_ molecules, respectively. As the temperature increases from 20 °C to 40 °C, the adsorption amount decreases relatively. This behavior is consistent with a physisorption mechanism.

#### 2.1.3. Ni(3-amino-4,4’-bipyridine)[Ni(CN)_4_], (Ni-BpyNH2)

**Figure 13.**
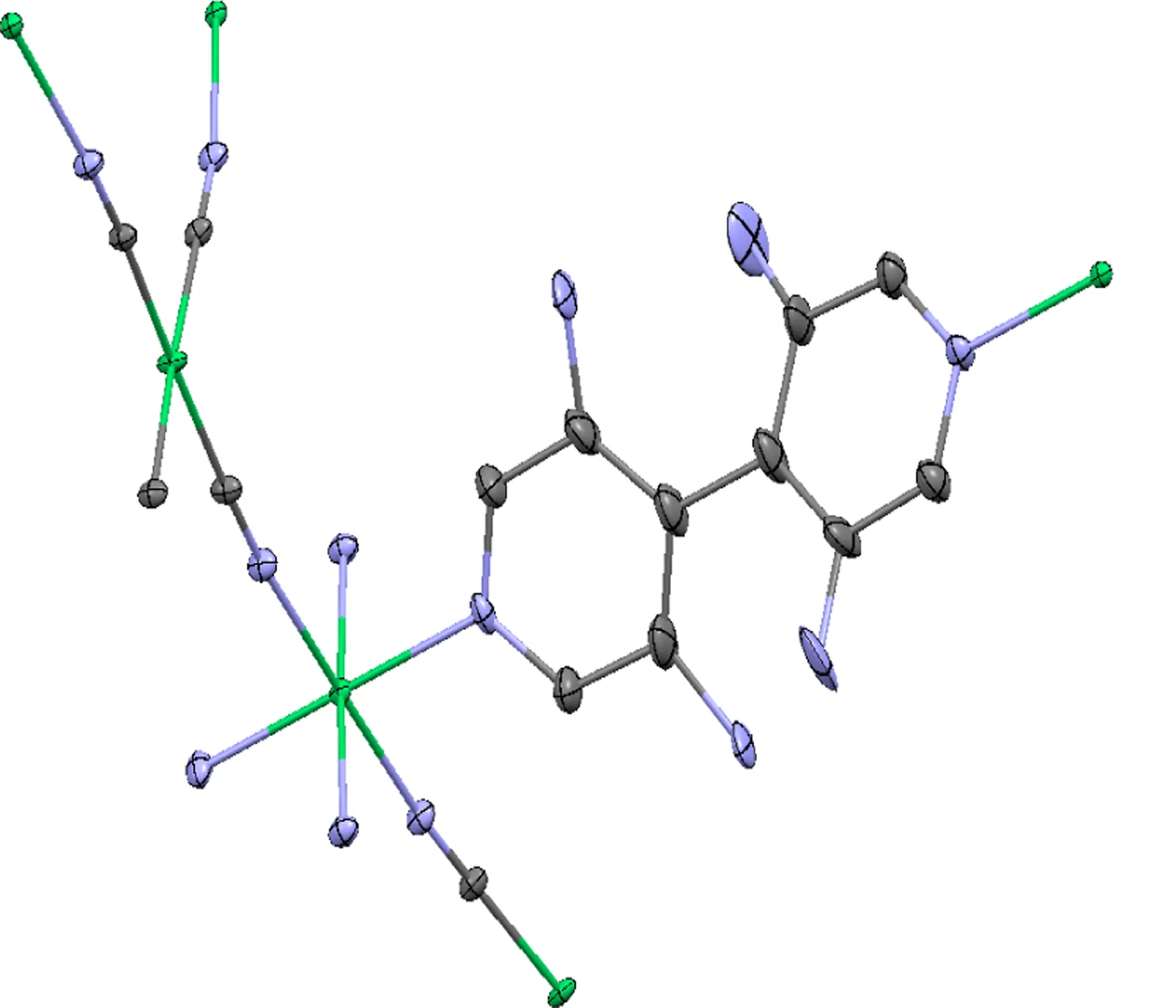
The basic motif of the Ni(BpyNH2)[Ni(CN)_4_] molecules (C-grey, N-blue, Ni-green; probability ellipsoids drawn at 50%).

Tables S7 to S11 give the atomic coordinates, anisotropic displacement parameters, selected pertinent bond distances, atomic coordinates for the hydrogen atoms and selected bond angles, respectively, for Ni-BpyMe. Figure 13 gives the basic motif of the Ni(BpyNH2)[Ni(CN)_4_] molecules. A complete labeling of the crystallographic independent unit is given in supplementary Figure S3. It was difficult to grow high-quality Ni-BpyNH2 crystals, as reflected partly on the final relatively high residual values.

The compound was found to be orthorhombic having a space group *Cmca*. The unit cell dimensions were determined to be *a* = 14.7000(5) Å, *b* = 22.6879(7) Å, *c* = 13.8028(4) Å, *V* = 4603.4(2) Å^3^, and *Z* = 8. The *b*-axis is much longer than that of *a*- and *c*- and that is where the long axis of the ligand BpyNH2 aligns.

**Figure 14.**
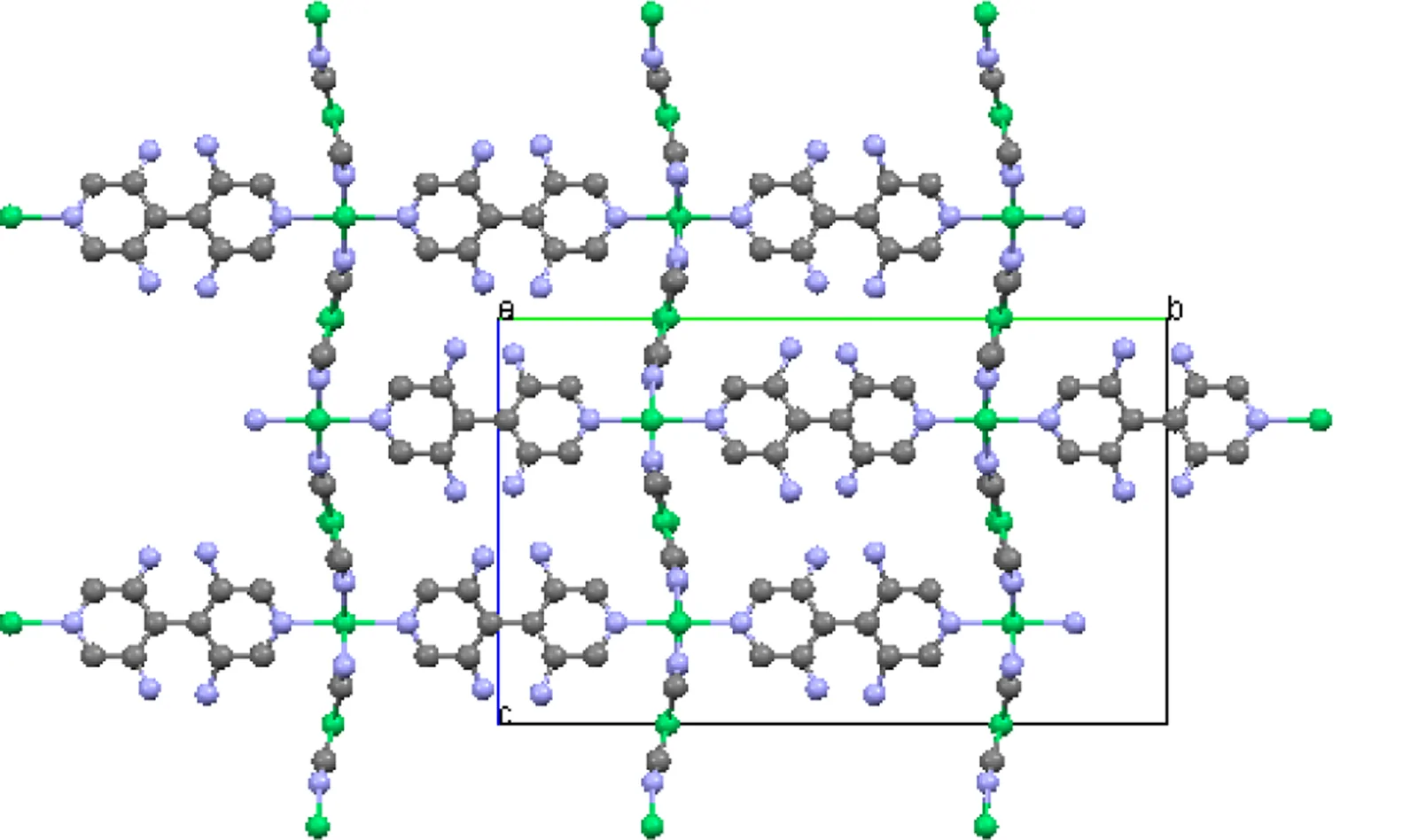
2-D Ni(CN)_4_ wavy net connecting to each other via the Ni-BPyNH2 ligands to form a 3-D structure (C-grey, N-blue, Ni-green), viewing along *a*-axis.

**Figure 15.**
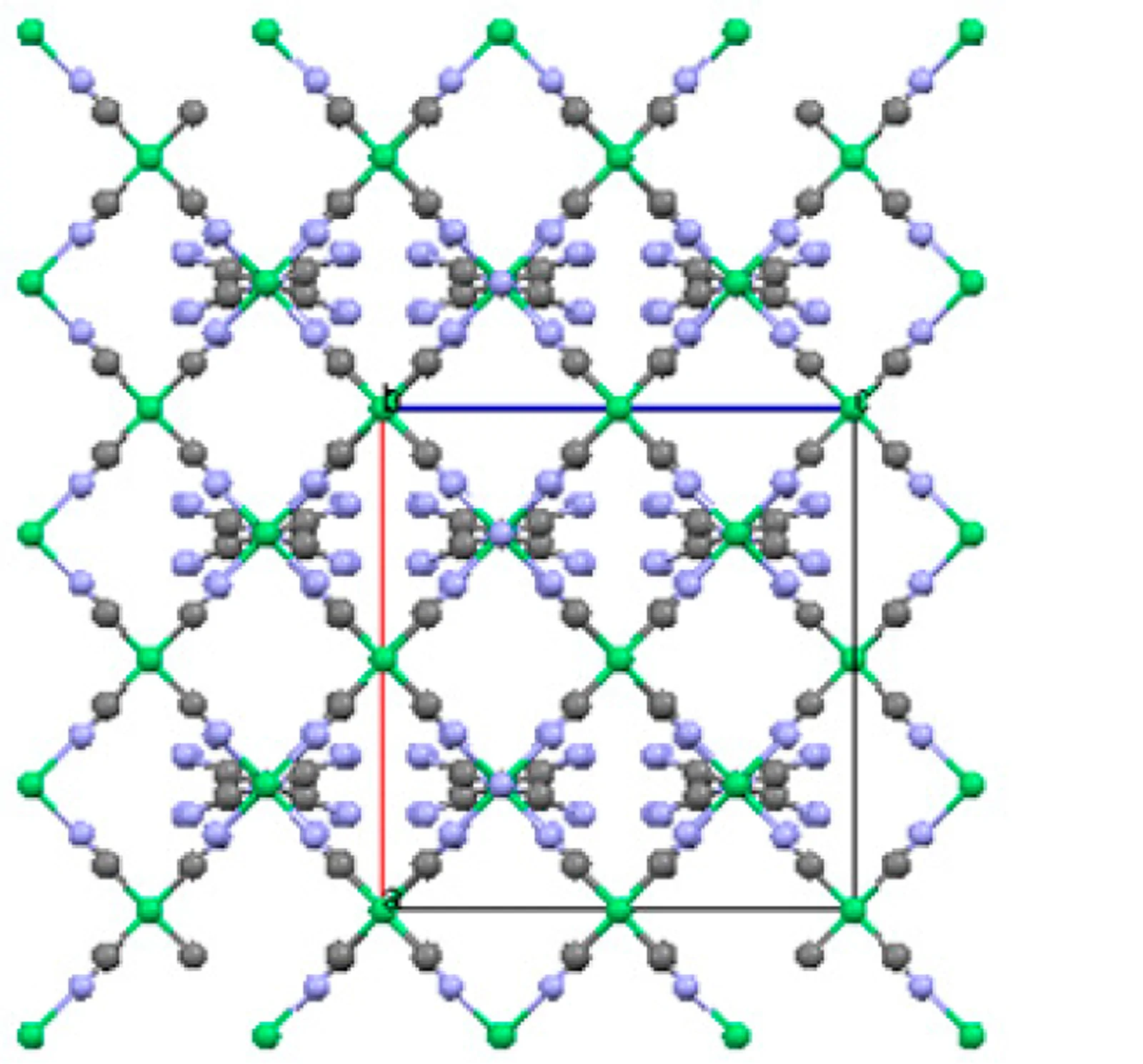
Ni-BPyNH2 square net view down the *b*-axis of the molecule (C-grey, N-blue, Ni-green).

Ni-BpyNH2 forms a 3-D network, with a 2-D Ni(CN)_4_ square net connecting to each other via the BpyNH2 ligands. Figure 14 gives the 3-D packing diagram of the structure, viewing along the *a*-axis. It is clear that the fundamental structure is similar to the Ni-Bpene and Ni-BpyMe structures where parallelepiped-shaped cavities were enclosed by the 2-D Ni(CN)_4_ net and the BpyNH2 ligands. The 2-D Ni(CN)_4_ net is connected to each other via the bonding of the pyridine “N” atom to Ni2. The Ni2 atom is of six-fold coordination to N with relatively long Ni2-N distances (average of 2.079 Å) as compared to the four-fold coordination Ni1-C distances (average of 1.860 Å). The Ni(CN)_4_ net is arranged in a wave-like fashion. Figure 15 gives the “projected view” of the net where the NH2 groups are pointing towards the center of the pores (hydrogen atoms not shown in the figure).

Similar to Ni-BpyMe, one of the features of the Ni-BPyNH2 system is that the amine functional group, –NH_2_, has been solved using a disordered model resulting from an average of four configurations, giving rise to the observed pseudosymmetry. The NH_2_ group is found in the *m*-position relative to the N atom of the pyridine ring. It has one-quarter site occupancy in each of the four *m*-positions. In other words, the NH_2_ group has 25% probability of being at one of the four possible *m*-positions. Furthermore, similar to the structure of Ni-BpyMe, the two pyridine rings in the ligands are not coplanar; they are essentially perpendicular to each other to avoid large steric hindrance. The two disordered hydrogen atoms with an occupancy of 0.75 have been assigned to H4 and H7 (Table S10).

Based on the structure of Ni-BpyMe, it is expected that the solvent molecules DMSO are also entrapped in the channel. DMSO molecules were indeed located in two independent sites. However, these solvent molecules are highly disordered in the pores. Similar to Ni-Bpene and Ni-BpyMe, it is expected that when the disordered solvent molecules are extracted, they can be replaced by guest molecules. The relatively high residual electron density in the vicinity of the disordered DMSO solvent molecule is partly due to the inadequate displacement model (anisotropic ellipsoids) describing the thermal motions. The overall structure of the Ni-BpyNH2 network indeed gives important information for future guest molecule exchange studies.

In this system, no extra BpyNH2 ligand molecules were found to be inside the cavities of the structure. The chemical formula of the compound is therefore C_18_Ni_2_S_2_O_2_N_7_H_21_. Efforts to obtain gas adsorption data on the crystalline Ni-BpyNH2 sample were hampered by the inability to obtain a sufficient quantity of pure material since the crystals had to be mechanically separated from a significant amount of polycrystalline impurity.

### 2.2. Two-Dimensional (2-D) Structure

#### Ni(4,4’-bipyridine)[Ni(CN)_4_], (Ni-Bpy)

**Figure 16.**
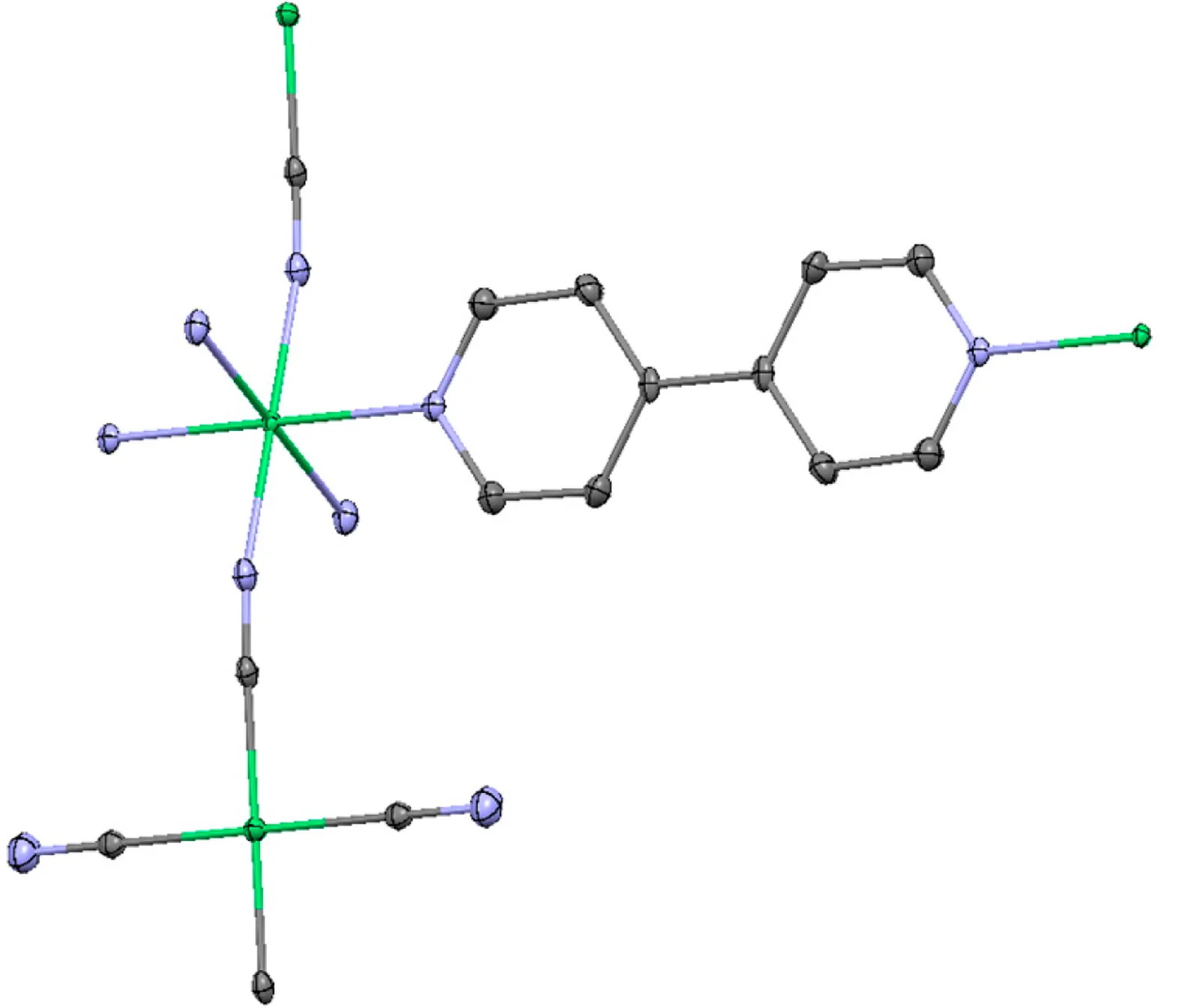
The basic motif of the Ni(Bpy)[Ni(CN)_4_] molecules (C-grey, N-blue, Ni-green; probability ellipsoids drawn at 50%).

Tables S12 to S16 give the atomic coordinates, anisotropic displacement parameters, selected pertinent bond distances, atomic coordinates for the hydrogen atoms and selected bond angles, respectively, for Ni-Bpy. Figure 16 gives the basic motif of the Ni(Bpy)[Ni(CN)_4_] molecules. A complete labeling of the crystallographic independent unit is given in supplementary Figure S4.

The structure of the compound Ni (4,4’-bipyridine)[Ni(CN)_4_], or Ni-Bpy, was determined to be a 2-D metal organic cyanide-bridged framework instead of having a 3-D network. The resulting chemical formula is [Ni(CN)_4_Ni(C_10_H_8_N_2_)(NH_3_)_2_], or Ni_2_C_14_N_8_H_14_, instead of the expected Ni_2_C_14_N_6_H_8_. The crystal was found to be orthorhombic *Pnnm*, *a* = 7.2641((9) Å, *b* = 9.9285(12) Å, *c* = 11.4008(14) Å. The structure can be considered as extended 2-D sheets, while interacting with the neighboring sheets via van der Waals’ forces (Figure 16). The structure is essentially composed of –Ni-4,4’-Bpy-Ni-4,4’Bpy-Ni chains linked by [Ni(CN)_4_]^2−^ anions. The six-fold coordination environment of the Ni^II^ includes two NH_3_ groups (in place of two –C≡N groups), while the coordination environment around the four-fold Ni^I^ is completed by two terminal –C≡N groups and two other –C=N- in the chain. As a result of these unexpected coordinates, cross-link bridging is no longer possible, and a 3-D structure is excluded.

**Figure 17.**
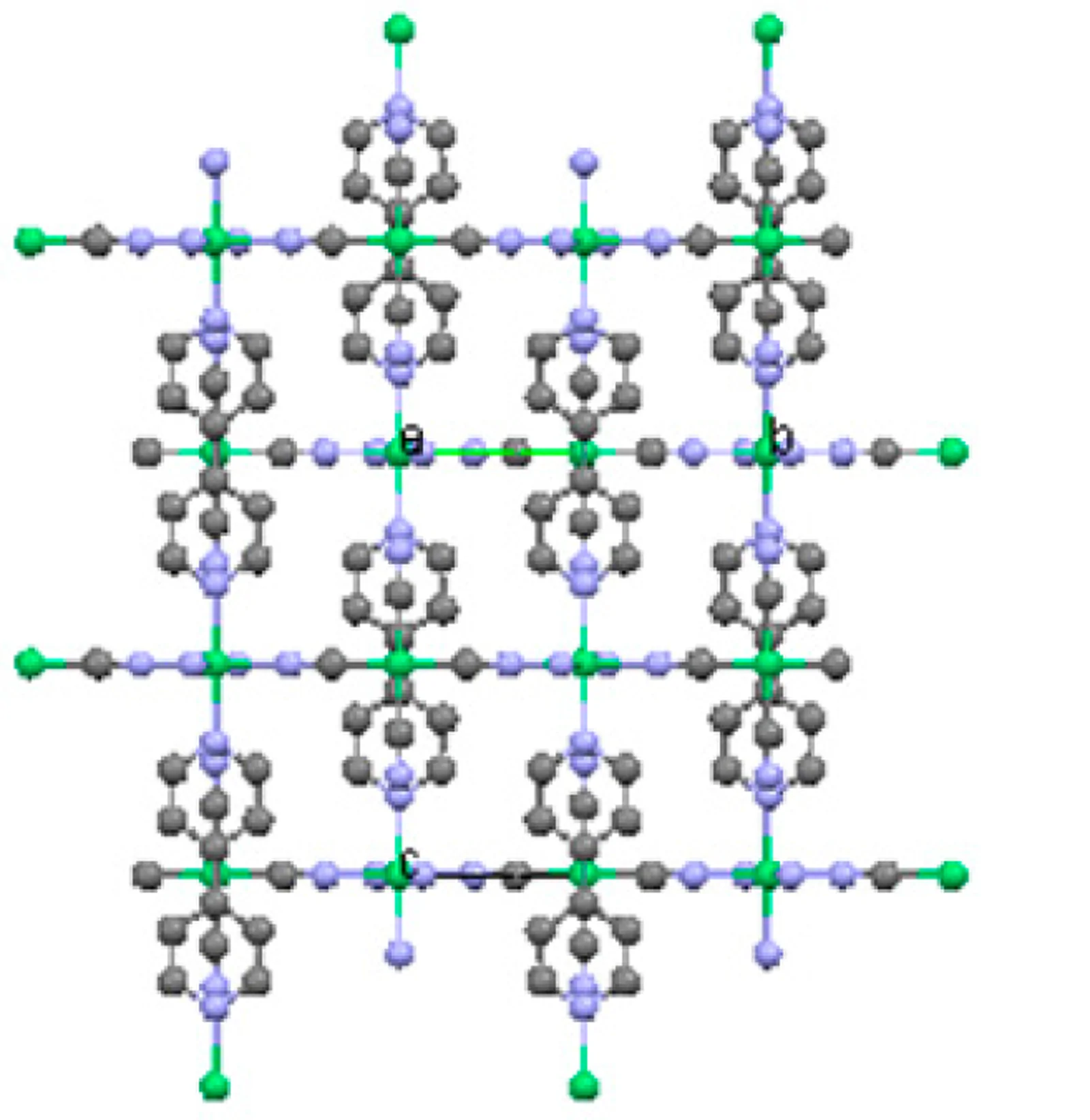
2-D structure of Ni-Bpy along *a*-axis, with sheet-like structure spreading in the *bc* direction (C-grey, N-blue, Ni-green).

**Figure 18.**
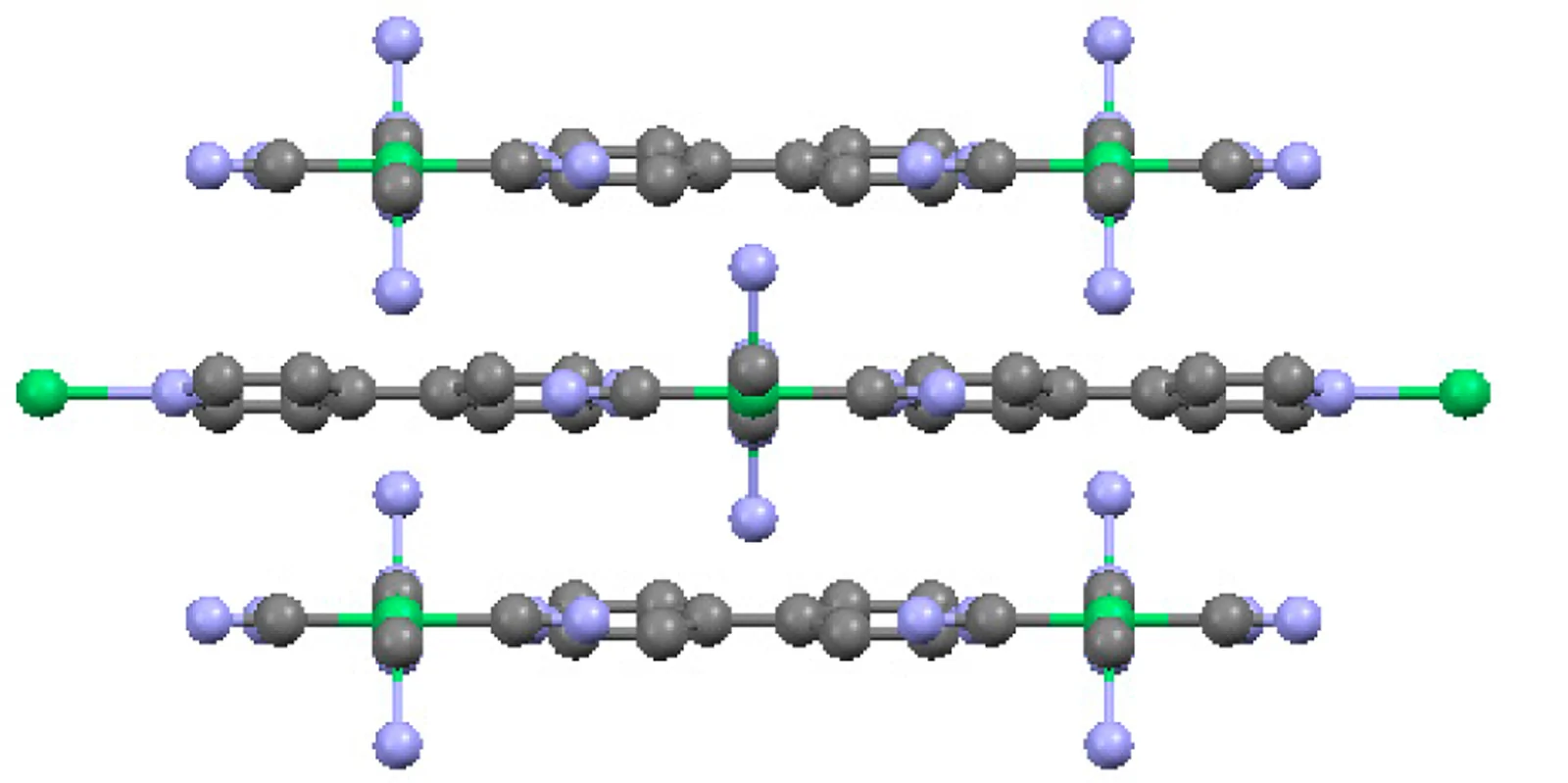
2-D sheets in Ni-Bpy are connected to each other via van der Waal’s forces and hydrogen bonding (view along *b*-axis; C-grey, N-blue, Ni-green).

**Figure 19.**
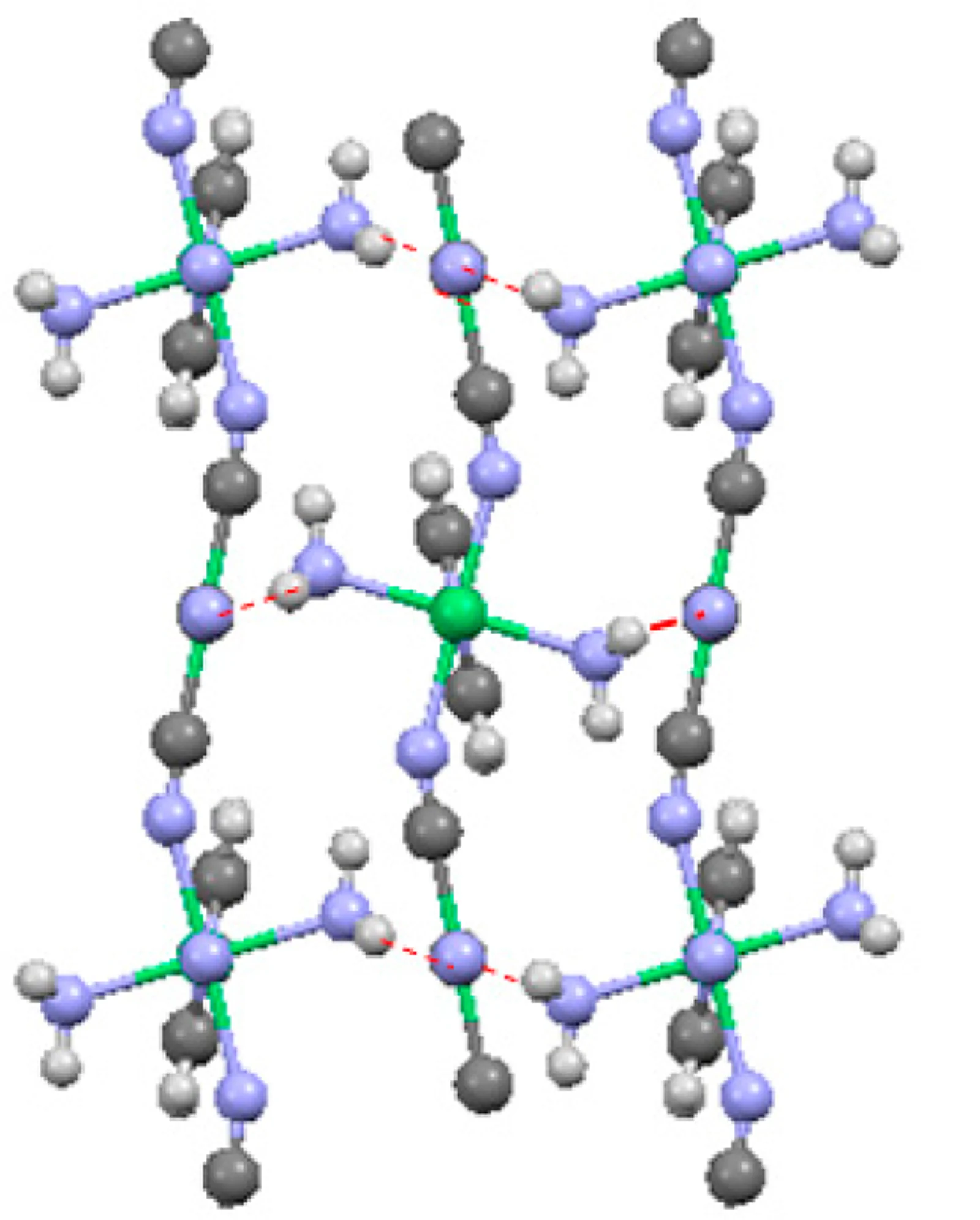
Terminal C≡N groups of the neighboring Ni-C-N-Ni-N-C-Ni chains in the Ni-Bpy structure forming a zigzag fashion (view along *c*-axis; C-grey, N-blue, Ni-green). The red broken lines indicate H-bonding.

The salient feature of the structure is that it consists of zigzag –Ni-NC-Ni-CN-Ni– chains. Each chain is further connected to the neighboring chains via the Bpy ligand to form a layered structure. The two pyridine rings are coplanar with each other. However, there is an abscence of a 3-D bridging of the layer to other layers via coordination bonds at the Ni sites. In other words, Ni-Bpy has a sheet-like structure spreading in the *bc* direction. Figure 17 gives the view of one such layer. This is because the two remaining available Ni^II^ sites on the other chain have been occupied with two strong Lewis bases, namely, the NH_3_ groups; therefore, the N site of the relatively weaker Lewis base –C≡N was “forced” to remain as a terminal group. This situation is different from a typical Hoffman-type of compound where instead of having the terminal –C≡N groups, the –C≡N groups bridge to the other Ni site on different chains to form a 2-D net (Figure 17). These sheets are connected to each other via van der Waal’s forces and hydrogen bonds (van der Waal radii for H is 1.2 Å, N: 1.55 Å [89]) (Figure 18). From Table S16, two of the Hs in the NH_3_ groups form hydrogen bonds (2.323 Å) to the terminal C≡N groups of the neighboring Ni-C-N-Ni-N-C-Ni chains in the structure as shown on Figure 19.

NH_3_, regarded as a basic molecule because it possesses a lone pair of electrons in its 3a1 orbital, is a classic example of an electron-donor Lewis base molecule [90] (a Lewis base donates a lone pair of electrons to a Lewis acid to form a Lewis adduct). This lone pair at the nitrogen is available for donation to other species (accept a proton) during bonding [90,91]. Madey and Houston [92] found that when NH_3_ is adsorbed on metal Ni(111), it is molecularly adsorbed, and is bonded to the surface via the N atom (becomes part of the structure) with the H atoms oriented away from the surface. Netzer and Madley [93] further confirmed that traces of pre-adsorbed oxygen on a metal surface will induce a high degree of azimuthal order in adsorbed molecules.

As a summary, one of the reasons that the 2-D nickel cyanide net does not form in Ni-Bpy is because of the strong Lewis acid–base reactions between NH_3_ with Ni that prevent the polymerization of the chains to become a 2-D net. Comparing to a number of other Hoffman-type compounds with Ni-groups, to our knowledge, this is one of the first compounds that we found in the Ni(Bpy)[Ni(CN)_4_] family that has NH_3_ groups as terminating ligands. Water of crystallization was reported to coordinate to the transition metals Ni or Cu [94–96]. Since our solvent of crystallization is a mixture of DMSO/H_2_O/NH_3_, apparently NH_3_ is a stronger Lewis base than H_2_O for competing for the coordination site to Ni.

It is unclear at this point why the functionalized bipyridine ligands BpyMe and BpyNH2 both give 3-D structures whereas the non-functionalized Bpy ligand yields a 2-D structure. One possibility is that the –R group in Ni-Bpy-R gives rise to steric hindrance which prevents the coordination of the NH_3_ groups to Ni, allowing the polymerization process to yield the 3-D structure. There may also be some effect where the higher symmetry of the Bpy ligand allows the 2-D network to propagate more quickly forcing the precipitation of this network before the loss of ammonia is complete. In the reaction with the less symmetrical Bpy-R, the kinetics for the propagation of the 2-D Bpy-R bridged structure may be slowed enough that this phase remains in solution longer. This would provide enough time for the NH_3_ concentration to drop to a point where the 3-D network can form via displacement of the coordinated NH_3_ ligands with bridging ligands. This mechanism is further supported by the formation of even lower dimensional 1-D chain structures with the shortest ligand in the series 2,6-naphthyridine. With the Naph linker (discussed below), the chain propagation kinetics are fast enough to crystallize the 1-D structure while the four blocking NH_3_ ligands remain coordinated to the octahedral Ni site.

### 2.3. One-Dimensional (1-D) Structure

#### (Ni(2,6-naphhyridine)[Ni(CN)_4_]), (Ni-Naph)

**Figure 20.**
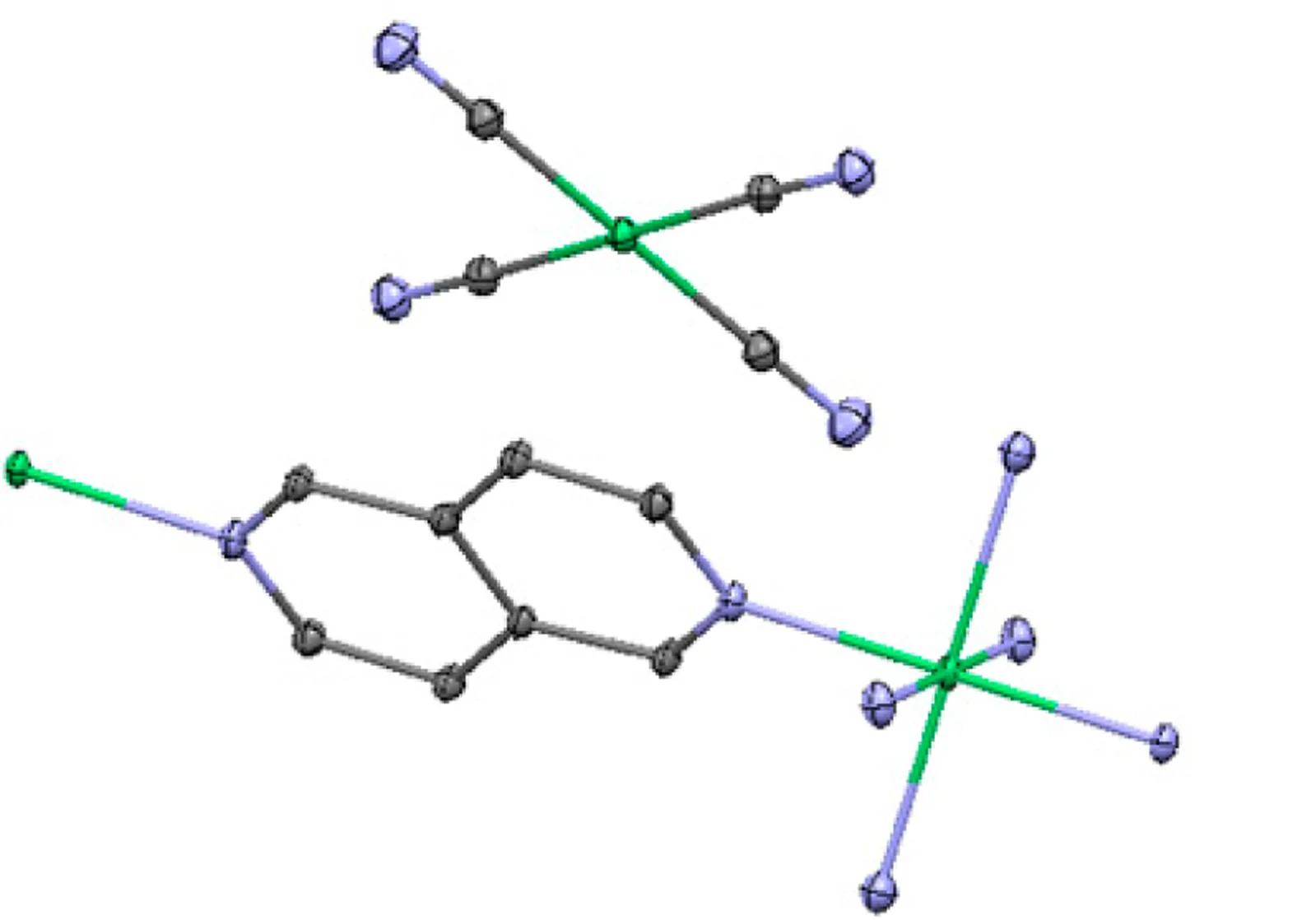
The basic motif of the Ni(Naph)[Ni(CN)_4_] molecules (C-grey, N-blue, Ni-green; probability ellipsoids drawn at 50%).

Tables S17–S21 give the atomic coordinates, anisotropic displacement parameters, selected pertinent bond distances, atomic coordinates for the hydrogen atoms and selected bond angles, respectively, for Ni-Naph. Figure 20 gives the basic motif of the Ni(Naph)[Ni(CN)_4_] molecules with partial labeling. A complete labeling of the crystallographic independent unit is given in supplementary Figure S5.

In the Ni-Naph compound, the ligand Naph has a relatively short length which consists of only two six-membered rings sharing one common edge, indicating if a 3-D cage-like structure forms; the resulting volume for CO_2_ capture is expected to be smaller than that in the Bpene, BpyMe and BpyNH2 compounds. The structure of Ni-Naph was found to be triclinic (space group *P-1*), with a relatively small unit cell dimension: *a* = 7.0315(3)Å, *b* = 7.9219(3)Å, *c* = 8.6103(3) Å, *α* = 67.816(1)°, *β* = 72.828(1)°, and *γ* = 73.698(1)°.

**Figure 21.**
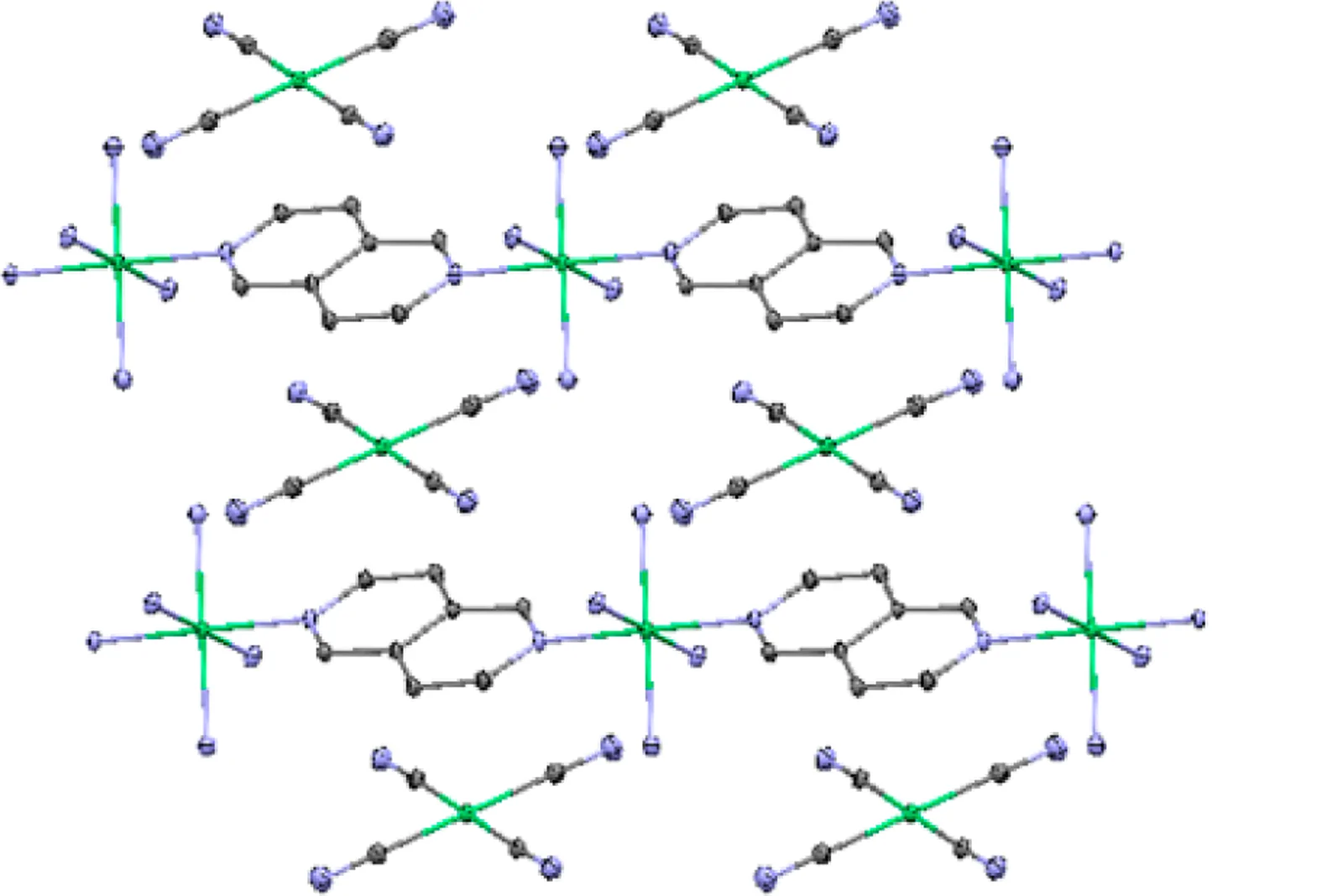
The Ni-Naph structure showing 1-D chains of Ni(Naph)(NH_3_)_4_ and individual units of planar [Ni(CN)_4_] groups (view along *b*-axis; C-grey, N-blue, Ni-green).

The salient feature of the Ni-Naph compound is that instead of having a 3-D or 2-D structure, it consists of one-dimensional chains running parallel to the *c*-axis (Figure 21). The Naph ligand, while coordinating to Ni of the Ni(CN)_4_ groups to form chains, it prevents the formation of a 2-D tetracyanonickelate net. In the Ni-Naph structure, the one-dimensional chains alternate with discrete Ni(CN)_4_ groups. Four NH_3_ groups instead of the expected C≡N groups were found bonding to the six-fold coordinated Ni site. These NH_3_ groups are terminated groups instead of propagating to form a higher dimension structure. As a result, 1-D chains of Ni(Naph)(NH_3_)_4_ and discrete units of planar [Ni(CN)_4_] groups were found parallel to each other along *c*-axis. The chemical formula of this compound (Ni_2_C_12_N_10_H_18_), can be written in terms of chemical moiety as Ni(CN)_4_·Ni(NH_3_)_4_·C_8_N_2_H_6_.

In the 1-D Ni(Naph)(NH_3_)_4_ chains, the N1-Ni1-N1 and N3-Ni1-N3 angles are all 180°. The six-fold coordinated Ni-N distances (2.1429(8) Å, 2.1145(8) Å, and 2.1353(9) Å) are within the expected Ni-N distances. In the discrete Ni(CN)_4_ units, the Ni2-C6 and Ni2-C5 bond distances of 1.8627(10) Å and 1.8588(10) Å are shorter than those of the Ni-N distances, as expected. The C5-Ni2-C5 angles and the C6-Ni2-C6 angles are all 180°. The –C≡N distances (1.1565(14) Å and 1.1592(14) Å) are within the expected range.

Another 1-D square tetracyano complex was also reported earlier by Černák and Abboud [97]. They found a one-dimensional structure of catena-poly[Ni_2_(CN)_4_(C_10_H_8_N_2_)_2_]_n_, consisting of infinite zigzag chains running parallel to the c-axis of the structure. The chains are composed of paramagnetic [Ni(dipy)_2_]^2+^ cations linked by diamagnetic [Ni(CN)_4_]^2−^ anions via bridging cyano groups. The bridging cyano group occupies a *cis* position in the cation and a *trans* position in the anion, resulting in a cis-trans-type square tetracyano complexes.

All these molecules are connected to each other via intermolecular covalent forces. This is another excellent example illustrating that the concentration of solvent mixture of crystallization has an important effect on the final product of crystal growth and their structures. Different strategies such as varying the outgas rate of NH_3_ will be pursued for obtaining the 3-D structure of Ni-Naph.

## 3. Experimental Section

Certain trade names and company products are mentioned in the text or identified in illustrations in order to adequately specify the experimental procedures and equipment used. In no case does such identification imply recommendation or endorsement by the National Institute of Standards and Technology.

The details of the powder syntheses of these MOF compounds using a different synthetic method are reported elsewhere [61].

### 3.1. Material Synthesis and Crystal Growth

A technique [84] that we have found to be versatile for crystallization of Ni(L)[Ni(CN)_4_] compounds is a modification of a procedure originally used by Černák et al. to prepare crystalline 1-D [Ni(CN) _4_] containing chain compounds [97]. The approach involves the use of NH_3_ as a blocking ligand since a sufficient concentration of NH_3_ will prevent the formation of Ni-CN-Ni and Ni-L-Ni bridges which are required for polymerization. If the reaction mixture is contained in an open flask, the NH_3_ will outgas from the solution. Once the concentration of NH_3_ drops below a threshold level, assembly of the Ni(L)[Ni(CN)_4_] material will commence. Using a H_2_O/DMSO mixture as the solvent and a reaction temperature of ≈90 °C provided the necessary combination of NH_3_ outgassing rate and oligomer solubility to produce the polymeric structures. A good crop of crystals can typically be obtained in 24 h to 72 h. The technique has been found to be adaptable to various organic bridging ligands (L) in our laboratory. The general synthetic strategy has been reported previously [84] and its adaptation to the synthesis of the materials in the current report is as follows. The reactants 3-methyl-4,4’-bipyridine; 3-amino-4,4’-bipyridine; nickel cyanide hydrate Ni[Ni(CN)_4_]_n_(H_2_O)_3_ were prepared as previously described [61]. The 2,6-naphthyridine was purchased from the Florida Center for Heterocyclic Compounds and used as received. Water was purified by an in-house reverse osmosis system. All other reagents were purchased from Sigma-Aldrich and used as received. Precise yields are not given since the crystals are stored in the mother liquor and only removed in random batches as needed for analysis and other characterizations. Estimated yields for all reactions are in the range of 50%–75%.

#### Ni-BpyMe.

At room temperature, a 50 mL flask was charged with 0.5 mmol polymeric nickel cyanide hydrate Ni[Ni(CN)_4_]_n_(H_2_O)_3_ and made to dissolve through the sequential addition of 6 mL H_2_O, 6 mL of reagent concentrated ammonium hydroxide solution, and 9 mL DMSO with stirring. (Caution: do not mix concentrated ammonium hydroxide and DMSO directly as rapid outgassing of NH_3_ will occur.) A solution of 0.5 mmol BpyMe in 9 mL warm DMSO was then added to the resulting solution. A six-inch air-cooled condenser was added and the flask was transferred to an oil bath preheated to 70 °C. The temperature of the oil bath was ramped to 90 °C over a time span of approximately 30 min after which time the stirring was stopped and the reaction left at 90 °C undisturbed for two days. The dissolved NH_3_ bubbled out of the solution rapidly during the initial hour, then slowed significantly while a crystalline product formed over the next two days. After completion, the reaction mixture was cooled to room temperature and the resulting crystals were isolated by first decanting off any suspended fine particles suspension of impurities while being careful to leave the crystals wetted within the reaction mixture. The impurities were removed by filtration and the filtrate returned to the flask. The pipetting and filtration cycle was repeated until the powdered impurities were removed. The light violet crystals were kept stored in the cleaned reaction mixture in a capped vial at room temperature until the X-ray diffraction measurements were taken.

#### Ni-BpyNH2.

By the same general procedure used for Ni-BpyMe, 0.60 mmol of BpyNH2 was reacted with 0.5 mmol Ni_2_(CN)_4_ hydrate in a mixture of 6 mL H_2_O, 6 mL concentrated aqueous NH_3_, and 18 mL DMSO. The product contains a significant amount of a green powder impurity that obstructed complete characterization of the crystalline product by gas adsorption. A TGA scan was taken on a few pure crystals harvested from the stir bar.

#### Ni-Bpy.

The crystals were prepared using the same general procedure and reactant ratios as for Ni-BpyMe, with the exception that Bpy was used in place of BpyMe.

#### Ni-Naph.

The crystals were prepared using the same general procedure and reactant ratios as for Ni-BpyMe, with the exception that 2,6-naphthyridine was used in place of BpyMe.

### 3.2. Synchrotron X-Ray Diffraction Experiment

As most crystals are twin or multiplets, the crystals that we used for data collection in general have very small dimensions (for example, the Bpene crystal has a dimension of 0.016 × 0.011 × 0.006 mm^3^ and it has a thin-plate morphology). The crystals were mounted on the tip of a glass fiber with paratone oil and cooled down to 100 K with an Oxford Cryojet. X-ray diffraction experiments were performed with a Bruker D8 diffractometer in the vertical mount with a APEX II CCD detector (Bruker Corporation, Madison, WI, USA) using double crystals technique with Si(111) monochromator at 30 keV (λ ranges from 0.41328 Å to 0.4428 Å for the current studies) at sector 15 (ChemMatCARS), Advanced Photon Sources (APS). Data were processed with APEX2 suite software [98] for cell refinement and reduction. The structure was solved by the direct method and refined on F^2^ (SHELXTL) [99]. Non-hydrogen atoms were refined with anisotropic displacement parameters, and hydrogen atoms on carbons were placed in idealized positions (C-H = 0.95 Å).

### 3.3. Sorption Experiments

Gravimetric gas adsorption measurements for the Ni-BpyMe sample were conducted on a Hiden IGA microbalance (Hiden Isochema, Warrington, United Kingdom). The crystalline sample was first extracted by soaking in methanol at room temperature overnight. The methanol-exchanged sample (≈25 mg) was activated by heating under vacuum at 70 °C until the sample weight stabilized. Isotherms were then measured under flowing gas regulated by a mass flow controller and back pressure regulator. Equilibrium was determined at each pressure step using an internal fitting algorithm in the instrument control software. Buoyancy corrections were then applied to the final equilibrium weights using known densities of all components in the sample and counterweight chambers from gas densities calculated using REFPROP software [100].

Sample integrity of the Ni-BpyMe sample after solvent exchange with methanol and evacuation was verified by comparing the powder X-ray diffraction pattern of the activated sample to one calculated from the solvated Ni-BpyMe crystal structure using Mercury software [101]. The pattern was also compared to one from a previously reported synthesis of the polycrystalline Ni-BpyMe sample [61]. Taking into consideration that slight variations in the evacuated and solvated patterns are expected due to changes in symmetry upon guest removal, the two patterns give satisfactory agreement. The powder pattern reported for the evacuated polycrystalline material in [61] also agrees well with the current sample. The powder diffraction results are included as supporting information (Figure S6).

### 3.4. Thermogravimetric Analyses

**Figure 22.**
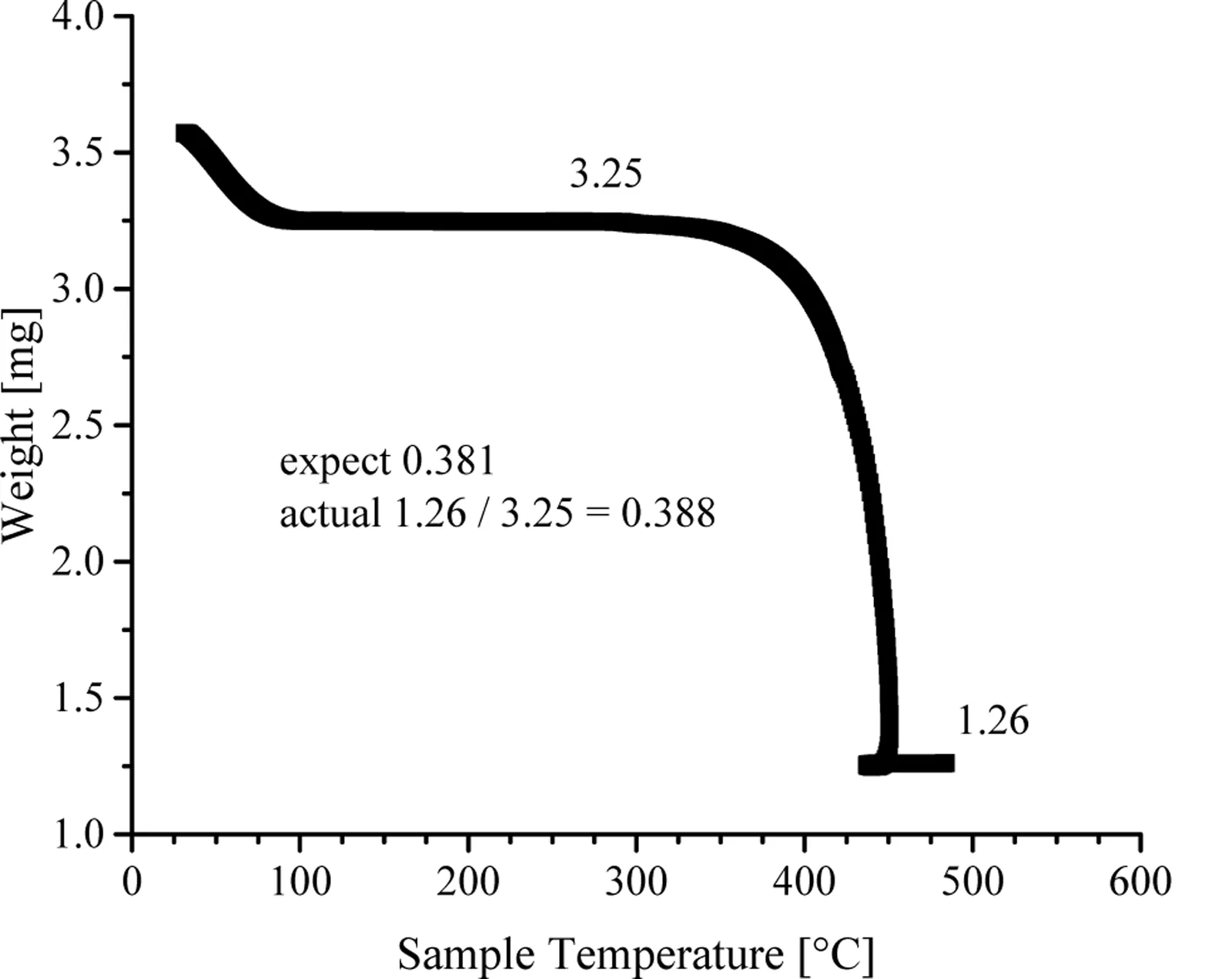
TGA scan in air for crystals of Ni-BpyMe after extracting DMSO guests with methanol. All methanol guests are lost below 100 °C with no further weight loss prior to decomposition. The “expected” value on the plot is the expected ratio of the mass of residual NiO to mass of the guest-free framework as determined from the structure determined by single-crystal X-ray diffraction, whereas the “actual” ratio is the experimentally determined value taken at the indicated points in the scan. See Experimental Section for an explanation of how the ratio is calculated.

Thermogravimetric analyses were performed using a Mettler STARe TGA/DSC thermogravimetric analyzer (Mettler Toledo, Columbus, Ohio, USA). Samples (typically 5 mg to 10 mg) were run in a Pt pan under a dry air purge at temperatures >550 °C. Sample purity was determined from the ratio of the residual weight of NiO in the sample pan to the guest-free weight of the material at the appropriate place in the TGA scan. This experimental ratio was then compared to the NiO ratio expected from the guest-free formula weight of the material as determined from the crystal structure. A TGA scan in air will then allow the guest-free framework mass to be determined, which upon combustion in air will yield NiO. Since only one composition of NiO is possible, the method works well for these compounds, as opposed to other materials which contain variable oxidation state metals such as iron, cobalt, copper, manganese, etc. which could form mixed oxidation state oxides during the combustion. With nickel compounds, the TGA technique works according to the simple decomposition reaction: guest-free framework Ni(L)Ni(CN)_4_ → 2NiO. As such, the ratio of the mass of residual NiO to the mass of the guest-free framework in the TGA scan is the same as the ratio of the formula weight of NiO to the formula weight of the guest-free framework. Using the data in Figure 22 for the Ni(Bpy-Me)Ni(CN)_4_ compound as an example: Expected formula weight of Ni(Bpy-Me)Ni(CN)_4_ is 391.67 g/mol and the formula weight of 2NiO is 149.39 g/mol. The ratio of the formula weight of NiO to Ni(Bpy-Me)Ni(CN)_4_ = 149.39/391.67 = 0.3814. This same ratio should be observed in TGA scan when using the residual mass of NiO to the dry framework mass. From the TGA data, the residual mass of NiO is 1.26 mg and the mass of the guest-free framework is 3.25 mg. The ratio is 1.26/3.25 = 0.3877. This is within 98% of the expected value, thus confirming the crystal composition. The actual and expected values for the NiO to framework ratios for the other samples gave similar agreements. The values for each sample are shown on the TGA plots in the respective figures in the manuscript.

**Figure 23.**
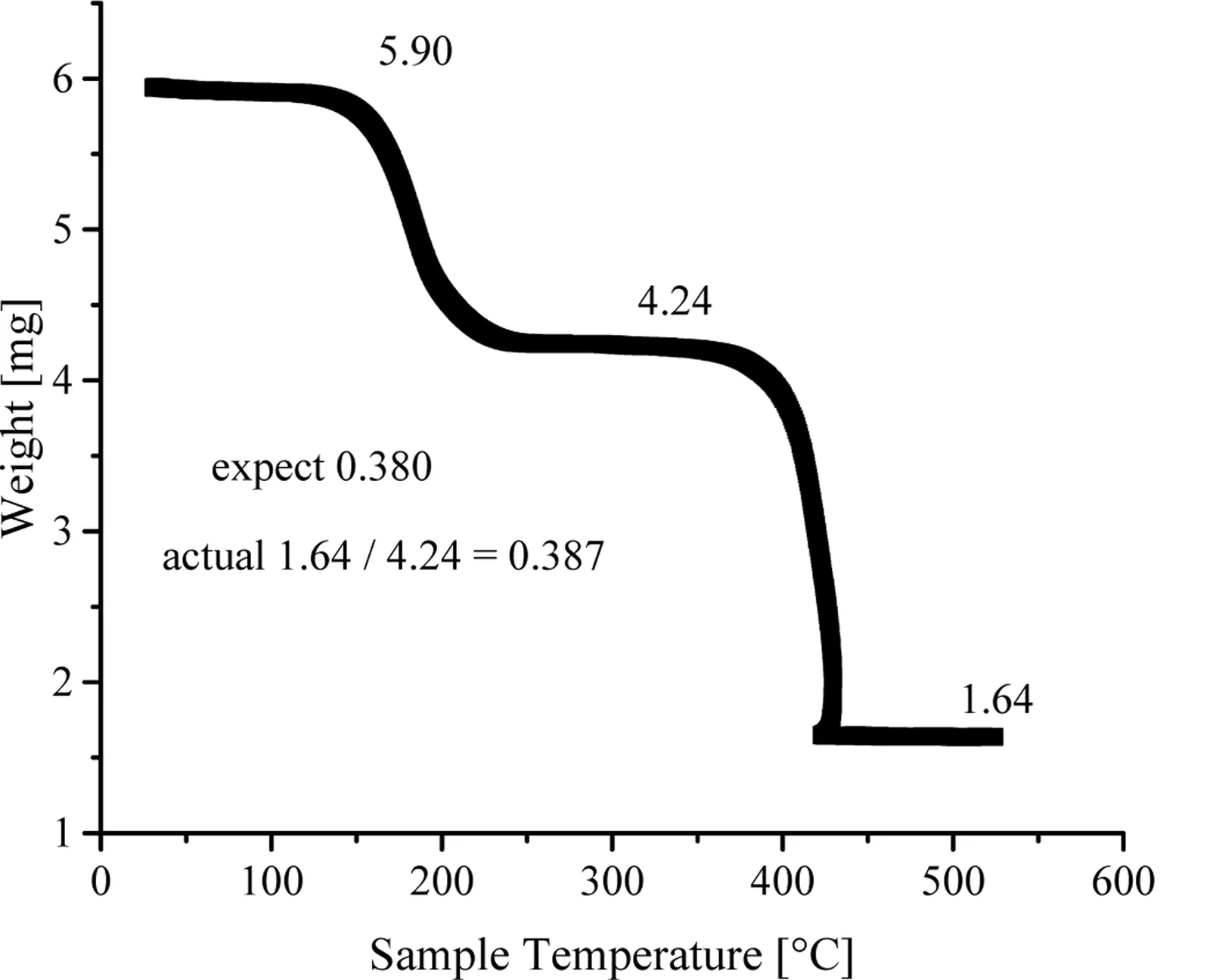
TGA scan in air for neat clean crystals of Ni-BpyNH2. Loss of guest DMSO is complete by 250 °C yielding the guest-free framework. The “expected” value on the plot is the expected ratio of the mass of residual NiO to the mass of the guest-free framework as determined from the structure determined by single-crystal X-ray diffraction, whereas the “actual” ratio is the experimentally determined value taken at the indicated points in the scan. See Experimental Section for an explanation of how the ratio is calculated.

**Figure 24.**
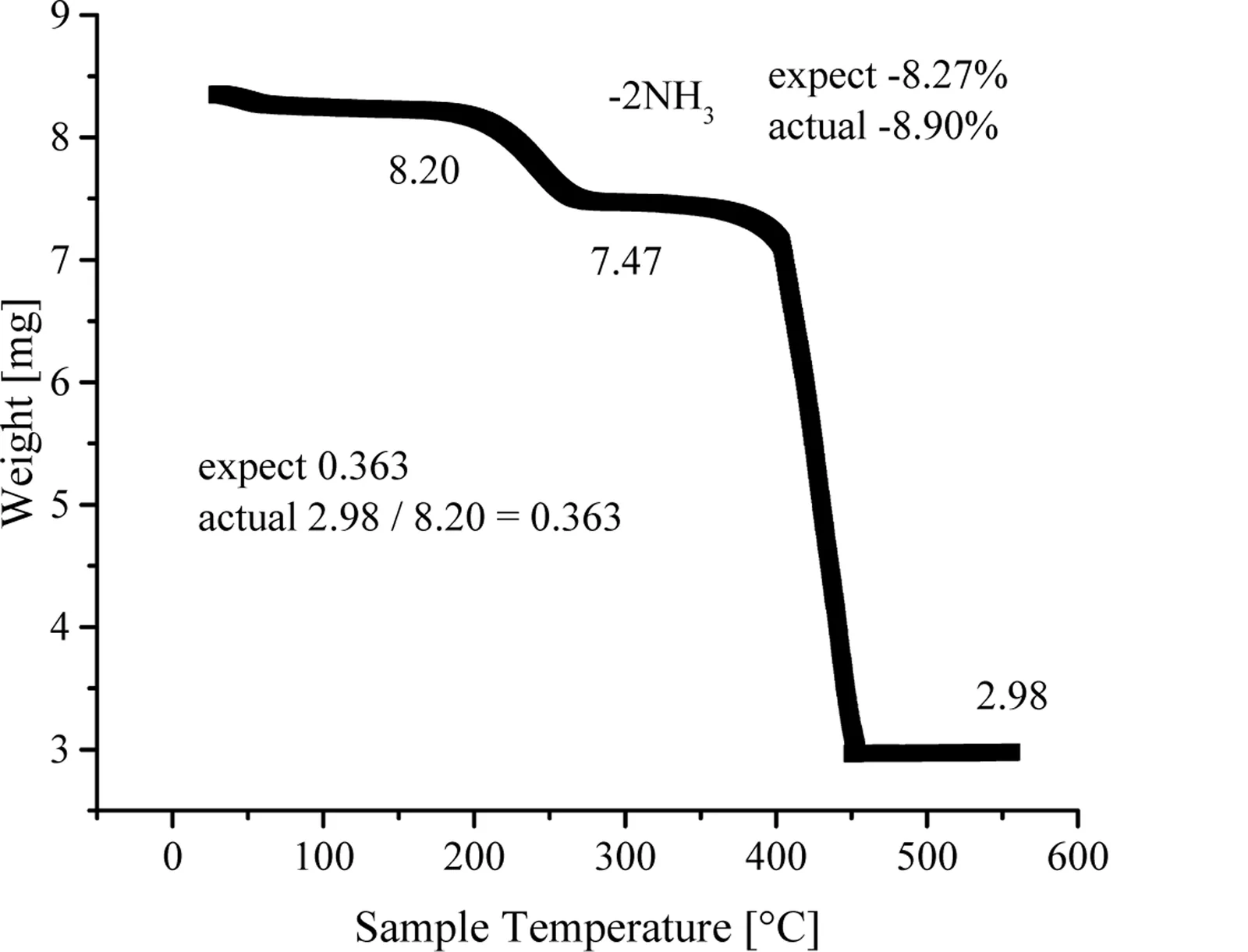
TGA scan in air for neat crystals of Ni-Bpy. Loss of coordinated NH_3_ occurs over the temperature range of 200 °C to 300 °C. The “expected” value on the plot is the expected ratio of the mass of residual NiO to the mass of the framework as determined from the structure determined by single-crystal X-ray diffraction, whereas the “actual” ratio is the experimentally determined value taken at the indicated points in the scan. See Experimental Section for an explanation of how the ratio is calculated.

**Figure 25.**
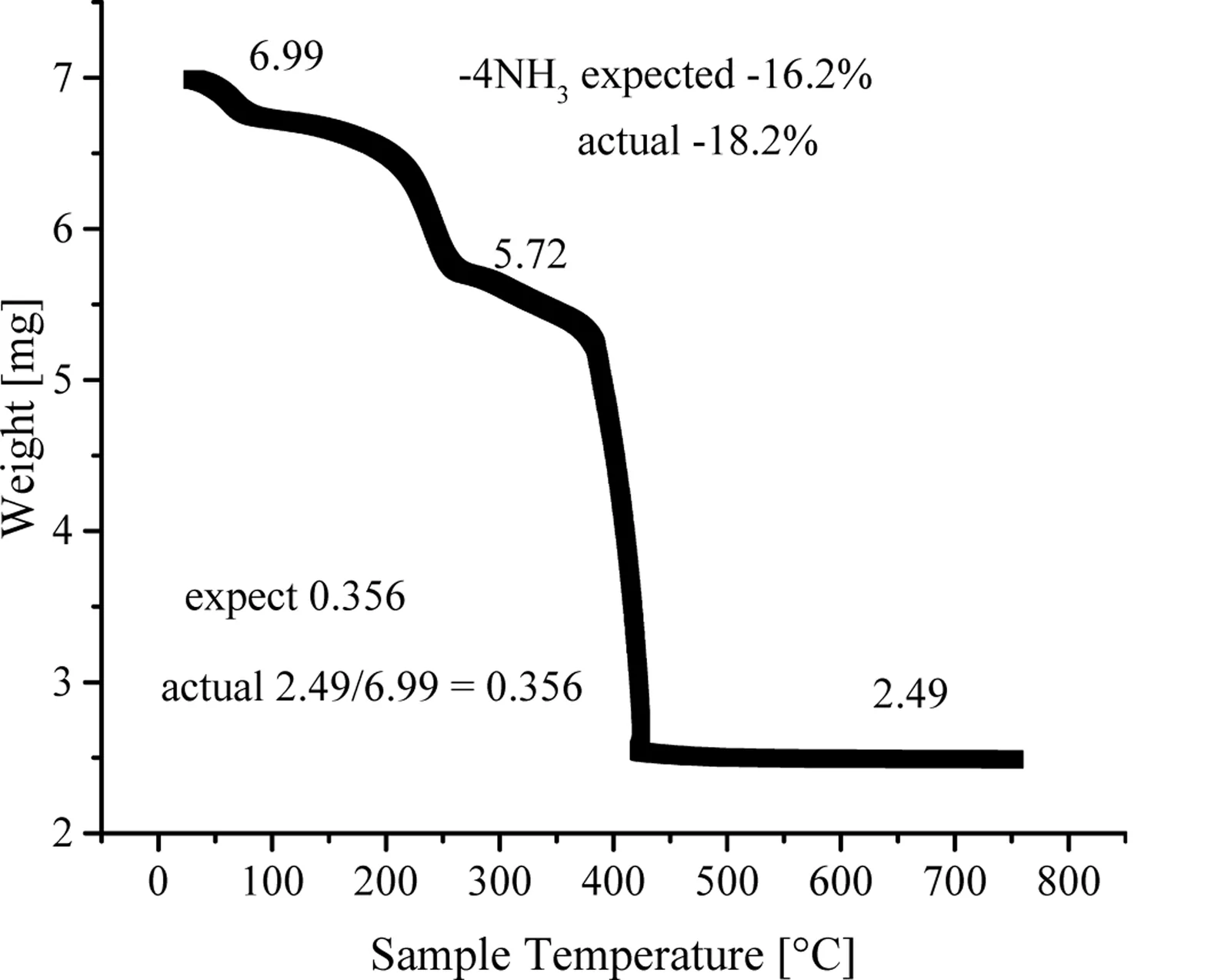
TGA scan in air for neat crystals of Ni-Naph. Loss of NH_3_ occurs in two steps out to 300 °C followed by immediate decomposition of the material. (The initial small weight loss at low temperature may also indicate some impurity in the bulk sample as discussed in the main text.) The “expected” value on the plot is the expected ratio of the mass of residual NiO to the mass of the framework as determined from the structure determined by single-crystal X-ray diffraction, whereas the “actual” ratio is the experimentally determined value taken at the indicated points in the scan. See Experimental Section for an explanation of how the ratio is calculated.

A TGA scan in air for a sample of neat crystals of Ni-BpyNH2 harvested from the stir bar is shown in Figure 23. The ratio of residual NiO to the guest-free framework (plateau region 250–300 °C after loss of guest DMSO) agrees well with that expected based on the guest-free Ni-BpyNH2 formula weight of 392.65 g/mol determined from the crystal structure. The TGA scan in air for crystals of neat Ni-Bpy is shown in Figure 24. The ratio of residual NiO to the crystal framework (plateau region 100–150 °C after loss of surface water) agrees well with that expected based on the Ni-Bpy formula weight of 411.70 g/mol determined from the crystal structure. The loss of the two coordinated NH_3_ ligands per Ni(Bpy)(NH_3_)_2_[Ni(CN)_4_] unit are shown by the step at ~200 °C. The relative weight loss for the release of the coordinated ammonia also agrees well with that expected from the crystal structure. The TGA scan in air for crystals of neat Ni-Naph are shown in Figure 25. The ratio of residual NiO to the crystal framework agrees well with that expected based on the Ni-Naph formula weight of 419.73 g/mol determined from the crystal structure if the initial starting mass in the TGA scan is used; however, the results are not as clear as with the other three samples. The sample does show a small initial mass loss below 100 °C which transitions into a larger step indicating that the loss of coordinated NH_3_ ligands is complete by ≈250°C. The low-temperature weight loss may indicate that the bulk sample has some small fraction of material in which coordinated NH_3_ ligands are partially replaced with coordinated water ligands or that the bulk material is not 100% phase pure. A likely impurity in the bulk material would be the result of incomplete removal of an insoluble green powder phase that precipitates with the crystalline sample, similar to that noted for the NiBpy-NH2 sample above.

## 4. Conclusions

Structure data for the PICNIC series of compounds increases our understanding of coordination chemistry and interactions of guest-host phenomena in these versatile materials. In particular, it’s interesting to compare the structures and guest adsorption behaviors of the 3-D structures when the ligands are Bpene, BpyMe and BpyNH2. All three materials adopt a similar pillared layer motif; however, the Bpene compound shows structurally dynamic behavior which transitions between a low-porosity and high-porosity state during the adsorption and desorption of guests, such as CO_2_, whereas the BpyMe and BpyNH2 compounds retain a rigid structure with no variation in pore structure between the guest-loaded and guest-free states. One possible explanation for the variation in structural rigidity in the series of materials may lie in the Ni-N-C bond angles associated with the octahedral Ni sites in the structures. These bonds result from the bridging interaction of the cyanide ligand between the square planar Ni_A_(CN)_4_ complexes and the octahedral Ni_B_ sites. The Ni-Bpene material shows the greatest deviation from linearity with both Ni2-N3-C3 and Ni1-N4-C4 having bond angles of 171.4°. This bridging interaction corresponds to the Ni-N-C-Ni linkage running along the *bc* face diagonal. In contrast, the highest deflection in the Ni-BpyMe compound occurs in Ni1-N2-C8 with an angle of 174.3°. In Ni-BpyNH2, the bond is even more linear with a bond angle between Ni2-N1-C1 of 176.4°. (See supporting information Figures S1–S3) From this observation, there appears to be a stronger driving force to form a more corrugated Ni[Ni(CN)_4_]_n_ network in the Bpene material which results in a more significant tilt in the Bpene pillar ligands relative to the Ni[Ni(CN)_4_]_n_ plane. A flexible, hinge-like bond corresponding to the Ni-N-C linkage would provide a means for the relative tilt of the pillars to change, which in turn, would directly affect the inter-pillar distance and available pore volume.

The relative rates of NH_3_ outgassing and Ni-(L)-Ni chain propagation are important for the final structures of the single crystals. When the ligands were Bpene, BpyMe and BpyNH2, the relative rates are favorable for the formation of extended 3-D structures. In these materials, the pillar ligands and the Ni[Ni(CN)_4_] layers encase empty voids of parallelepiped shapes which are created by the absence of ligand coordination to the two Ni sites of the square plane [Ni(CN)_4_] building units. These void spaces provide pockets for the encapsulation of guest molecules. When these reaction rates vary significantly, structures with a lower dimensionality are obtained including a 2-D layered structure when Bpy is a bridging ligand and a 1-D structure when the bridging ligand is Naph.

In the 2-D and 1-D structures, the octahedral Ni sites contain coordinated –NH_3_ ligands which effectively block chain propagation in all three dimensions. In the 2-D structure, the two axial sites of the octahedral Ni complex are blocked by –NH_3_, allowing chain propagation through Bpy and Ni(CN)_4_ bridging interactions to occur in the *xy* plane only. Furthermore, when the ligand is Naph, a 1-D structure resulted. In the 1-D chain, the six-fold coordinated Ni is surrounded by four –NH_3_ groups while the chain is propagated along the –Naph-Ni-Naph-Ni– direction (*c*-direction). The chains are further interleaved with discrete isolated Ni(CN)_4_ units. Future challenges will be to refine the synthesis process in order to convert these low-dimensional structures into 3-D ones.

Results of more single-crystal data with a greater variety of ligands with different lengths and functional groups will further enhance our understanding of these materials. Continuing research is ongoing in our laboratories to prepare and characterize other novel 3-D PICNICs, and to explore their adsorption properties.

## Supplementary Material

Supp1

**Supplementary Materials:** The supplementary materials are available online at http://www.mdpi.com/2073–4352/6/9/108/s1.

## Figures and Tables

**Table 1.** Crystal Data and Structure Refinement for Four Cyanonickelate Compounds.

	Ni-BPyMe	Ni-BPyNH2	Ni-BPy	Ni-Naph

Formula (Expected)	C_15_H_10_N_6_Ni_2_	C_14_H_9_N_9_Ni_2_	C_14_H_8_N_6_Ni_2_	C_12_H_6_N_6_Ni_2_
Formula (Determined)	C_19_H_22_N_6_Ni_2_O_2_S_2_	C_18_H_21_N_7_Ni_2_O_2_S_2_	C_7_H_7_N_4_Ni	C_12_H_18_N_10_Ni_2_
Formula weight	546.96	543.92	205.88	419.78
Temperature (K)	100(2)	100(2)	100(2)	100(2)
Wavelength (Å)	0.41328	0.4428	0.41328	0.41328
Crystal system	Monoclinic	Orthorhombic	Orthorhombic	Triclinic
Space group	*P2/n*	*Cmca*	*Pnnm*	*P-1*
Unit cell dimension				
*a* (Å)	13.3483(14)	14.7000(5)	7.2641(9)	7.0315(3)
*b* (Å)	7.1002(7)	22.6879(7)	9.9285(12)	7.9219(3)
*c* (Å)	13.5625(14)	13.8028(4)	11.4008(14)	8.6103(3)
*α* (°)				67.8160(10)
*β* (°)	114.834(2)			72.2820(10)
*γ* (°)				73.6980(10)
*V* (Å^3^)	1166.5(2)	4603.4(2)	822.2(2)	415.51(3)
*Z*	2	8	4	1
D_cal_, mg/m^3^	1.557	1.570	1.663	1.678
μ (mm^−1^)	0.412	0.462	0.475	0.517
F(000)	562	2216	420	216
θ range (°)	1.044 to 14.349	1.379 to 23.456	1.581 to 14.351	1.524 to 23.837
Index ranges	−16 ≤ h ≤ 1	−24 ≤ h ≤ 20	−8 ≤ h ≤ 8	−13 ≤ h ≤ 13
	−8 ≤ k ≤ 8	−40 ≤ k ≤ 39	−11 ≤ k ≤ 9	−15 ≤ k ≤ 14
	−16 ≤ l ≤ 16	−17 ≤ l ≤ 22	−13 ≤ l ≤ 12	−10 ≤ l ≤ 15
No. reflections	14101	33326	6410	12740
R(intensity)	0.0866	0.0862	0.112	0.0435
Completeness, θ(%)	14.357(99.9)	15.407(97.1)	14.357(94.3)	14.357(96.9)
Refinement method	Full matrix LSQ on F^2^	Full matrix LSQ on F^2^	Full matrix LSQ on F^2^	Full matrix LSQ on F^2^
Data/restraints/parameters	2116/6/169	5839/48/159	740/0/81	5162/0/148
Goodness-of-fit on F^2^	1.152	1.054	1.086	1.030
R indices [I>2sigma(I)]				
R1	0.0586	0.0923	0.0393	0.0300
wR2	0.1463	0.2582	0.1072	0.0750
R indices (all data)				
R1	0.0991	0.1587	0.0411	0.0424
wR2	0.1754	0.3022	0.1103	0.0801
Extinction coefficient			0.18(3)	
Largest diff. peak (e.Å^−3^)	0.902	2.952	1.008	0.751
Largest diff. hole (e.Å^−3^)	−0.523	−2.676	−0.65	−0.729

## References

[1] Etheridge DM; Steele LP; Langenfelds RL; Francey RJ; Barnola JM; Morgan VI Natural and anthropogenic changes in atmospheric CO_2_ over the last 1000 years from air in Antarctic ice and firn. J. Geophys. Res. Atmos. 1996, 101, 4115–4128. [CrossRef]

[2] Gammon RH; Sundquist ET; Fraser PJ History of carbon dioxide in the atmosphere. Atmospheric Carbon Dioxide and the Global Carbon Cycle; DOE/ER-239; Trabalka JR, Ed.; U.S. Department of Energy: Washington, DC, USA, 1985; pp. 25–62.

[3] Espinal L; Poster DL; Wong-Ng W; Allen AJ; Green ML Standards, Data, and Metrology Needs for CO_2_ Capture Materials—A Critical Review. Environ. Sci. Technol. 2009, 47, 11960–11975. [CrossRef] [PubMed] 10.1021/es402622q 24060087

[4] Choi S; Drese J; Jones CW Absorbent Materials for carbon Dioxide Capture from Large Anthropogenic Point Source. ChemSusChem 2009, 2, 796–854. [CrossRef] 19731282 10.1002/cssc.200900036

[5] Wang B; Cote AP; Furukawa H; O’Keeffe MJ; Yaghi OM Colossal Cages in Zeolite Imidazolate Frameworks as Selective Carbon Dioxide Reservoirs. Nature 2008, 453, 207. [CrossRef] [PubMed] 18464739 10.1038/nature06900

[6] Wong-Ng W; Kaduk JA; Huang Q; Espinal L; Li L; Burress JW Investigation of NaY Zeolite with Adsorbed CO_2_ by Neutron Powder Diffraction. Microporous Mesoporous Mater. 2013, 172, 95–104. [CrossRef]

[7] Siriwardane RV; Shen M-S; Fisher EP; Poston JA Adsorption of CO2 on Molecular Sieves and Activated Carbon. Energy Fules 2001, 15, 279–284. [CrossRef]

[8] Fripiat JJ; Cruz MM; Bohor BF; Thomas J Jr. Interlamellar Adsorption of Carbon Dioxide by Smectites. Clays Clay Miner 1974, 22, 23–30. [CrossRef]

[9] Sayyah I; Lu Y; Mall RI; Suslick KS Mechancial Activation of CaO-Based Adsorbates for CO2 Capture. ChemSusChem 2013, 6, 193–198. [CrossRef] [PubMed] 23132751 10.1002/cssc.201200454

[10] Ochoa-Fernández E; Rønning M; Grande T; Chen D Synthesis and CO_2_ Capture Properties of Nanocrystalline Lithium Zirconate. Chem. Mater. 2006, 18, 6037–6046. [CrossRef]

[11] Hutson ND Structural Effects on the High Temperature Adsorption of CO_2_ on a Synthetic Hydrotalcite. Chem. Mater. 2004, 16, 4135–4143. [CrossRef]

[12] Zhou HC; Kitagawa S Metal–Organic Frameworks (MOFs). Chem. Soc. Rev. 2014, 43, 5415–5418. [CrossRef] [PubMed] 25011480 10.1039/c4cs90059f

[13] Dey C; Kundu T; Biswal BP; Mallick A; Banerjee R Crystalline metal-organic frameworks (MOFs): Synthesis, structure and function. Acta Cryst. 2014, B70, 3–10. [CrossRef] [PubMed] 10.1107/S2052520613029557 24441122

[14] Woerner WR; Plonka AM; Chen X; Banerjee D; Thallapally PK; Parise JB Simultaneous in situ X-ray Diffraction and Calorimetric Studies as a Tool to Evaluate Gas Adsorption in Microporous Materials. J. Phys. Chem. 2016, 120, 360–369.

[15] Liu Y; Wang ZU; Zhou H-C Recent Advances in Carbon Dioxide Capture with Metal-Organic Frameworks. Greenh. Gas Sci. Technol. 2012, 2, 239–259. [CrossRef]

[16] Yaghi OM; Li Q Reticular Chemistry and Metal-Organic Frameworks for Clean Energy. MRS Bull. 2009, 34, 682–690. [CrossRef]

[17] Morris RE; Wheatley PS Gas Storage in Nanoporous Materials. Angew. Chem. Int. Ed. 2008, 47, 4966–4981. [CrossRef] [PubMed] 10.1002/anie.200703934 18459091

[18] Rowsell JLC; Yaghi OM Metal-Organic Frameworks: A New Class of Porous Materials. Microporous Mesoporous Mater. 2004, 73, 3–14. [CrossRef]

[19] Chen R; Yao J; Gu Q; Smeets S; Barlocher C; Gu H; Zhu D; Morris W; Yaghi OM; Wang HA Two-Dimensional Zeolitic Imidazolate Framework with a Cushion-Shaped Cavity for CO_2_ Adsorption. Chem. Commun. 2013, 49, 9500–9502. [CrossRef] [PubMed] 10.1039/c3cc44342f 24018656

[20] Wang QM; Shen D; Bülow M; Lau ML; Deng S; Fitch FR; Lemcoff NO; Semanscin J Metallo-organic molecular sieve for gas separation and purification. Microporous Mesoporous Mater. 2002, 55, 217–230. [CrossRef]

[21] Britt D; Furukawa H; Wang B; Glover TG; Yaghi OM Highly efficient separation of carbon dioxide by a metal-organic framework replete with open metal sites. Proc. Natl. Acad. Sci. USA 2009, 106, 20637–20640. [CrossRef] [PubMed] 19948967 10.1073/pnas.0909718106 PMC2791636

[22] Furukawa H; Cordova KE; O’Keeffe M; Yaghi OM The Chemistry and Applications of Metal-Organic Frameworks. Science 2013, 341, 1230444. [CrossRef] [PubMed] 10.1126/science.1230444 23990564

[23] Gao W-Y; Chrzanowski M; Ma S Metal-Metalloporphyrin Frameworks: Resurging Class of Functional Materials. Chem. Soc. Rev. 2014, 43, 5841–5866. [CrossRef] [PubMed] 24676096 10.1039/c4cs00001c

[24] Li B; Zhang Y; Ma D; Ma T; Shi Z; Ma S Metal Cation Directed *de novo* Assembly of a Functionalized Guest Molecule into the Nanospace of a Metal-Organic Framework. J. Am. Chem. Soc. 2014, 136, 1202–1205. [CrossRef] [PubMed] 24428188 10.1021/ja410868r

[25] Meng L; Cheng Q; Kim C; Gao W-Y; Wojtas L; Cheng Y-S; Zaworotko MJ; Zhang XP; Ma S Crystal engineering of a microporous, catalytically active fcu topology MOF using a custom-designed metalloporphyrin linker. Angew. Chem. Int. Ed. 2012, 51, 10082–10085. [CrossRef] [PubMed] 10.1002/anie.201205603 22952168

[26] Tranchemontagne DJ; Hunt JR; Yaghi OM Room Temperature Synthesis of Metal-Organic Framework: MOF-5, MOF-74, MOF-177, MOF-199, and IRMOF-0. Tetrahedron 2008, 64, 8553–8557. [CrossRef]

[27] Feng D; Gu Z-Y; Chen Y-P; Park J; Wei Z; Sun Y; Bosch M; Yuan S; Zhou H-C A Highly Stable Porphyrinic Zirconium Metal-Organic Framework with shp-a Topology. J. Am. Chem. Soc. 2014, 136, 17714–17717. [CrossRef] [PubMed] 25479454 10.1021/ja510525s

[28] Feng D; Wang K; Su J; Liu T-F; Park J; Wei Z; Bosch M; Yakovenko A; Zou X; Zhou H-C A Highly Stable Zeotype Mesoporous Zirconium Metal-Organic Framework with Ultralarge Pores. Angew. Chem. Int. Ed. 2014, 54, 149–154. [CrossRef] [PubMed] 10.1002/anie.201409334 25385329

[29] Queen WL; Hudson MR; Bloch ED; Mason JA; Gonzalez ML; Lee JS; Gygi D; Howe JD; Lee K; Darwish TA; Comprehensive study of carbon dioxide adsorption in the metal-organic frameworks M2(dobdc) (M = Mg, Mn, Fe, Co, Ni, Cu, Zn). Chem. Sci. 2014, 5, 4569–4581. [CrossRef]

[30] Bloch ED; Hudson MR; Mason JA; Queen WL; Zadrozny JM; Chavan S; Bordiga S; Brown CM; Long JR Reversible CO Binding Enables Tunable CO/H_2_ and CO/N_2_ Separations in Metal-Organic Frameworks with Exposed Divalent Metal Cations. J. Am. Chem. Soc. 2014, 136, 10752–10761. [CrossRef] [PubMed] 24999916 10.1021/ja505318p

[31] Caskey SR; Wong-Foy AG; Matzger AJ Dramatic Tuning of Carbon Dioxide Uptake via Metal substitution in a Coordination Polymer with Cylindrical Pores. J. Am. Chem. Soc. 2008, 130, 10870–10871. [CrossRef] [PubMed] 18661979 10.1021/ja8036096

[32] Wu H; Simmons JM; Srinivas G; Zhou W; Yildirim T Adsorption sites and binding nature of CO_2_ in prototypical metal-organic frameworks-A combined neutron diffraction and first-principles study. J. Phys. Chem. Lett. 2010, 1, 1946–1951. [CrossRef]

[33] Chui SS-Y; Lo SM-F; Charmant JPH; Orpen AG; Williams ID A Chemically Functionalizable Nanoporous Material [Cu3(TMA)2(H2O)3]n. Science 1999, 283, 1148–1150. [CrossRef] [PubMed] 10024237 10.1126/science.283.5405.1148

[34] Wong-Ng W; Kaduk JA; Siderius DL; Allen AL; Espinal L; Boyerinas BM; Levin I; Suchomel MR; Ilavsky J; Li L Reference Diffraction Patterns, Microstructure, and Pore Size Distribution for the Copper (II) benzene-1,3,5-tricarboxylate Metal Organic Framework (Cu-BTC) Compounds. Powder Diffr. 2015, 30, 2–13. [CrossRef]

[35] Bourrelly S; Serre C; Vimont A; Ramsahye NA; Maurin G; Daturi M; Filinehuk Y; Férey G; Llewellyn PL A Multidisciplary Approach to Understanding Sorption Induced Breathing in the Metal Organic Framework MIL53(Cr). In Zeolites to Porous Materials—The 40th Anniversary of International Zeolite Conference; Xu R, Gao Z, Chen J, Yan W, Eds.; Elsevier: Amsterdam, Netherlands, 2007; pp. 1008–1014.

[36] Espinal L; Wong-Ng W; Kaduk JA; Allen AJ; Snyder CR; Chiu C; Siderius DW; Li L; Cockayne E; Espinal AE Time dependent CO_2_ sorption hysteresis in a one-dimensional microporous octahedral molecular sieve. J. Am. Chem. Soc. 2012, 134, 7944–7951. [CrossRef] [PubMed] 22482879 10.1021/ja3014133

[37] Allen AJ; Espinal L; Wong-Ng W; Queen WL; Brown CM; Kline SR; Kauffman KL; Culp JT; Matranga C *In Situ* Structural Dynamics of Flexible Metal-Organic Frameworks for Selective CO_2_ Adsorption. J. Alloys Compd. 2015, 647, 24–34.

[38] Skoulidas AI Molecular Dynamics Simulations of Gas Diffusion in Metal-Organic Frameworks: Argon in CuBTC. J. Am. Chem. Soc. 2004, 126, 1356–1357. [CrossRef] [PubMed] 14759190 10.1021/ja039215+

[39] Wong-Ng W; Kaduk JA; Espinal L; Suchomel M; Allen AJ; Wu H High-Resolution Synchrotron X-ray Diffraction Study of Bis(2-methylimidazolyl)-Zinc, C8H10N4Zn (ZIF-8). Powder Diffr. 2011, 26, 234–237. [CrossRef]

[40] Wong-Ng W; Kaduk JA; Wu H; Suchomel M Synchrotron X-ray Studies of Metal-Organic Framework M2(2,5-dihydroxyterephthalte), M = (Mn,Co,Ni,Zn) (MOF74). Powder Diffr. 2012, 27, 256–262. [CrossRef]

[41] Wong-Ng W; Culp JT; Chen Y-S; Deschamps J; Marti A Flexible Metal Organic Framework {[Ni(DpBz)][Ni(CN)4]}n, DpBz=1,4-Bis(4-pyridyl)benzene) with an Unusual Ni-N Bond. Solid State Sci. 2016, 52, 1–9. [CrossRef]

[42] Zaman MB; Udachin K; Ripmeester JA; Smith MD; Zur Loye H-C Synthesis and Characterizaiton of Diverse Coordination Polymers. Linear and Zigzag Chains Involving Their Structural Transformation via Intermolecular Hydrogen-Bonded, Interpenetrating Ladders Polycatenane, and Noninterpenetrating Square Grid from Long, Rigid N,N’-Bidentate Ligands: 1,4-Bis[(x-pyridyl)ethynyl]benaene (x = 3,4). Inorg. Chem. 2005, 44, 5047–5059. [PubMed] 15998033 10.1021/ic050080d

[43] Hu JS; Shang Y-J; Yao X-Q; Qin L; Li YZ; Guo Z-J; Zheng H-G; Xue Z-L Syntheses, Structures, and Photoluminescence of Five New Metal-Organic Frameworks Based on Flexible Tetrapyridines and Aromatic Polycarboxylate Acids. Cryst. Growth Des. 2010, 10, 2676–2684. [CrossRef]

[44] Serre C Role of Solvent-Host Interactions that Lead to Very Large Swelling of Hybrid Frameworks. Science 2007, 315, 1828–1831. [CrossRef] [PubMed] 17395825 10.1126/science.1137975

[45] Cussen EJ; Claridge JB; Rosseinsky MJ; Kepert CJ Flexible Sorption and Transformation Behavior in a Microporous Metal-Organic Framework. J. Am. Chem. Soc. 2002, 124, 9574–9581. [CrossRef] [PubMed] 12167052 10.1021/ja0262737

[46] Kitagawa S; Kondo M Chemistry of Crystalline metal Complex-Assembled Compounds. Bull. Chem. Soc. Jpn. 1998, 71, 1739–1753. [CrossRef]

[47] Kitagawa S; Uemura K Dynamic Porous Properties of Coordination Polymers Inspired by Hydrogen Bonds. Chem. Soc. Rev. 2005, 34, 109–119. [CrossRef] [PubMed] 15672175 10.1039/b313997m

[48] Uemura K; Matsuda R; Kitagawa S Flexible Microporous Coordination Polymers. J. Solid State Chem. 2005, 178, 2420–2429. [CrossRef]

[49] Uemura K; Kitagawa S; Kondo M; Fukui K; Kitaura R; Chang H-C; Mizutani T Novel Flexible Frameworks of Porous Cobalt (II) Coordination Polymers That Show Selective Guest Adsorption Based on the Switching of Hydrogen-Bond Pairs of Amide Groups. Chem. Eur. J. 2002, 8, 3587–3590. [CrossRef] 10.1002/1521-3765(20020816)8:16<3586::AID-CHEM3586>3.0.CO;2-K 12203285

[50] Flexible MOFs. Berkeley Lectures on Energy: Carbon Capture and Sequestration, UC Berkeley Main Site. Available online: http://www.cchem.berkeley.edu/molsim/teaching/spring2013/CCS/Group8/ (accessed on 12 April 2014).

[51] Liu Y-Y; Couck S; Vandichel M; Grzywa M; Leus K; Biswas S; Volkmer D; Gascon J; Kapteijn F; Denayer JFM. New VIV-Based Metal-Organic Framework Having Framework Flexibility and High CO2 Adsorption Capacity. Inorg. Chem. 2013, 52, 113–120. [CrossRef] [PubMed] 23256823 10.1021/ic301338a

[52] Wu H; Reah RS; Smith DA; Trachtenberg MC; Li J Highly Selective CO_2_ Capture by a Flexible Microporous Metal–Organic Framework (MMOF) Material. Chem. Eur. J. 2010, 16, 13951–13954. [CrossRef] [PubMed] 21108262 10.1002/chem.201002683

[53] Choi H-S; Suh M Highly Selective CO2 Capture in Flexible 3-D Coordination Polymer Networks. Angew. Chem. Int. Ed. 2009, 48, 6865–6869. [CrossRef] [PubMed] 10.1002/anie.200902836 19693760

[54] Nijem N; Thissen P; Yao Y; Longo R; Roodenko K; Wu H; Zhao Y; Cho K; Li J; Langreth DC Understanding the Preferential Adsorption of CO_2_ over N_2_ in a Flexible Metal–Organic Framework. J. Am. Chem. Soc. 2011, 133, 12849–12857. [CrossRef] [PubMed] 21736366 10.1021/ja2051149

[55] Tanaka D; Nakagawa K; Higuchi M; Horike S; Kubota Y; Kobayashi TC; Takata M; Kitagawa S Kinetic Gate-Opening Process in a Flexible Porous Coordination Polymer. Angew. Chem. 2008, 120, 3978–3982. [CrossRef] 10.1002/anie.200705822 18404764

[56] Kauffman KL; Culp JT; Allen AJ; Espinal-Thielen L; Wong-Ng W; Brown TD; Goodman A; Bernardo MP; Pancoast RJ; Chirdon D Selective Adsorption of CO_2_ from Light Gas Mixture by Using a Structurally Dynamic Porous Coordination Polymer. Angew. Chem. Int. Ed. 2011, 50, 10888–10892. [CrossRef] [PubMed] 10.1002/anie.201104130 22002810

[57] Soldatov DV; Moudrakovski IL; Ratcliffe CI; Dutrisac R; Ripmeester JA Sorption of Xenon, Methane, and Organic Solvents by a Flexible Microporous Polymer Catena-Bis(Dibenzoylmethanato)-(4,4’-bipyridyl)nickel(II). Chem. Mater. 2003, 15, 4810–4818. [CrossRef]

[58] Hofmann KA; Küspert F Verbindungen von Kohlenwasserstoffen mit Metallsalzen. Z. Anorg. Allgem. Chem. 1897, 15, 204–207. [CrossRef]

[59] Niel V; Martinez-Agudo JM; Muñoz MC; Gaspar AB; Real JA Cooperative spin crossover behavior in cyanide-bridged Fe(II)-M(II) bimetallic 3-D Hofmann-like networks (M = Ni, Pd, and Pt). Inorg. Chem. 2001, 40, 3838–3839. [CrossRef] [PubMed] 11466039 10.1021/ic010259y

[60] Agustí G; Cobo S; Gaspar AB; Molná G; Moussa NO; Szilagyi PÁ; Pálfi V; Vieu C; Muñoz MC; Real JA Thermal and Light-Induced Spin Crossover Phenomena in New 3-D Hofmann-Like Microporous Metalorganic Frameworks Produced As Bulk Materials and Nanopatterned Thin Films. Chem Mater. 2008, 20, 6721–6732. [CrossRef]

[61] Culp JT; Madden C; Kauffman K; Shi F; Matranga C Screening Hofmann Compounds as CO_2_ Srobents: Nontraditional Synthetic Route to over 40 Different Pore-Functionalized and Flexible Pillard Cyanonickelates. Inorg. Chem. 2013, 52, 4205–4216. [CrossRef] [PubMed] 23541249 10.1021/ic301893p

[62] Culp JT; Smith MR; Bittner E; Bockrath B Hysteresis in the Physisorption of CO_2_ and N_2_ in a Flexible Pillard layer Nickel Cyanide. J. Am. Chem. Soc. 2008, 130, 12427–12434. [CrossRef] [PubMed] 18717562 10.1021/ja802474b

[63] Culp JT; Natesakhawat S; Smith MR; Bittner E; Matranga CS; Bockrath B Hydrogen storage Properties of Rigid tThree-dimensional Hofmann Clathrate Derivatives: The effect of Pore Size. J. Phys. Chem. 2008, 112, 7079–7083.

[64] Kauffman KL; Culp JT; Goodman A; Matranga C FT-IR Study of CO2 Adsorption in a Dynamic Copper (II) Benzoate-Pyrazine Host with CO_2_-CO_2_ Interactions in the Adsorbed State. J. Phys. Chem. C 2011, 115, 1857–1866. [CrossRef]

[65] Culp JT; Goodman AL; Chirdon D; Sankar SG; Matranga C Mechanism for the Dynamic Adsorption of CO_2_ and CH_4_ in a Flexible Linear Chain Coordination Polymer as Determined from in situ Infrared Spectroscopy. J. Phys. Chem. C 2010, 114, 2184–2191. [CrossRef]

[66] Bradshaw S; Claridge JB; Cussen EJ; Prior TJ; Rosseinsky MJ Design, Chirality, and Flexibility in Nanoporous Molecule-Based Materials. Acc. Chem. Res. 2005, 38, 273–282. [CrossRef] [PubMed] 15835874 10.1021/ar0401606

[67] Bureekaew S; Shimonura S; Kitagawa S Chemistry and application of flexible porous coordination polymers. Sci. Technol. Adv. Mater. 2008, 9. [CrossRef] 10.1088/1468-6996/9/1/014108 PMC5099803 27877934

[68] Ferey G Hybrid Porous Solids: Past, Present, Future. Chem. Soc. Rev. 2008, 37, 191–214. [CrossRef] [PubMed] 18197340 10.1039/b618320b

[69] Fletcher AJ; Thomas KM; Rosseinsky MJ Flexibility in Metal-Organic Framework Materials: Impact on Sorption Properties. J. Solid State Chem. 2005, 178, 2491–2510. [CrossRef]

[70] Kawano M; Fujita M Direct observation of crystalline-state guest exchange in coordination networks. Coord. Chem. Rev. 2007, 251, 2592–2605.

[71] Kitagawa S; Kitaura R; Noro S Functional Porous Coordination Polymers. Angew. Chem. Int. Ed. 2004, 43, 2334–2375. [CrossRef] [PubMed] 10.1002/anie.200300610 15114565

[72] Kitagawa S; Masuda R Chemistry of coordination space of porous coordination polymers. Coord. Chem. Rev. 2007, 251, 2490–2509. [CrossRef]

[73] Tanaka D; Henke A; Albrecht K; Moeller M; Nakagawa K; Kitagawa S; Groll J Rapid preparation of flexible porous coordination polymer nanocrystals with accelerated guest adsorption kinetics. Nat. Chem. 2010, 2, 410–416. [CrossRef] [PubMed] 20414244 10.1038/nchem.627

[74] Maji TK; Kitagawa S Chemistry of porous coordination polymers. Pure Appl. Chem. 2007, 79, 2155–2177. [CrossRef]

[75] Soldatov DV; Ukraintseva EA; Logvinenko A Structure and stability of a clathrate of bis (dibenzoylmethanato)-dipyridine-nickel (II) with acetone (1:2). J. Struct. Chem. 2007, 48, 938–948. [CrossRef]

[76] Choi HJ; Dinca M; Long JR Broadly Hysteretic H_2_ Adsorption in the Microporous Metal−Organic Framework Co(1,4-benzenedipyrazolate). J. Am. Chem. Soc. 2008, 130, 7848–7850. [CrossRef] [PubMed] 18512921 10.1021/ja8024092

[77] Liu Y; Her JH; Dailly A; Ramirez-Cuesta AJ; Neumann DA; Brown CM Reversible structural transition in MIL-53 with large temperature hysteresis. J. Am. Chem. Soc. 2008, 130, 11813–11818. [CrossRef] [PubMed] 18693731 10.1021/ja803669w

[78] Chandler BD; Enright GD; Udachin KA; Pawsey S; Ripmeester JA; Cramb DT; Shimizu GKH Mechanical gas capture and release in a network solid via multiple single-crystalline transformations. Nat. Mater. 2008, 7, 229–235. [CrossRef] [PubMed] 18204452 10.1038/nmat2101

[79] Maji TK; Matsuda R; Kitagawa S A flexible interpenetrating coordination framework with a bimodal porous functionality. Nat. Mater. 2007, 6, 142–148. 17259990 10.1038/nmat1827

[80] Shimomura S; Horike S; Matsuda R; Kitagawa S Guest-specific function of a flexible undulating channel in a 7,7,8,8-tetracyano-p-quinodimethane dimer-based porous coordination polymer. J. Am. Chem. Soc. 2007, 129, 10990–10991. [CrossRef] 17705386 10.1021/ja073505z

[81] Snurr RQ; Hupp JT; Nguyen ST Prospects for nanoporous metal-organic materials in advanced separations processes. AIChE J. 2004, 50, 1090–1095. [CrossRef]

[82] Llewellyn PL; Maurin G; Devic T; Lorea-Serna S; Rosenbach N; Serre C; Bourrelly S; Horeajada P; Filinchuk Y; Ferey G Prediction of the conditions for breathing of metal organic framework materials using a combination of X-ray powder diffraction, microcalorimetry, and molecular simulation. J. Am. Chem. Soc. 2008, 130, 12808–12814. [CrossRef] [PubMed] 18729451 10.1021/ja803899q

[83] Hagrman D; Hagr PJ; Zubieta J Solid-State Coordination Chemistry: The Self-Assembly of Microporous Organic–Inorganic Hybrid Frameworks Constructed from Tetrapyridylporphyrin and Bimetallic Oxide Chains or Oxide Clusters. Angew. Chem. Int. Ed. 1999, 38, 3165–3168. [PubMed] 10.1002/(sici)1521-3773(19991102)38:21<3165::aid-anie3165>3.0.co;2-o 10556890

[84] Wong-Ng W; Culp JT; Chen YS; Zavalij P; Espinal L; Siderius DW; Allen AJ; Scheins S; Matranga C Improved Synthesis and Crystal Structure of the Flexible Pillared Layer Porous Coordination Polymer: Ni(1,2-bis(4-pyridyl)ethylene)[Ni(CN)4]. CryEngComm. 2013, 15, 4684–4693. [CrossRef]

[85] Nishikiori S; Hasegawa T; Iwamoto T The crystal structures of α, ω- diaminoalkanecadmium(II) tetracyanonickelate(II)-Aromatic molecule inclusion compounds. V. Toluidine clathrates of the hosts built of the diamines, 1,4-diaminobutane, 1.5-diaminonentane, and 1,8-diaminooctane. J. Incl. Phenom. 1991, 11, 137–152. [CrossRef]

[86] Akitsu T; Einaga Y Extremely long axial Cu-N bonds in chiral One-dimensional zigzag cyanide-bricged CuII-NiII and CuII-PtII Bimetallic assemblies. Inorg. Chem. 2006, 45, 9826–9833. 17112280 10.1021/ic060783a

[87] Kürkçüoğlu GS; Yeşilel OZ; Kavlak I; Büyükgüngör O. Syntheses, spectral and thermal analyses of heteronuclear aqua(2-methylpyrazine)metal(II) complexes with tetracyanonickelate ion and crystal structure of supramolecular [Cd(H2O)(2mpz)Ni(μ-CN)4]n complex. Struct. Chem. 2008, 19, 879–888. [CrossRef]

[88] Hibble SJ; Chippindale AM; Pohl AH; Hannon AC Surprises from a simple metal-The structure and properties of nickel cyanide. Angew. Chem. Int. Ed. 2007, 46, 7116–7118. [CrossRef] [PubMed] 10.1002/anie.200701246 17683027

[89] Bondi A van der Waals Volumes and Radii. J. Phys. Chem. 1964, 68, 441–451. [CrossRef]

[90] Kung MC; Kung H IR Studies of NH_3_, Pyridine, CO, and NO Adsorbed on Transition Metal Oxides. Catal. Rev. Sci. Eng 1985, 27, 425–460. [CrossRef]

[91] Klauber C; Alvey MD; Yates T Evidence for chemisorption site selection based on an electron-donor mechanism. Chem. Phy. Lett. 1984, 106, 477–481. [CrossRef]

[92] Madey TE; Houston JE; Seabury CW; Rhodin TN The Structure of NH3 on Ni(111). J. Vac. Sci. Technol. 1981, 1892, 476–479. [CrossRef]

[93] Netzer FP; Madey TE Coadsorption-Induced Azimuthal ordering in Molecular Adsorbate Layers: H2O and NH3 on Oxgyen-Precovered Ni (111). Phy. Rev. Lett. 1981, 47, 928–931. [CrossRef]

[94] Calderón-Casado A; Barandika G; Bazán B; Urtiaga M-K; Vallcorba O; Rius J; Miravittles C; Arriortua M-I Solid-state transformation of the MOF [Ni2(bipy)1.5(PDC)2(H2O)2]·3.5H2O. CrystEngComm 2011, 13, 6831–6838. [CrossRef]

[95] Kizzie AC; Wong-Foy AG; Matzger AJ Effect of Humidity on the Performance of Microporous Coordination Polymers as Adsorbents for CO2 Capture. Langmuir 2011, 27, 6368–6373. [CrossRef] [PubMed] 21488612 10.1021/la200547k

[96] Chavan S; Vitillo JG; Groppo E; Bonino F; Lamberti C; Dietzel PDC; Bordiga S Polymer: Spectroscopic Features and Interaction Energy. J. Phys. Chem. C 2009, 113, 3292–3299.

[97] Černák J; Abboud KA Ni(bipy) 2Ni(CN)4, a new type of one-dimensional square tetracyano complex. Acta Crystallogr. 2000, C56, 783–785. [CrossRef] 10.1107/s0108270100004996 10935079

[98] APEX2 (2009.11–0). Program for Bruker CCD X-ray Diffractometer Control; Bruker AXS Inc.: Madison, WI, USA, 2009.

[99] Sheldrick GM SHELXTL, Version 6.14. Program for Solution and Refinement of Crystal Structures; Universität Göttingen: Göttingen, Germany, 2000.

[100] Lemmon EW; Huber ML A Computer Program, REFPROP (Reference Fluid Thermodynamic and Transport Properties, ver. 9.1); National Institute of Standards and Technology: Gaithersburg, MD, USA.

[101] Macrae CF; Bruno IJ; Chisholm JA; Edgington PR; McCabe P; Pidcock E; Rodriguez-Monge L; Taylor R; van de Streek J; Woodward PA Mercury, CSD 2.0—New Features for the Visualization and Investigation of Crystal Structures. J. Appl. Cryst. 2008, 41, 466–470. [CrossRef]

