# Supplementary material for: Crystallography of Representative MOFs Based on Pillared Cyanonickelate (PICNIC) Architecture
Source: Crystals (Basel). Author manuscript; Available in PMC 2026 Sep 16. (PMC13576038; doi:10.3390/cryst6090108)
Supplement: Supplementary file 1 [file NIHMS1613409-supplement-Supp1.pdf]

crystals

MDPI

Supplementary Materials: Crystallography of Representative MOFs Based on Pillared Cyanonickelate (PICNIC) Architecture

Winnie Wong-Ng, Jeffrey T. Culp and Yu-Sheng Chen

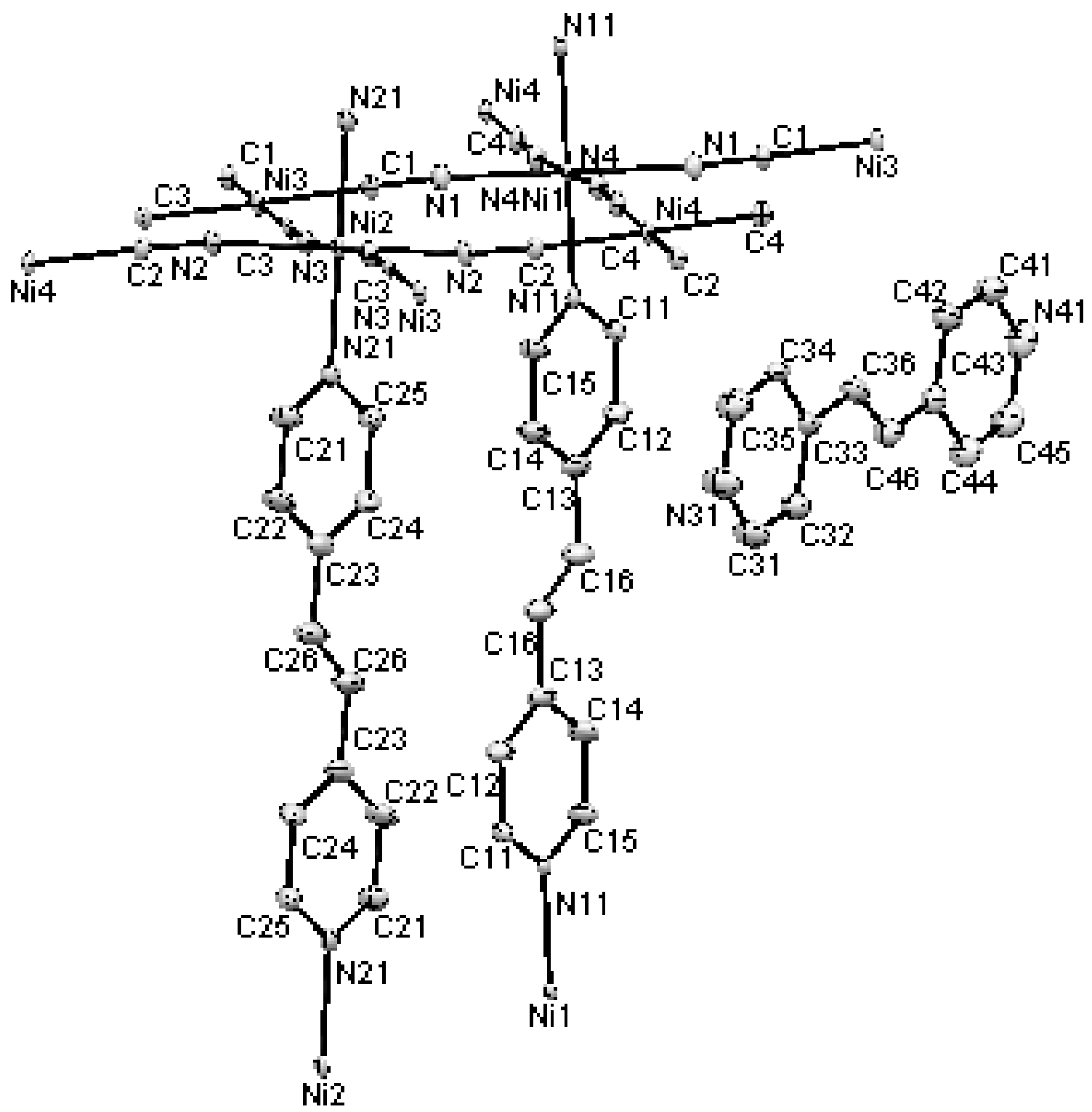

Figure S1. Labeling scheme for the Ni(bpene)[$Ni(CN)_4$] molecule.

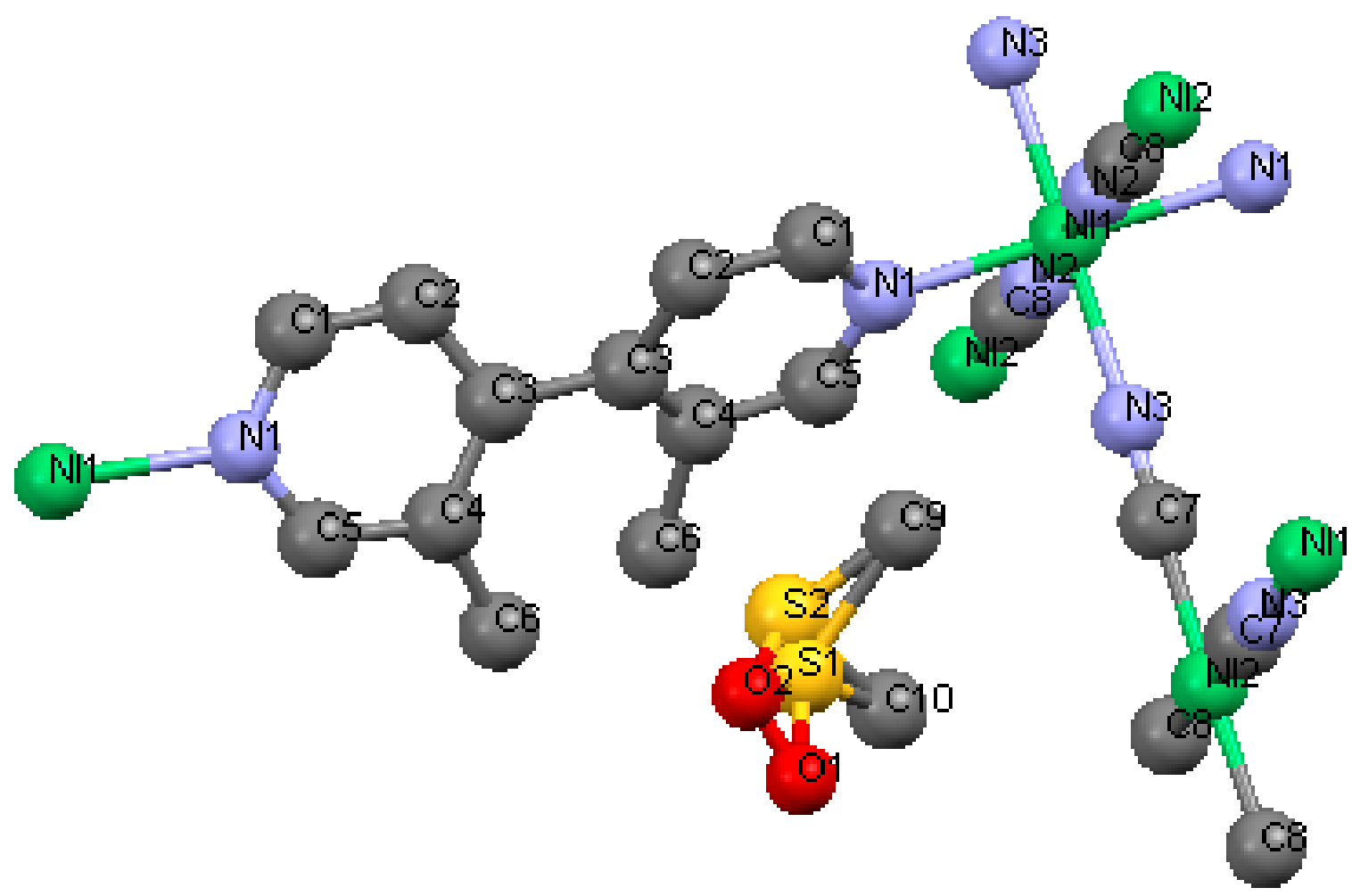

Figure S2. Labeling scheme for the Ni-BpyMe molecule.

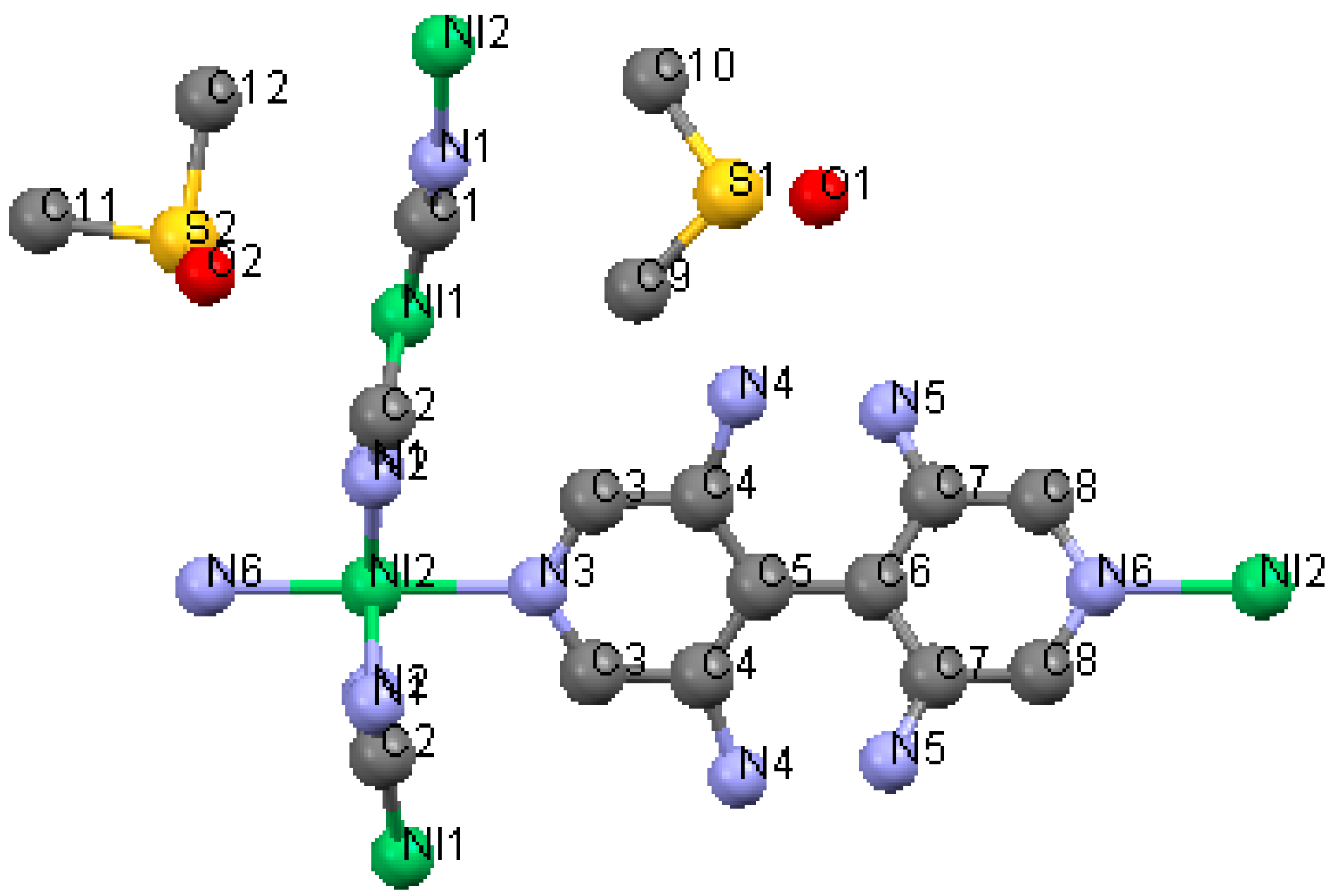

Figure S3. Labeling scheme for the Ni-BpyNH2 molecule.

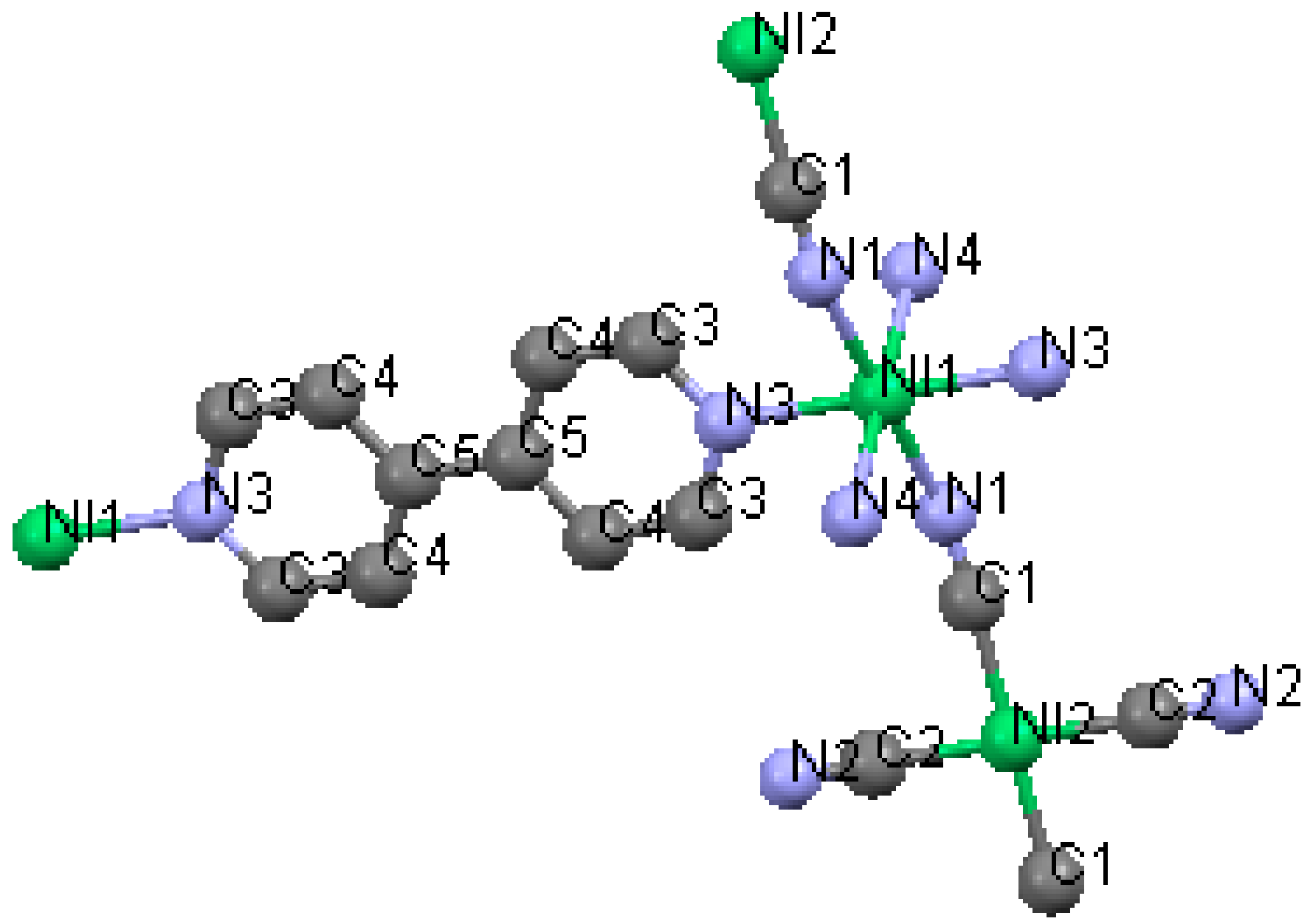

Figure S4. Labeling scheme for the Ni-Bpy molecule.

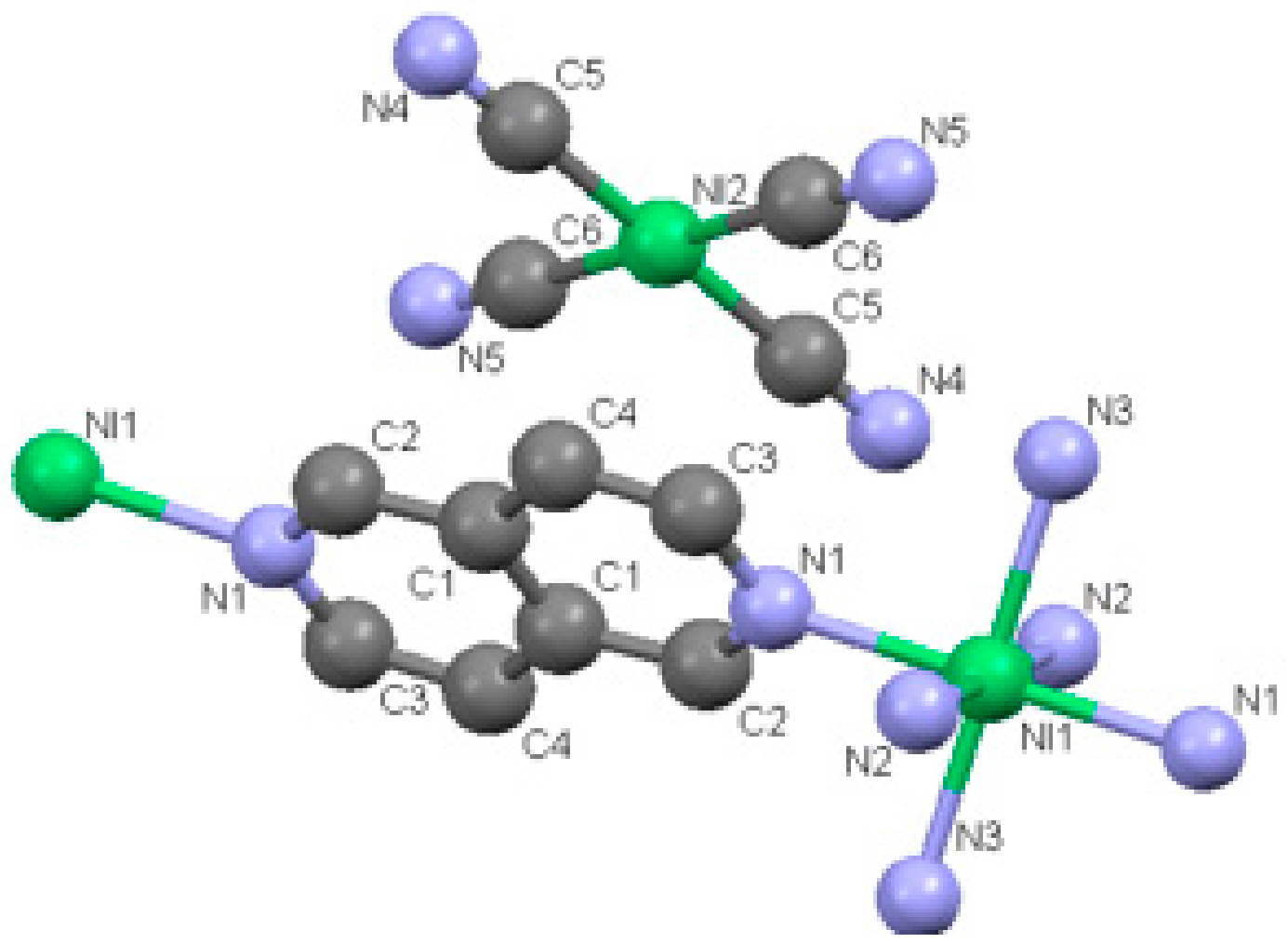

Figure S5. Labeling scheme for the Ni-Naph.

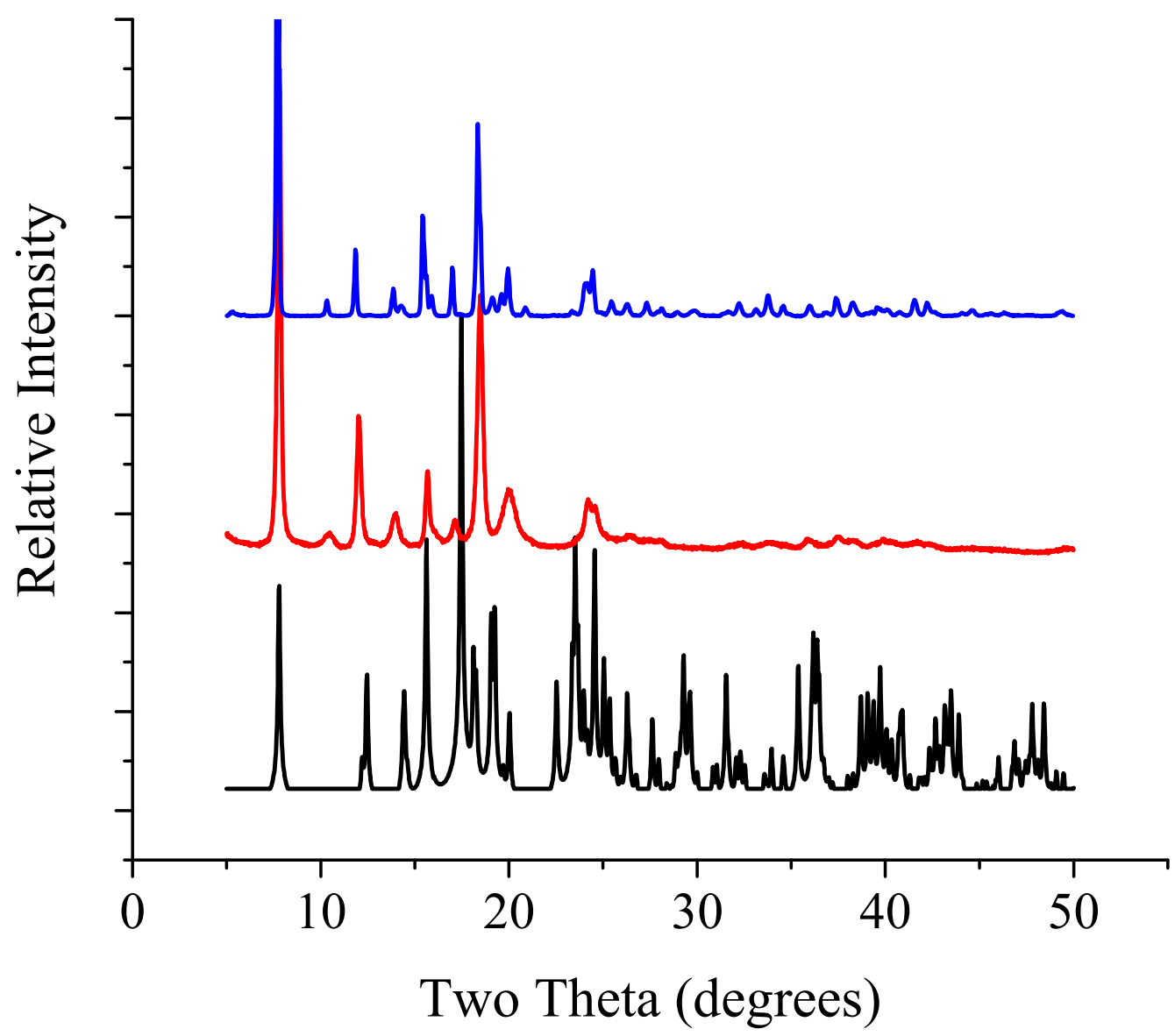

Figure S6. Comparing the powder X-ray diffraction patterns for the methanol exchanged and evacuated crystalline Ni-BpyMe sample used in the current report for gas adsorption measurements (blue) to a previously reported pattern for a polycrystalline sample of evacuated Ni-BpyMe given in reference [61] (red) and to a pattern calculated from the crystal structure of DMSO solvated Ni-BpyMe (black). Both evacuated samples give similar patterns, whereas some differences arise when comparing the solvated sample due to changes in symmetry as a result of variations in guest-loading between the samples.

Table S1. Selected bond distances (in Å) in Ni-Bpene ($Ni_2N_7C_{24}H_{35}SO_3$) [84].

Atoms	Distances	Atoms	Distances	Atoms	Distances
	Main	Components			
Ni1-N1	2.060(2)	Ni2-N2	2.055(2)		
Ni1-N4	2.066(2)	Ni2-N3	2.063(2)		
Ni1-N11	2.099(2)	Ni2-N21	2.1070(13)		
Ni3-C1	1.856(2)	Ni4-C2	1.859(2)		
Ni3-C3	1.865(2)	Ni4-C4	1.860(2)		
C1-N1	1.150(3)	C2-N2	1.154(3)		
C3-N3	1.151(3)	C4-N4	1.158(3)		
N11-C11	1.342(3)	N21-C21	1.339(2)		
N11-C15	1.343(3)	N21-C25	1.339(2)		
C11-C12	1.382(3)	C21-C22	1.380(2)		
C12-C13	1.389(4)	C22-C23	1.392(3)		
C13-C14	1.389(4)	C23-C24	1.395(3)		
C14-C15	1.381(3)	C24-C25	1.382(2)		
C16-C16	1.297(6)	C26-C26	1.319(5)		
		C23-C26	1.474(2)		
N31-C31	1.342(6)	N41-C41	1.337(5)		
N31-C35	1.327(6)	N41-C45	1.343(6)		
C31-C32	1.394(6)	C41-C42	1.401(6)		
C32-C33	1.395(6)	C42-C43	1.386(6)		
C33-C34	1.384(5)	C43-C44	1.392(6)		
C33-C36	1.479(6)	C43-C46	1.475(6)		
C34-C35	1.402(6)	C44-C45	1.393(6)		
C36-C46	1.332(8)				
	Disordered	Ligand	Components		
Ni1-Ni1A	2.118(7)				
N11A-C11A	1.337(6)	N31A-C31A	1.337(6)	N41A-C41A	1.339(6)
N11A-C15A	1.337(6)	N31A-C35A	1.335(6)	N41A-C45A	1.323(6)
C11A-C12A	1.387(6)	C31A-C32A	1.385(6)	C41A-C42A	1.392(6)
C12A-C13A	1.388(6)	C32A-C33A	1.391(6)	C42A-C43A	1.391(6)
C13A-C14A	1.385(6)	C33A-C34A	1.396(6)	C43A-C44A	1.388(6)
C13A-C16A	1.483(6)	C34A-C35A	1.396(6)	C43A-C46A	1.474(6)
C14A-C15A	1.394(6)	C36A-C46A	1.290(9)	C44A-C45A	1.398(6)
C16A-C16A	1.325(14)				
	Disordered	Solvent	(DMSO)		
S1-O1S	1.837(4)	S2-O2S	1.858(4)		
S1-C1S	1.827(4)	S2-C3S	1.507(3)		
S1-C2S	1.761(5)	S2-C4S	1.734(5)		

Table S2. Atomic coordinates (× 10^4) and equivalent isotropic displacement parameters (Å^2) (× 10^3) for Ni-BpyMe ($C_{19}H_{22}N_6Ni_2O_2S_2$). U(eq) is defined as one third of the trace of the orthogonalized U^{ij} tensor. The anisotropic displacement factor exponent takes the form: $-2\pi^2[h^2 a^{*2}U^{11} + ... + 2 h k a^* b^* U^{12}]$.

	x	y	z	U(eq)
Ni(1)	5000	0	5000	7(1)
Ni(2)	2500	5026(2)	2500	9(1)
S1a	478(11)	3099(19)	4434(10)	47(2)
O1a	−604(11)	3780(30)	3795(15)	51(6)
S2b	740(9)	2400(20)	4694(9)	36(3)
O2b	−455(13)	2510(30)	4265(17)	57(7)
N(1)	4058(4)	228(7)	5925(4)	11(1)
N(2)	6083(4)	1975(8)	5993(4)	11(1)
N(3)	4038(4)	2103(8)	3989(4)	12(1)
C(1)	3477(6)	−1241(10)	6029(5)	16(2)
C(2)	2845(6)	−1174(10)	6623(5)	16(2)
C(3)	2826(6)	477(9)	7165(6)	15(2)
C(4)	3415(7)	1996(11)	7055(7)	29(2)
C(5)	3998(6)	1808(10)	6426(6)	23(2)
C(6)	3252(11)	4020(20)	7329(11)	18(3)
C(7)	3434(5)	3198(9)	3417(5)	12(1)
C(8)	6621(5)	3116(9)	6577(5)	11(1)
C(9)	940(8)	961(17)	3834(8)	57(3)
C(10)	1331(8)	4546(15)	4323(8)	50(3)

Table S3. Anisotropic displacement parameters (Å^2) (× 10^3) for Ni-BpyMe ($C_{19}H_{22}N_6Ni_2O_2S_2$). The anisotropic displacement factor exponent takes the form: $-2\pi^2[h^2 a^{*2}U^{11} + ... + 2 h k a^* b^* U^{12}]$.

	U^{11}	U^{22}	U^{33}	U^{23}	U^{13}	U^{12}
Ni(1)	8(1)	2(1)	7(1)	1(1)	1(1)	0(1)
Ni(2)	11(1)	1(1)	9(1)	0	−2(1)	0
S1a	39(4)	56(5)	56(5)	35(5)	29(4)	15(4)
O1a	28(7)	60(14)	62(11)	−14(10)	15(7)	3(7)
S2b	29(4)	57(7)	22(4)	−4(4)	12(3)	−10(4)
O2b	39(9)	60(15)	84(13)	−14(12)	37(9)	−3(9)
N(1)	14(3)	4(3)	16(3)	3(2)	7(2)	1(2)
N(2)	11(3)	7(3)	13(3)	−1(2)	3(2)	0(2)
N(3)	11(3)	9(3)	14(3)	0(2)	4(2)	3(2)
C(1)	27(4)	9(3)	12(3)	−3(3)	8(3)	−3(3)
C(2)	20(4)	13(4)	20(4)	1(3)	12(3)	−3(3)
C(3)	22(4)	6(3)	21(4)	1(3)	12(3)	6(3)
C(4)	48(5)	11(4)	51(5)	−2(4)	41(4)	−4(4)
C(5)	35(4)	5(3)	42(5)	−7(3)	29(4)	−4(3)
C(6)	21(6)	15(6)	24(6)	−7(5)	16(5)	−11(5)
C(7)	16(3)	5(3)	14(3)	−4(3)	5(3)	−6(3)
C(8)	12(3)	8(3)	10(3)	6(3)	2(3)	5(3)
C(9)	47(6)	91(9)	31(5)	−12(5)	15(5)	−26(6)
C(10)	46(5)	64(7)	54(6)	2(5)	35(5)	13(5)

Table S4. Bond lengths [Å] for Ni-BpyMe ($C_{19}H_{22}N_6Ni_2O_2S_2$).

Atom-Atom	length [Å]
Ni(1)-N(2)#1	2.058(6)
Ni(1)-N(2)	2.058(6)
Ni(1)-N(3)	2.069(6)
Ni(1)-N(3)#1	2.069(6)
Ni(1)-N(1)	2.122(5)
Ni(1)-N(1)#1	2.122(5)
Ni(2)-C(8)#2	1.858(7)
Ni(2)-C(8)#3	1.858(7)
Ni(2)-C(7)#4	1.866(7)
Ni(2)-C(7)	1.866(7)
S1a-O1a	1.42(3)
S1a-C(10)	1.588(13)
S1a-C(9)	1.94(2)
S2b-O2b	1.45(2)
S2b-C(9)	1.651(14)
S2b-C(10)	1.88(2)
N(1)-C(5)	1.332(9)
N(1)-C(1)	1.342(8)
N(2)-C(8)	1.150(8)
N(3)-C(7)	1.154(8)
C(1)-C(2)	1.390(9)
C(2)-C(3)	1.390(9)
C(3)-C(4)	1.379(10)
C(3)-C(3)#5	1.499(13)
C(4)-C(5)	1.382(10)
C(4)-C(6)	1.519(15)
C(8)-Ni(2)#2	1.858(7)

Symmetry transformations used to generate equivalent atoms: #1 = −x + 1, −y, −z + 1; #2 = −x + 1, −y + 1, −z + 1; #3 = x − 1/2, −y + 1, z − 1/2; #4 = −x + 1/2, y, −z + 1/2; #5 = −x + 1/2, y, −z + 3/2.

Table S5. Hydrogen coordinates (× 10^4) and isotropic displacement parameters ($Å^2$) (× 10^3) for Ni-BpyMe ($C_{19}H_{22}N_6Ni_2O_2S_2$). H(4) is a theoretical value and it has an occupancy of 0.5.

	x	y	z	U(eq)
H(1)	3499	−2388	5679	19
H(2)	2432	−2242	6657	20
H(4)	3420	0315	0741	75
H(5)	4382	2881	6345	28
H(6A)	3357	4086	8087	26
H(6B)	2504	4430	6852	26
H(6C)	3791	4831	7223	26
H9Aa	1744	826	4205	85
H9Ba	722	1164	3056	85
H9Ca	587	−186	3938	85
H10Aa	1294	5742	4667	75
H10Ba	1147	4760	3552	75
H10Ca	2080	4030	4681	75

Table S6. Selected Bond angles (°) in Ni-BpyMe ($C_{19}H_{22}N_6Ni_2O_2S_2$).

Atom-Atom-Atom	Angle (°)
N(2)#1-Ni(1)-N(2)	180.0
N(2)#1-Ni(1)-N(3)	89.3(2)
N(2)-Ni(1)-N(3)	90.7(2)
N(2)#1-Ni(1)-N(3)#1	90.7(2)
N(2)-Ni(1)-N(3)#1	89.3(2)
N(3)-Ni(1)-N(3)#1	180.0
N(2)#1-Ni(1)-N(1)	90.6(2)
N(2)-Ni(1)-N(1)	89.4(2)
N(3)-Ni(1)-N(1)	88.9(2)
N(3)#1-Ni(1)-N(1)	91.1(2)
N(2)#1-Ni(1)-N(1)#1	89.4(2)
N(2)-Ni(1)-N(1)#1	90.6(2)
N(3)-Ni(1)-N(1)#1	91.1(2)
N(3)#1-Ni(1)-N(1)#1	88.9(2)
N(1)-Ni(1)-N(1)#1	180.0(2)
C(8)#2-Ni(2)-C(8)#3	89.5(4)
C(8)#2-Ni(2)-C(7)#4	177.5(3)
C(8)#3-Ni(2)-C(7)#4	89.4(2)
C(8)#2-Ni(2)-C(7)	89.4(2)
C(8)#3-Ni(2)-C(7)	177.5(3)
C(7)#4-Ni(2)-C(7)	91.9(4)
O1a-S1a-C(10)	108(2)
O1a-S1a-C(9)	115.4(9)
C(10)-S1a-C(9)	95.7(7)
O2b-S2b-C(9)	103.2(8)
O2b-S2b-C(10)	111(2)
C(9)-S2b-C(10)	95.8(6)
C(5)-N(1)-C(1)	116.1(6)
C(5)-N(1)-Ni(1)	122.9(4)
C(1)-N(1)-Ni(1)	121.0(4)
C(8)-N(2)-Ni(1)	174.3(5)
C(7)-N(3)-Ni(1)	174.9(5)
N(1)-C(1)-C(2)	123.6(6)
N(1)-C(1)-H(1)	118.2
C(2)-C(1)-H(1)	118.2
C(3)-C(2)-C(1)	119.0(6)
C(3)-C(2)-H(2)	120.5
C(1)-C(2)-H(2)	120.5
C(4)-C(3)-C(2)	117.7(6)
C(4)-C(3)-C(3)#5	124.6(5)
C(2)-C(3)-C(3)#5	117.7(4)
C(3)-C(4)-C(5)	119.1(7)
C(3)-C(4)-C(6)	124.4(8)
C(5)-C(4)-C(6)	114.6(7)
N(1)-C(5)-C(4)	124.5(7)
N(1)-C(5)-H(5)	117.7
C(4)-C(5)-H(5)	117.7
C(4)-C(6)-H(6A)	109.5
C(4)-C(6)-H(6B)	109.5
H(6A)-C(6)-H(6B)	109.5

Table S6. *Cont.*

Atom-Atom-Atom	Angle (°)
C(4)-C(6)-H(6C)	109.5
H(6A)-C(6)-H(6C)	109.5
H(6B)-C(6)-H(6C)	109.5
N(3)-C(7)-Ni(2)	177.9(6)
N(2)-C(8)-Ni(2)#2	178.9(6)
S1a-C(9)-H9Aa	109.5
S1a-C(9)-H9Ba	109.5
H9Aa-C(9)-H9Ba	109.5
S1a-C(9)-H9Ca	109.5
H9Aa-C(9)-H9Ca	109.5
H9Ba-C(9)-H9Ca	109.5
S1a-C(10)-H10Aa	109.5
S1a-C(10)-H10Ba	109.5
H10Aa-C(10)-H10Ba	109.5
S1a-C(10)-H10Ca	109.5
H10Aa-C(10)-H10Ca	109.5
H10Ba-C(10)-H10Ca	109.5

Table S7. Atomic coordinates (× 10^4) and equivalent isotropic displacement parameters (Å^2 × 10^3) for Ni-BpyNH$_2$ ($C_{18}H_{21}N_7Ni_2O_2S_2$). U(eq) is defined as one third of the trace of the orthogonalized U^{ij} tensor. The anisotropic displacement factor exponent takes the form: $-2\pi^2[h^2 a^{*2}U^{11} + ... + 2 h k a^* b^* U^{12}]$.

	x	y	z	U(eq)
Ni(1)	0	2473(1)	8(1)	9(1)
Ni(2)	2500	2299(1)	2500	7(1)
S(1)	305(4)	4309(3)	−1125(5)	99(2)
S(2)	0	1248(2)	−699(3)	94(2)
O(1)	−377(9)	4797(5)	−1019(11)	99(2)
O(2)	−952(7)	1372(5)	−370(8)	94(2)
N(1)	−1526(2)	2683(1)	−1420(2)	12(1)
N(2)	1468(2)	2306(1)	1503(2)	12(1)
N(3)	2500	3240(2)	2500	13(1)
N(4)	1905(13)	4357(6)	733(12)	21(3)
N(5)	1900(16)	5208(9)	4139(17)	38(5)
N(6)	2500	6371(2)	2500	14(1)
C(1)	−922(3)	2616(2)	−885(3)	10(1)
C(2)	896(3)	2360(2)	942(3)	11(1)
C(3)	2281(4)	3542(2)	1706(4)	22(1)
C(4)	2266(5)	4156(2)	1680(4)	30(1)
C(5)	2500	4475(3)	2500	27(1)
C(6)	2500	5129(3)	2500	28(2)
C(7)	2267(4)	5453(2)	3330(4)	28(1)
C(8)	2289(4)	6062(2)	3292(4)	22(1)
C(9)	0	3733(7)	−324(13)	160(8)
C(10)	0	3933(9)	−2226(10)	160(8)
C(11)	0	448(3)	−864(11)	95(4)
C(12)	0	1373(6)	−1999(6)	95(4)

Table S8. Anisotropic displacement parameters (Å^2) (× 10^3) for Ni-BpyNH2 ($C_{18}H_{21}N_7Ni_2O_2S_2$). The anisotropic displacement factor exponent takes the form: $-2\pi^2[h^2 a^{*2}U^{11} + ... + 2 h k a^* b^* U^{12}]$.

	**U11**	**U22**	**U33**	**U23**	**U13**	**U12**
Ni(1)	4(1)	17(1)	5(1)	2(1)	0	0
Ni(2)	5(1)	9(1)	7(1)	0	0(1)	0
S(1)	74(3)	94(3)	130(3)	−38(3)	1(2)	−16(2)
S(2)	167(5)	54(2)	60(2)	−6(2)	0	0
O(1)	74(3)	94(3)	130(3)	−38(3)	1(2)	−16(2)
O(2)	167(5)	54(2)	60(2)	−6(2)	0	0
N(1)	11(2)	16(1)	10(1)	0(1)	−1(1)	−1(1)
N(2)	7(2)	16(1)	11(1)	1(1)	0(1)	0(1)
N(3)	13(2)	7(1)	19(2)	0	5(2)	0
N(4)	35(9)	7(4)	23(7)	0(5)	−2(7)	2(5)
N(5)	41(9)	23(6)	50(9)	7(6)	10(7)	−9(6)
N(6)	13(2)	13(2)	15(2)	0	5(2)	0
C(1)	8(2)	15(1)	8(1)	2(1)	0(1)	−1(1)
C(2)	7(2)	16(1)	9(1)	2(1)	1(1)	1(1)
C(3)	31(3)	13(2)	22(2)	2(2)	−2(2)	−1(2)
C(4)	46(3)	13(2)	31(3)	6(2)	9(2)	2(2)
C(5)	39(4)	10(2)	31(3)	0	8(3)	0
C(6)	39(4)	11(2)	34(4)	0	15(4)	0
C(7)	45(3)	13(2)	26(2)	5(2)	10(2)	3(2)
C(8)	29(3)	14(2)	24(2)	2(2)	6(2)	1(2)
C(9)	159(8)	159(8)	161(8)	2(3)	0	0
C(10)	159(8)	159(8)	161(8)	2(3)	0	0
C(11)	111(11)	81(8)	94(9)	−42(7)	0	0
C(12)	111(11)	81(8)	94(9)	−42(7)	0	0

Table S9. Selected bond lengths [Å] and angles [°] for Ni-BpyNH2 ($C_{18}H_{21}N_7Ni_2O_2S_2$).

**Atom-Atom-Atom**	**Angle (°)**
Ni(1)-C(2)#1	1.861(4)
Ni(1)-C(2)	1.861(4)
Ni(1)-C(1)	1.860(4)
Ni(1)-C(1)#1	1.860(4)
Ni(2)-N(2)#2	2.048(3)
Ni(2)-N(2)	2.048(3)
Ni(2)-N(1)#3	2.067(3)
Ni(2)-N(1)#4	2.067(3)
Ni(2)-N(6)#5	2.107(5)
Ni(2)-N(3)	2.135(4)
N(1)-C(1)	1.165(3)
N(2)-C(2)	1.150(5)
N(3)-C(3)	1.332(5)
N(3)-C(3)#2	1.332(5)
N(4)-C(4)	1.48(2)
N(5)-C(7)	1.36(2)
N(6)-C(8)#2	1.336(5)
N(6)-C(8)	1.336(5)
C(3)-C(4)	1.394(6)
C(4)-C(5)	1.387(7)
C(5)-C(4)#2	1.387(7)
C(5)-C(6)	1.483(9)
C(6)-C(7)	1.404(6)
C(6)-C(7)#2	1.404(6)
C(7)-C(8)	1.382(6)
DMSO molecules	
S(1)-O(1)	1.501(8)
S(1)-C(9)	1.769(8)
S(1)-C(10)	1.801(8)
S(2)-O(2)#1	1.498(8)
S(2)-C(12)	1.816(8)
S(2)-C(11)	1.829(8)
S(1)-O(1)	1.501(8)
S(1)-C(9)	1.769(8)
S(1)-C(10)	1.801(8)
S(2)-O(2)#1	1.498(8)

Symmetry transformations used to generate equivalent atoms: #1 = −x, y, z; #2 = −x + 1/2, y + 0, −z + 1/2; #3 = x + 1/2, −y + 1/2, −z; #4 = −x, −y + 1/2, z + 1/2; #5 = x, y − 1/2, −z + 1/2; #6 = −x, −y + 1/2, z − 1/2; #7 = x, y + 1/2, −z + 1/2.

Table S10. Atomic coordinates (× 10^4) and equivalent isotropic displacement parameters (Å^2) (× 10^3) of the hydrogen atoms for Ni-BPyNH2 ($C_{18}H_{21}N_7Ni_2O_2S_2$). U(eq) is defined as one third of the trace of the orthogonalized U^{ij} tensor. H(4) and H(7) are theoretical values and they both have an occupancy of 0.75.

	x	y	z	U(eq)
H(4A)	1810	4735	630	26
H(4B)	1787	4099	275	26
H(5A)	1691	5435	4606	46
H(5B)	1871	4822	4196	46
H(3)	2129	3331	1134	27
H(4)	2090	4350	1100	26
H(7)	2100	5260	3920	26
H(8)	2144	6273	3866	26
H(9A)	119	3851	333	240
H(9B)	−636	3647	−397	240
H(9C)	350	3389	−475	240
H(10A)	122	4189	−2765	240
H(10B)	350	3578	−2293	240
H(10C)	−636	3837	−2214	240
H(11A)	0	406	−1557	143
H(11B)	533	263	−601	143
H(12A)	0	1786	−2149	143
H(12B)	533	1192	−2269	143

Table S11. Selected Bond angles (°) in Ni-BpyNH2 ($C_{18}H_{21}N_7Ni_2O_2S_2$).

Atom-Atom-Atom	Angle (°)
C(2)#1-Ni(1)-C(2)	90.1(2)
C(2)#1-Ni(1)-C(1)	88.2 (2)
C(2)-Ni(1)-C(1)	177.1(2)
C(1)-Ni(1)-C(1)#1	93.5(2)
N(2)#2-Ni(2)-N(2)	179.2(2)
N(2)#2-Ni(2)-N(1)#3	88.36(14)
N(2)-Ni(2)-N(1)#3	91.62(14)
N(1)#3-Ni(2)-N(1)#4	177.7(2)
N(2)#2-Ni(2)-N(6)#5	90.42(9)
N(1)#3-Ni(2)-N(6)#5	91.15(9)
N(2)-Ni(2)-N(3)	89.58(9)
N(1)#3-Ni(2)-N(3)	88.85(9)
N(6)#5-Ni(2)-N(3)	180.0
C(3)-N(3)-C(3)#2	118.0(5)
C(8)#2-N(6)-C(8)	116.7(5)
C(8)-N(6)-Ni(2)#7	121.7(3)
N(1)-C(1)-Ni(1)	176.4(4)
N(2)-C(2)-Ni(1)	177.4(4)
N(3)-C(3)-C(4)	122.7(5)
C(3)-C(4)-C(5)	119.8(5)
C(3)-C(4)-N(4)	109.6(7)
C(5)-C(4)-N(4)	130.4(6)
C(4)-C(5)-C(4)#2	117.1(6)
C(4)-C(5)-C(6)	121.5(3)
C(4)#2-C(5)-C(6)	121.5(3)
C(7)-C(6)-C(7)#2	116.8(6)
C(7)-C(6)-C(5)	121.6(3)
C(7)#2-C(6)-C(5)	121.6(3)
N(5)-C(7)-C(8)	116.7(10)
N(5)-C(7)-C(6)	123.5(10)
C(8)-C(7)-C(6)	119.2(5)
N(6)-C(8)-C(7)	124.1(5)
DMSO molecules	
O(1)-S(1)-C(9)	108.3(6)
O(1)-S(1)-C(10)	105.4(6)
C(9)-S(1)-C(10)	96.6(6)
O(2)-S(2)-C(12)	105.7(5)
O(2)-S(2)-C(11)	103.0(5)
C(12)-S(2)-C(11)	91.8(5)

Symmetry transformations used to generate equivalent atoms: #1 = −x, y, z; #2 = −x + 1/2, y + 0, −z + 1/2; #3 = x + 1/2, −y + 1/2, −z; #4 = −x, −y + 1/2, z + 1/2; #5 = x, y − 1/2,−z + 1/2; #6 = −x, −y + 1/2, z − 1/2; #7 = x, y + 1/2, −z + 1/2.

Table S12. Atomic coordinates (× 10^4) and equivalent isotropic displacement parameters (Å^2) (× 10^3) for Ni-Bpy ($C_7H_7N_4Ni$). U(eq) is defined as one third of the trace of the orthogonalized U^{ij} tensor. The anisotropic displacement factor exponent takes the form: $-2\pi^2[h^2 a^{*2}U^{11} + ... + 2 h k a^* b^* U^{12}]$.

	x	y	z	U(eq)
Ni(1)	0	0	0	6(1)
Ni(2)	0	5000	0	9(1)
C(4)	−300(2)	1172(2)	3702(2)	14(1)
N(4)	−2764(2)	616(2)	0	13(1)
N(1)	711(3)	2016(2)	0	12(1)
C(3)	−286(2)	1131(2)	2490(1)	15(1)
C(1)	504(4)	3168(2)	0	11(1)
C(5)	0	0	4346(2)	11(1)
N(3)	0	0	1868(3)	9(1)
N(2)	0	5000	2659(3)	21(1)
C(2)	0	5000	1648(4)	11(1)

Table S13. Anisotropic displacement parameters (Å^2) (× 10^3) for Ni-Bpy ($C_7H_7N_4Ni$). The anisotropic displacement factor exponent takes the form: $-2\pi^2[h^2 a^{*2}U^{11} + ... + 2 h k a^* b^* U^{12}]$.

	U^{11}	U^{22}	U^{33}	U^{23}	U^{13}	U^{12}
Ni(1)	7(1)	8(1)	4(1)	0	0	0(1)
Ni(2)	12(1)	8(1)	7(1)	0	0	0(1)
C(4)	18(1)	15(1)	10(1)	−2(1)	1(1)	1(1)
N(4)	12(1)	18(1)	8(1)	0	0	1(1)
N(1)	15(1)	16(1)	7(1)	0	0	−1(1)
C(3)	19(1)	15(1)	10(1)	1(1)	1(1)	1(1)
C(1)	13(1)	14(1)	6(1)	0	0	−2(1)
C(5)	7(1)	17(1)	7(1)	0	0	−1(1)
N(3)	8(2)	12(2)	8(2)	0	0	0(1)
N(2)	24(1)	26(1)	13(1)	0	0	5(1)
C(2)	11(2)	12(2)	11(2)	0	0	1(1)

Table S14. Bond lengths [Å] for Ni-Bpy ($C_7 H_7 N_4 Ni$).

Ni(1)-N(1)	2.067 (2)
Ni(1)-N(1)#1	2.067(2)
Ni(1)-N(4)	2.099(2)
Ni(1)-N(4)#1	2.099(2)
Ni(1)-N(3)	2.130(4)
Ni(1)-N(3)#1	2.130(4)
Ni(2)-C(1)#2	1.855(2)
Ni(2)-C(1)	1.855(2)
Ni(2)-C(2)#2	1.879(4)
Ni(2)-C(2)	1.879(4)
C(4)-C(3)	1.382(2)
C(4)-C(5)	1.393(2)
N(1)-C(1)	1.154(3)
C(3)-N(3)	1.344(3)
C(5)-C(4)#3	1.393(2)
C(5)-C(5)#4	1.491(4)
N(3)-C(3)#3	1.344(3)
N(2)-C(2)	1.152(5)

Symmetry transformations used to generate equivalent atoms: #1 = −x, −y, −z; #2 = −x, −y + 1, −z; #3 = −x, −y, z; #4 = −x, −y, −z + 1.

Table S15. Hydrogen coordinates (× 10^4) and isotropic displacement parameters ($Å^2$) (× 10^3) for Ni-Bpy ($C_7H_7N_4Ni$).

	x	y	z	U(eq)
H(3)	−530(30)	1940(20)	2057(19)	23(5)
H(4A)	−3340(30)	330(20)	610(19)	30(5)
H(4)	−530(30)	2040(20)	4070(20)	27(4)
H(4B)	−2860(40)	1570(30)	0	39(8)

Table S16. Selected Bond angles (°) in Ni-Bpy ($C_7H_7N_4Ni$).

Atom-Atom-Atom	Angle (°)
N(1)-Ni(1)-N(1)#1	180.0
N(1)-Ni(1)-N(4)	87.52(7)
N(1)#1-Ni(1)-N(4)	92.49(7)
N(1)-Ni(1)-N(4)#1	92.49(7)
N(1)#1-Ni(1)-N(4)#1	87.51(7)
N(4)-Ni(1)-N(4)#1	180.0
N(1)-Ni(1)-N(3)	90.0
N(1)#1-Ni(1)-N(3)	90.0
N(4)-Ni(1)-N(3)	90.0
N(4)#1-Ni(1)-N(3)	90.0
N(1)-Ni(1)-N(3)#1	90.0
N(1)#1-Ni(1)-N(3)#1	90.0
N(4)-Ni(1)-N(3)#1	90.0
N(4)#1-Ni(1)-N(3)#1	90.0
N(3)-Ni(1)-N(3)#1	180.0
C(1)#2-Ni(2)-C(1)	180.0
C(1)#2-Ni(2)-C(2)#2	90.0
C(1)-Ni(2)-C(2)#2	90.0
C(1)#2-Ni(2)-C(2)	90.0
C(1)-Ni(2)-C(2)	90.0
C(2)#2-Ni(2)-C(2)	180.0
C(3)-C(4)-C(5)	120.07(14)
C(3)-C(4)-H(4)	117.5(13)
C(5)-C(4)-H(4)	122.4(13)
Ni(1)-N(4)-H(4A)	111.7(13)
Ni(1)-N(4)-H(4B)	111(2)
H(4A)-N(4)-H(4B)	107(2)
C(1)-N(1)-Ni(1)	158.04(15)
N(3)-C(3)-C(4)	123.6(2)
N(3)-C(3)-H(3)	117.2(12)
C(4)-C(3)-H(3)	119.2(12)
N(1)-C(1)-Ni(2)	176.1(2)
C(4)-C(5)-C(4)#3	116.4(2)
C(4)-C(5)-C(5)#4	121.80(10)
C(4)#3-C(5)-C(5)#4	121.80(10)
C(3)#3-N(3)-C(3)	116.3(3)
C(3)#3-N(3)-Ni(1)	121.8(2)
C(3)-N(3)-Ni(1)	121.8(2)
N(2)-C(2)-Ni(2)	180.0

Symmetry transformations used to generate equivalent atoms: #1 = −x, −y, −z; #2 = −x, −y + 1,−z; #3 = −x, −y, z; #4 = −x, −y, −z + 1.

Table S17. Atomic coordinates (× 10^4) and equivalent isotropic displacement parameters (Å^2) (× 10^3) for Ni-naph ($C_{12}H_{18}N_{10}Ni_2$). U(eq) is defined as one third of the trace of the orthogonalized U^{ij} tensor. The anisotropic displacement factor exponent takes the form: $-2\pi^2[h^2 a^{*2}U^{11} + ... + 2hka^* b^* U^{12}]$.

	**x**	**y**	**z**	**U(eq)**
Ni(1)	5000	10000	0	9(1)
Ni(2)	0	5000	5000	10(1)
N(1)	4691(1)	7914(1)	2480(1)	10(1)
N(2)	5719(1)	11755(1)	992(1)	13(1)
N(3)	1838(1)	11188(1)	488(1)	13(1)
N(4)	1784(2)	4797(1)	1394(1)	20(1)
N(5)	−1492(2)	9081(1)	3368(1)	16(1)
C(1)	5557(1)	4761(1)	4256(1)	10(1)
C(2)	5611(1)	6175(1)	2644(1)	10(1)
C(3)	3623(1)	8363(1)	3933(1)	11(1)
C(4)	3445(1)	7100(1)	5548(1)	11(1)
C(5)	1108(2)	4852(1)	2786(1)	14(1)
C(6)	−944(1)	7519(1)	4032(1)	13(1)

Table S18. Anisotropic displacement parameters (Å^2) (× 10^3) for Ni-naph ($C_{12}H_{18}N_{10}Ni_2$). The anisotropic displacement factor exponent takes the form: $-2\pi^2[h^2 a^{*2}U^{11} + ... + 2hka^* b^* U^{12}]$.

	**U^{11}**	**U^{22}**	**U^{33}**	**U^{23}**	**U^{13}**	**U^{12}**
Ni(1)	11(1)	7(1)	7(1)	0(1)	−2(1)	−2(1)
Ni(2)	11(1)	8(1)	9(1)	−1(1)	−2(1)	−1(1)
N(1)	13(1)	9(1)	8(1)	0(1)	−3(1)	−2(1)
N(2)	17(1)	11(1)	12(1)	−2(1)	−6(1)	−2(1)
N(3)	14(1)	12(1)	12(1)	−1(1)	−3(1)	−3(1)
N(4)	22(1)	18(1)	16(1)	−6(1)	−1(1)	−2(1)
N(5)	18(1)	13(1)	16(1)	−2(1)	−5(1)	−1(1)
C(1)	11(1)	9(1)	7(1)	−1(1)	−2(1)	−3(1)
C(2)	13(1)	10(1)	7(1)	−1(1)	−2(1)	−2(1)
C(3)	13(1)	10(1)	9(1)	−1(1)	−3(1)	−1(1)
C(4)	13(1)	9(1)	8(1)	−2(1)	−2(1)	−2(1)
C(5)	15(1)	11(1)	13(1)	−2(1)	−3(1)	−1(1)
C(6)	13(1)	12(1)	11(1)	−2(1)	−3(1)	−2(1)

Table S19. Bond lengths [Å] for Ni-naph ($C_{12}H_{18}N_{10}Ni_2$).

Ni(1)-N(2)#1	2.1145(8)
Ni(1)-N(2)	2.1146(8)
Ni(1)-N(3)	2.1353(9)
Ni(1)-N(3)#1	2.1353(9)
Ni(1)-N(1)	2.1429(8)
Ni(1)-N(1)#1	2.1429(8)
Ni(2)-C(6)	1.8588(10)
Ni(2)-C(6)#2	1.8588(10)
Ni(2)-C(5)	1.8627(10)
Ni(2)-C(5)#2	1.8628(10)
N(1)-C(2)	1.3208(12)
N(1)-C(3)	1.3684(12)
N(4)-C(5)	1.1592(14)
N(5)-C(6)	1.1565(14)
C(1)-C(1)#3	1.409(2)
C(1)-C(4)#3	1.4137(13)
C(1)-C(2)	1.4147(13)
C(3)-C(4)	1.3649(14)
C(4)-C(1)#3	1.4138(13)

Symmetry transformations used to generate equivalent atoms: #1 = –x + 1, −y + 2, −z; #2 = −x, −y + 1, −z + 1; #3 = −x + 1, −y + 1, −z + 1.

Table S20. Hydrogen coordinates (× 10^4) and isotropic displacement parameters ($Å^2$) (× 10^3) for Ni-Naph ($C_{12}H_{18}N_{10}Ni_2$).

	x	y	z	U(eq)
H(2A)	6560(30)	12470(20)	190(20)	29(5)
H(2B)	4640(30)	12450(20)	1400(20)	31(5)
H(2C)	6370(30)	11060(20)	1800(30)	29(4)
H(3A)	1370(30)	11450(30)	−450(30)	37(5)
H(3B)	1560(20)	12230(20)	720(20)	26(4)
H(3C)	1030(30)	10490(20)	1310(20)	23(4)
H(2)	6400(20)	5830(20)	1640(20)	7(3)
H(3)	3010(20)	9620(20)	3750(20)	8(3)
H(4)	2660(20)	7460(20)	6530(20)	18(4)

Table S21. Selected Bond angles (°) in Ni-naph ($C_{12}H_{18}N_{10}Ni_2$).

Atom-Atom-Atom	Angle (°)
N(2)#1-Ni(1)-N(2)	180.0
N(2)#1-Ni(1)-N(3)	87.70(3)
N(2)-Ni(1)-N(3)	92.30(3)
N(2)#1-Ni(1)-N(3)#1	92.30(3)
N(2)-Ni(1)-N(3)#1	87.70(3)
N(3)-Ni(1)-N(3)#1	180.00(6)
N(2)#1-Ni(1)-N(1)	89.45(3)
N(2)-Ni(1)-N(1)	90.55(3)
N(3)-Ni(1)-N(1)	92.23(3)
N(3)#1-Ni(1)-N(1)	87.77(3)
N(2)#1-Ni(1)-N(1)#1	90.55(3)
N(2)-Ni(1)-N(1)#1	89.45(3)
N(3)-Ni(1)-N(1)#1	87.77(3)
N(3)#1-Ni(1)-N(1)#1	92.23(3)
N(1)-Ni(1)-N(1)#1	180.0
C(6)-Ni(2)-C(6)#2	180.0
C(6)-Ni(2)-C(5)	88.61(4)
C(6)#2-Ni(2)-C(5)	91.39(4)
C(6)-Ni(2)-C(5)#2	91.39(4)
C(6)#2-Ni(2)-C(5)#2	88.61(4)
C(5)-Ni(2)-C(5)#2	180.0
C(2)-N(1)-C(3)	118.52(8)
C(2)-N(1)-Ni(1)	120.44(6)
C(3)-N(1)-Ni(1)	120.97(6)
Ni(1)-N(2)-H(2A)	110.8(12)
Ni(1)-N(2)-H(2B)	112.3(12)
H(2A)-N(2)-H(2B)	109(2)
Ni(1)-N(2)-H(2C)	108.0(11)
H(2A)-N(2)-H(2C)	107(2)
H(2B)-N(2)-H(2C)	110(2)
Ni(1)-N(3)-H(3A)	109.3(12)
Ni(1)-N(3)-H(3B)	114.2(10)
H(3A)-N(3)-H(3B)	106(2)
Ni(1)-N(3)-H(3C)	116.9(11)
H(3A)-N(3)-H(3C)	104(2)
H(3B)-N(3)-H(3C)	105.4(15)
C(1)#3-C(1)-C(4)#3	118.45(11)
C(1)#3-C(1)-C(2)	118.05(10)
C(4)#3-C(1)-C(2)	123.50(8)
N(1)-C(2)-C(1)	122.91(8)
N(1)-C(2)-H(2)	120.0(10)
C(1)-C(2)-H(2)	117.0(10)
C(4)-C(3)-N(1)	123.27(8)
C(4)-C(3)-H(3)	121.3(9)
N(1)-C(3)-H(3)	115.5(9)
C(3)-C(4)-C(1)#3	118.79(8)
C(3)-C(4)-H(4)	121.0(10)
C(1)#3-C(4)-H(4)	120.2(10)
N(4)-C(5)-Ni(2)	178.65(10)
N(5)-C(6)-Ni(2)	177.38(10)

Symmetry transformations used to generate equivalent atoms: #1 = −x + 1, −y + 2, −z; #2= −x, −y + 1, −z + 1; #3 = −x + 1, −y + 1, −z + 1.

© 2016 by the authors; licensee MDPI, Basel, Switzerland. This article is an open access article distributed under the terms and conditions of the Creative Commons by Attribution (CC-BY) license (http://creativecommons.org/licenses/by/4.0/).
